# mineMS2: annotation of spectral libraries with exact fragmentation patterns

**DOI:** 10.1186/s13321-025-01051-y

**Published:** 2025-07-24

**Authors:** Alexis Delabrière, Coline Gianfrotta, Sylvain Dechaumet, Annelaure Damont, Thaïs Hautbergue, Pierrick Roger, Emilien L. Jamin, Olivier Puel, Christophe Junot, François Fenaille, Etienne A. Thévenot

**Affiliations:** 1 Université Paris-Saclay, CEA, List, Palaiseau, France; 2 Département Médicaments et Technologies pour la Santé, Université Paris-Saclay, CEA, INRAE, Gif-sur-Yvette, France; 3 MetaboHUB-IDF, National Infrastructure of Metabolomics and Fluxomics, Gif-sur-Yvette, France; 4 Toxalim (Research Centre in Food Toxicology), INRAE UMR 1331, ENVT, INP-Purpan, Toulouse University, Toulouse, France; 5 MetaboHUB-Metatoul, National Infrastructure of Metabolomics and Fluxomics, Metatoul-AXIOM, Toulouse, France; 6 Centre National de Recherche en Génomique Humaine, CEA Paris-Saclay, Institut François Jacob, Evry, France

**Keywords:** Computer-aided structure elucidation, Frequent subgraph mining, Graph theory, Mass spectrometry, Metabolomics

## Abstract

**Abstract:**

Identification is a major challenge in metabolomics due to the large structural diversity of metabolites. Tandem mass spectrometry is a reference technology for studying the fragmentation of molecules and characterizing their structure. Recent instruments can fragment large amounts of compounds in a single acquisition. The search for similarities within a collection of MS/MS spectra is a powerful approach to facilitate the identification of new metabolites. We propose an innovative *de novo* strategy for searching for exact fragmentation patterns within collections of MS/MS spectra. This approach is based on (i) a new representation of spectra as graphs of m/z differences, and (ii) an efficient frequent-subgraph mining algorithm. We demonstrate both on a spectral database from standards and on acquisitions in biological matrices that these new fragmentation patterns capture similarities that are not extracted by existing methods, and facilitate the structural interpretation of molecular network components and the elucidation of unknown spectra. The mineMS2 software is publicly available as an R package (https://github.com/odisce/mineMS2).

**Scientific contribution:**

We present an innovative strategy for structural elucidation, which extracts exact fragmentation patterns of m/z differences within collections of MS/MS spectra. The algorithms are implemented in a software library enabling efficient mining of MS/MS data and coupling to molecular networks. We show on real datasets the specific value of the patterns as fragmentation graphs for structural interpretation and *de novo* identification, and their complementarity to existing approaches.

## Introduction

Metabolomics, i.e. the global study of small molecules (metabolites) present in a given biological system, is of major interest for the molecular characterization of the phenotype and the search for biomarkers [1]. Analytical techniques such as liquid chromatography coupled to high-resolution mass spectrometry (LC-HRMS) enable the routine detection of several thousand potential metabolites in biological samples [2]. Direct chemical annotation of these compounds, however, remains limited since the precise mass can at best be traced back to the molecular formula [3, 4].

Tandem mass spectrometry (MS/MS) is a powerful approach to obtain structural information about the compound through the study of its fragmentation at various collision energies [5]. Modern instruments enable to perform simultaneous MS and MS/MS acquisitions on the same sample, by using Data Dependent or Data Independent Analysis (DDA and DIA; [6]). Direct matching to databases, however, is limited by the amount of available MS/MS data obtained from pure standards [7]. For this reason, numerous computational approaches have been developed in recent years to interpret MS/MS spectra in structural terms [8, 9, 10]. While “reverse” strategies seek to predict or generate the (sub)structure(s) from MS/MS spectra (either as molecular descriptors or as character representations such as SMILES; [11, 12, 13, 14]), “forward” strategies aim to simulate or predict the MS/MS spectrum from the compound’s structure (e.g. to enrich databases; [15, 16, 17, 18, 19, 20]). These *in silico* methods are based on heuristics, combinatorial optimization or statistical learning, or a combination of these approaches.

An important component of many of these approaches is the modelling of the fragmentation process of the compound as a graph, with vertices corresponding to fragment ions (represented by a molecular formula or a chemical structure) and edges indicating to possible fragmentation reactions (e.g. neutral losses). These fragmentation graphs both facilitate chemical interpretation [19] and improve predictions [12]. Within the full graph built by systematic bond cleavage, candidate fragmentation trees can be scored according to the matching with the experimental spectrum (number of peak explained, mass deviations, peak intensities; [21]) and the agreement with chemical rules regarding e.g. the bond dissociation energy [22, 19] or hydrogen rearrangements [18]. Such fragmentation trees can be used to retrieve molecules from compound databases [18, 19], or used by machine learning methods to predict molecular descriptors [23], which can then be used for compound retrieval [12]. As an alternative to select the fragmentation tree which best agrees with the observed spectrum, the probabilities associated to the edges from the fragmentation graph can be learned [15].

While these prediction tools are of major help for compound identification, the correct structure is still not always ranked first by these algorithms (28% of top-1 compound retrieval at the last CASMI challenge by the top performing software; [24, 25]), which is due in part to the limited size and diversity of the available spectral databases, in particular with regard to natural products. This is why, in parallel to forward and reverse prediction approaches, alternative strategies to directly mine similarities within collections of MS/MS spectra have emerged recently [26]. Importantly, such approaches do not require any information from spectral or molecular databases, nor any knowledge about molecular formulas. In molecular networks, as generated by the GNPS platform [27], each vertex is a spectrum and an edge indicates a high pairwise similarity according to the modified cosine score. While such a visualization greatly helps propagating annotations from known molecules, it does not, however, enable to extract common structures between more than two spectra. This is why methods have been developed to identify common patterns in MS/MS spectra, which may in turn provide structural information about the unknown compounds [28]. In the MS2LDA approach, text mining is used to extract sets of fragments or losses shared by several spectra [29]. Although the MS2LDA approach is very innovative, the tuning of the parameters from this probabilistic method is complex, and the software may be difficult to use outside of the web-server. GNPS and MS2LDA have been bundled together in the MolNetEnhancer software [30] thus facilitating this process. In addition, the MotifDB database was created to share motifs and their chemical annotations among the community [28]. However, the detected patterns, which are distributions of fragment and losses from the precursor, cannot be easily related to the underlying physical fragmentation process, and interpretation in a *de novo* context is not straightforward. More recently, MS/MS patterns associated to substructures have been learned by association rule mining [31]. This method, which has been shown to be complementary to MS2LDA, however depends on spectral databases for the learning of the associations.

Here, we propose an innovative combinatorial strategy to extract fragmentation patterns from MS/MS spectra collections, based on a representation of the MS/MS spectrum as a Directed Acyclic Graph (DAG). Our approach thus combines fragmentation modeling and common pattern discovery in an exact and *de novo* manner. The class of algorithms to extract recurring subgraphs from a set of graphs are the Frequent Subgraph Mining (FSM) algorithms. FSM algorithms extract all or a subset of the subgraphs from a set of graphs with a frequency above a specified threshold [32]. They have been used in chemistry (e.g. to mine molecular substructures), web, and biology (e.g. to mine protein-protein interactions; [33]). Patterns mining algorithms often rely on the growing of seed patterns, by combining two smaller size patterns, or by adding a single edge or vertex at a time. gSpan [34] and GASTON [35] are among the first and most efficient general purpose FSM algorithms [36]. These classes of algorithms are computationally intensive, and therefore specialized versions have often been proposed to mine specific classes of patterns such as trees [37], closed or maximal patterns [38], or cliques [39].

Our approach, named mineMS2, relies on a new representation of spectra as fragmentation graphs of m/z differences, and on a dedicated suite of mining algorithms for these particular graphs. Comparison of m/z differences, either directly or after alignment, have been shown recently of high value to assess the pairwise similarity between MS/MS spectra [40, 41]. Here, we further structure these m/z differences as graphs to detect common fragmentation patterns between groups of spectra. We demonstrate on various examples of spectra collections that the specific nature of the detected patterns (as being exact and structured as graphs) enables to capture similarities that are not detected by existing methods. In addition, we show the potential of coupling mineMS2 to molecular network approaches such as GNPS to help interpreting the detected components and annotate unknown spectra.

**Fig. 1:**
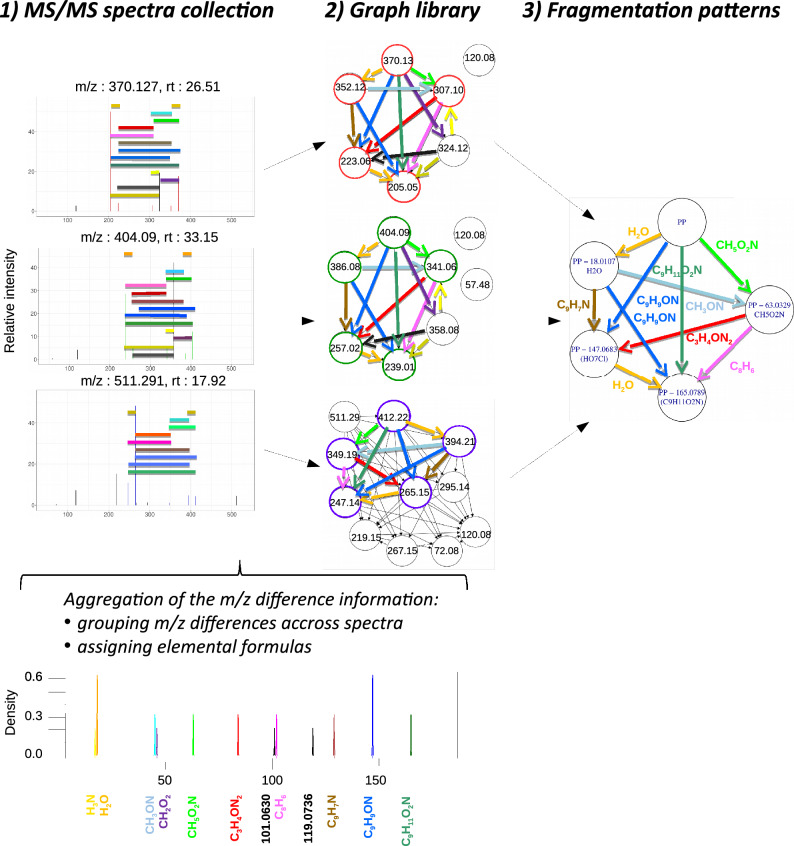
**The mineMS2 workflow:** Example of a fragmentation pattern detected in three MS/MS spectra from the *P. verrucosum* dataset; **1** a collection of MS/MS spectra is provided as input (.mgf format); **2** the spectra are converted to Directed Acyclic Graphs built on a common set of edge labels extracted from the whole spectra collection. These labels are binned m/z differences, which are converted into a molecular formula in case of a unique match; **3** maximal subgraphs (i.e. closed subgraphs) are mined at the selected frequency threshold. Here, a subgraph common to the three spectra has been identified (the peaks linked by the m/z differences from the pattern are colored in the spectra). Note that the m/z values of the pattern are shifted in the top spectrum, compared to the middle and bottom spectra

## Approach

The proposed methodology, named mineMS2, consists of two steps (Fig. 1): (1) representation of each MS/MS spectrum as a Directed Acyclic Graph (DAG), named *fragmentation graph*, whose edges correspond to the m/z differences between the peaks, and (2) mining of the set of fragmentation graphs to detect frequent subgraphs (i.e. fragmentation patterns).

**Fig. 2:**
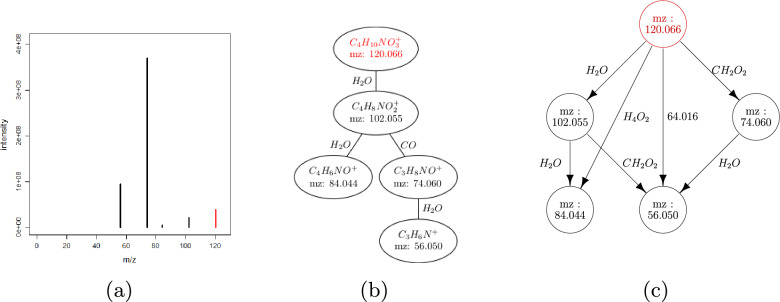
**Representation of an MS/MS spectrum as a graph.** **a** MS/MS spectrum of homoserine. **b** Corresponding fragmentation tree computed with SIRIUS [44]. **c** Proposed fragmentation graph representation as implemented by mineMS2. The precursor peak is shown in red. The fragmentation graph makes no assumption about specific fragmentation paths nor precursor formula

The required graph definitions are first introduced in Sect. 3.1. The building of the fragmentation graphs (Fig. 2), which share a common set of edge labels, is then described in Sect. 3.2. Importantly, this new graph representation is exact and makes no assumption about the fragmentation path leading to a given fragment ion, nor about the molecular formula of the precursor.

The search for common patterns of m/z differences (edges) among the fragmentation graphs is performed by an efficient Frequent Subgraph Mining (FSM) algorithm detailed in Sect. 3.4. A typical FSM algorithm generating frequent closed subgraphs relies on three main steps:

- *Candidate graph generation:* Each possible subgraph is generated sequentially once.
- *Frequency testing:* Subgraphs with frequencies below the specified threshold are discarded.
- *Closeness checking:* The pattern is checked for possible extensions with identical support, to avoid the mining of redundant patterns.

We have developed an efficient algorithm to perform these steps by taking into account several specific properties of the fragmentation graphs demonstrated in Sect. 3.3. Using one of these properties, we show that the mining of the specific class of subgraphs (i.e. possible fragmentation patterns) may be simplified to the mining of trees (Sect. 3.4.1). To ease the frequency computation step, we build a data structure storing all the frequent paths up to size *k*, that we call *k-path tree* (Sect. 3.4.3). The frequent subtrees are then generated by (i) enumerating the subtrees of the k-path tree and (ii) checking their frequency. Finally, a method is proposed to reconstruct the full subgraph from the tree and check the closeness of any mined subgraph. All the steps of the algorithm are described in Sect. 3.4.

## Methods

### Definitions

#### Definition 1

*Graph*: A graph *G* is defined as a set of vertices *V* which are connected by a set of edges *E*. It will be denoted as $G=(V,E)$. We can also note *V*(*G*) the set of vertices in *G* and *E*(*G*) the set of edges in *G*.

#### Definition 2

*Edge-labeled graph*: An **edge-labeled graph** can be represented by a 4-tuple, $G=(V,E,L,l)$, where *V* is a set of vertices, $E\subseteq V\times V$ is a set of edges, $\mathcal {L}$ is a set of labels, and $l:E\rightarrow \mathcal {L}$ is a function associating a label to each edge. If the elements of *E* are ordered pairs, the graph is called **directed**. Here, we will consider only directed edge-labeled graphs.

#### Definition 3

*Path*: A **path** is an alternating sequence of vertices and edges $v_1,e_1,v_2,\ldots ,v_{n-1},e_{n-1},v_n$, such that $v_1,\ldots , v_i,\ldots , v_n$ are vertices, and $\forall i<n,$ the edge $e_i$ goes either from $v_i$ to $v_{i+1}$ or from $v_{i+1}$ to $v_i$, and all vertices with possible exception of $v_1$ and $v_n$ are disjoints. If $v_1=v_n$, this path is called a **cycle**, otherwise it is referred as a simple path. If, for all $i < n$, the edge $e_i$ goes from $v_i$ to $v_{i+1}$, then the path is called **directed path**.

#### Definition 4

*Weakly connected graph*: A **weakly connected graph** is a graph in which there exists a path between every couple of vertices *u*, *v*.

#### Definition 5

*Root*: A graph is **rooted** if one vertex has been designated as the **root**. Here, we further assume that there is no incoming edge to the root.

#### Definition 6

*Flow graph*: A rooted graph on which there exists at least one directed path from the root to each vertex is called a **flow graph**.

#### Definition 7

*Rooted tree*: A **rooted tree** is a flow graph with a single directed path from the root to any other vertex of the graph.

#### Definition 8

*Subgraph and Supergraph*: A graph $G'=(V',E',\mathcal {L}',l')$ is a **subgraph** of a graph $G=(V,E,\mathcal {L},l)$ if the four following conditions are met:

- $$V'\subseteq V$$
- $$E'\subseteq E$$
- $$\mathcal {L}'= \mathcal {L}$$
- $$l'= l$$

*G* is called a **supergraph** of $G'$.

Moreover, if $\forall u,v \in V',(u,v)\in E \Rightarrow (u,v)\in E'$, the subgraph $G'$ is called an **induced subgraph**. This property is denoted as $G' \subseteq _eG$.

#### Definition 9

*Support*: Given a set of graphs $\mathcal {D}$ and a graph *G*, the **support** of the graph *G* in $\mathcal {D}$ is defined as: $Supp_{\mathcal {D}}(G) = |G\subseteq _eH, \forall H \in \mathcal {D}|$. It corresponds to the number of graphs from $\mathcal {D}$ containing *G*.

#### Definition 10

*Spanning tree*: A **spanning tree** of a graph *G* is a subgraph *T* such that *T* is a tree and $V(T)=V(G)$.

#### Definition 11

*Frequent Subgraph Mining*: Given a set of graphs $\mathcal {D}$ and a minimum support threshold *minSupp*, the **Frequent Subgraph Mining** problem is the extraction of every graph *G* in $\mathcal {D}$ such that:

$$\begin{aligned} Supp_{\mathcal {D}}(G) \ge minSupp \end{aligned}$$

Because every subgraph of a frequent graph is frequent, there is an exponential number of frequent graphs. Here we therefore chose to focus on the set of closed frequent subgraphs:

#### Definition 12

*Frequent closed subgraph*: A frequent subgraph $G'$ is **closed**, if there does not exist a supergraph of this graph $G'$ with similar support, i.e.,

$$\begin{aligned} \not \exists G \text { s.t. } G' \text { is a subgraph of } G \text { and } Supp_D(G)=Supp_D(G') \end{aligned}$$

#### Definition 13

*Graph Isomorphism*: An **isomorphism** between two graphs *G* and $G'$ is a bijective function $f:V(G)\rightarrow V(G')$ such that:

$$\begin{aligned} (u,v) \in E \Leftrightarrow (f(u),f(v))\in E'\text { and }l_G(u,v)=l_{G'}(f(u),f(v)) \end{aligned}$$

#### Definition 14

*Canonical form*: A **canonical form** is a standard way to represent a graph such that if two graphs *G* and *H* are isomorphic, they have the same canonical form. We define a **canonization function** as a function assigning each graph to its canonical form.

**Table 1:** Definition of the main concepts used in the mineMS2 approach

Acronym	Name	Definition
DAG	Directed Acyclic Graph	Graph with directed edges and without cycle (used to represent the MS/MS spectrum; Sect. 3.2)
AFG	Acyclic Flow Graph	Rooted DAG with at least one path from the root to each vertex (structure of the mineMS2 pattern; Sect. 3.4.1)
k-LMDF form	*k* Left-Most Depth-First canonical form	Specific canonical form of an AFG defined by picking edges in a specific order in Depth-First Search (form actually mined by mineMS2; Sect. 3.4.2)
k-path tree	*k*-path tree	Data structure to efficiently mine AFGs by storing all potentially frequent paths of size *k* or less (Sect. 3.4.3 and Fig. 3)

### Generation of fragmentation graphs

We propose to represent MS/MS spectra as Directed Acyclic Graphs (DAG) named **fragmentation graphs** (Fig. 2c; see the Table 1 for the definition of the main concepts used in mineMS2, and the Supplementary File 1, Note S1, for further details about the algorithms). Each fragment ion corresponds to a vertex, and each edge represents a discretized m/z difference between fragments. Importantly, those m/z differences arise between ions from the same fragmentation path (i.e. neutral losses), but also from alternative fragmentations. Although the latter m/z differences cannot be chemically interpreted as neutral losses, they can nevertheless provide specific information about the fragmentation of the compound. In practice, edge labels correspond to a set of disjoint m/z bins, annotated with the candidate molecular formula(s) according to the tolerance parameter (see below). The building of fragmentation graphs from a set of spectra includes three main steps:

1. **m/z difference binning: ** A set of disjoint bins is derived from the full set of m/z differences between the 15 most intense fragment ions from each spectrum (default value), using a kernel density estimation with a gaussian kernel. This subset of 15 peaks was shown to account for more than 90% of the total intensity in more than 85% of the spectra considered in this study (see the Supplementary File 1, Note S3, for a discussion about the mineMS2 parameter values). The main parameters define the bandwidth (*ppm* tolerance as well as *dmz* limit for low m/z), and the minimum frequency for each label (*f*). An additional step merges close bins (Supplementary File 1, Note S1).
2. **Formula generation: ** Possible neutral formulas are generated for all the m/z differences between 14 and 200. Putative formulas are generated as described in [42], and the existence of a planar graph for each candidate formula is checked [43]. Moreover, an m/z difference may encompass consecutive or parallel fragmentation steps, and therefore does not necessarily correspond to an existing chemical structure by itself (for example, the consecutive loss of two H_2_O molecules is plausible, whereas the H_4_O_2_ formula has no chemical meaning by itself). Thus, all combinations of formulas are also considered. For low m/z, there is often a single possible molecular formula (Fig. 2c). m/z differences between 14 and 200 Da for which no possible formula is found are discarded. All m/z differences higher than 200 are kept as such (i.e. without generating formulas).
3. **Building of the graph: ** Each MS/MS spectrum is converted to a fragmentation graph using the set of m/z bins: a vertex is created for each fragment ion, and an edge is added between vertices if the corresponding m/z difference belongs to the set of bins generated during steps 1. and 2. A last check is done to correct potential labeling error (Supplementary File 1, Note S1).

This process leads to a set of fragmentation graphs  $\mathcal {D}$, as well as a set of common labels $\mathcal {L}(\mathcal {D})$. In case multiple edges with identical label and a common source or target are found, only the closest to the middle of the m/z bin is kept (default heuristic). Such cases often result from poor MS/MS extraction.

### Properties of fragmentation graphs

Let us start with an intuition of the most important of these properties. Consider the triangle with edges H_2_O, H_2_O, H_4_O_2_ in Fig. 2c. Since all labels correspond to m/z differences, the knowledge of any two edges is sufficient to ensure that the third is present, i.e. H_2_O + H_2_O = H_4_O_2_ and H_4_O_2_ - H_2_O = H_2_O. This leads to the following property, for a set of fragmentation graphs $\mathcal {D}$ with an edge label set $\mathcal {L}(\mathcal {D})$:

#### Property 1

Consider a weakly connected graph $G=(V,E)$, subgraph of any graph of $\mathcal {D}$. Consider $G_e=(V,E/e)$ the subgraph obtained by removing any edge *e* from *G* such that $G_e$ remains weakly connected; then $Supp_\mathcal {D}(G)=Supp_\mathcal {D}(G_e)$.

It is even possible to define a second stronger property for paths. If a path *P* of size 2 composed of labels *a* and *b* is frequent, this means that between the two endpoints of *P*, *u* and *v*, there is a single m/z difference of $a+b$ with label *c*. This label *c* is frequent because for every occurrence of the path *P*, there is an occurrence of *c*. This property may be expressed more formally as:

#### Property 2

If $P=(u,x),(x,v)$ is a directed 2-edge path frequent in $\mathcal {D}$, then $\forall G \in \mathcal {D} \text { s.t. } P$ is a subgraph of *G*:

$$\begin{aligned} (u,v) \in E(G) \text { and } \exists ! c \in \mathcal {L}(\mathcal {D}) \text { s.t. } l(u,v)=c \end{aligned}$$

where $\mathcal {L}(\mathcal {D})$ is the set of edge labels from $\mathcal {D}$.

These two properties enable to build an efficient FSM algorithm. In particular, Property 2 is critical for the design of the subgraph mapping. Furthermore, Property 1 leads to the following theorem, which is key to mineMS2 FSM:

#### Theorem 1

Two induced connected subgraphs from $\mathcal {D}$, *G* and *H* are isomorphic, i.f.f there exists one spanning tree from *G*, $T_G$ and one from *H*, $T_H$ such that $T_G$ is isomorphic to $T_H$.

#### Proof

See the Supplementary File 1, Note S1. $\square$

In practice, this means that the occurrence of any spanning tree of a subgraph is equivalent to the presence of the full subgraph. The main simplification of the FSM algorithm proposed in this article therefore relies on the generation of spanning trees instead of full subgraphs. To validate that these theoretical properties indeed apply to our experimental DAGs, we evaluated the number of labeling errors in triangles within the two datasets described in this study (i.e., triangles with one edge missing or with an incorrect label). We found an error rate inferior to 1%, showing the general validity of Property 1 and 2 (Supplementary File 1, Note S1).

### Frequent subgraph mining

Deterministic and exhaustive frequent subgraph mining algorithms such as gSpan [34], SPIN [38], and GASTON [35] consist of two steps: (i) enumeration of all candidates above a frequency threshold and (ii) testing that the patterns are not isomorphic [37]. Both steps are computationally intensive.

The FSM algorithm from mineMS2 consists of the same two steps, but operates on spanning trees rather than arbitrary graphs, thus enabling to shorten the execution time. We first detail below the specific properties of the fragmentation patterns. We then present a canonical way of representing patterns, before describing a data structure that enables efficient pattern mining of the fragmentation graphs. Finally, we describe the main algorithm that computes the two steps mentioned above.

#### Patterns as Acyclic Flow Graphs (AFGs)

Since mined patterns should represent the fragmentation of chemical (sub)structures, we focus on rooted and connected subgraphs, where the root corresponds to the common substructure and the vertices to the observed fragments. Similarly to fragmentation graphs, the mined patterns are directed and acyclic, and will be denoted as **Acyclic Flow Graphs (AFG)** hereafter. These restriction on the topology of the patterns also greatly simplifies the mining problem.

As AFGs are rooted, there is a path from the root to each vertex. If an AFG is frequent, Property 2 states that there is a frequent path from the root to each vertex, and hence there is an edge from the root to each vertex. Therefore each frequent AFG contains a spanning tree consisting of all the edges outgoing from the root. This spanning tree is of depth 1. The existence of such a spanning tree for each frequent AFG allows us to define an efficient mining process using a canonical form of spanning trees and a specific data structure.

#### Canonical form of the AFGs

As a single AFG may contain distinct spanning trees, we focus on the *k* Left-Most Depth-First spanning tree as the canonical form (noted **k-LMDF**). It is obtained by performing a Depth-First Search (DFS) on an AFG *G*, such that (i) the edge with the minimum label is selected first and (ii) the search stops at depth *k*. This spanning tree is always defined for $k>0$, as there is always an edge from the root to each vertex. It will be denoted *N*(*G*) for each AFG *G*.

**Fig. 3:**
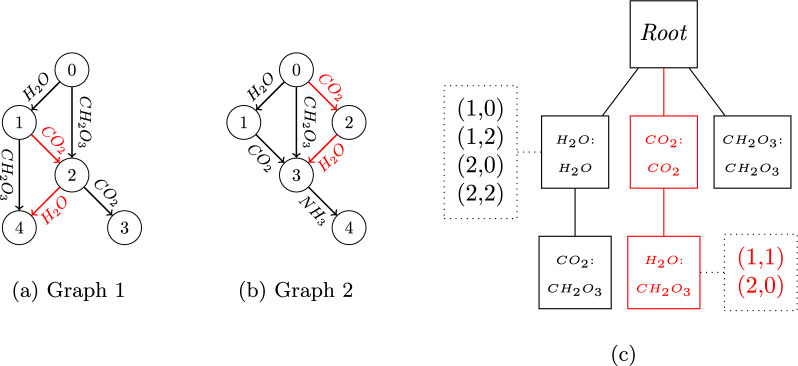
**Example of the** 2-**path tree generated from an example database** $\mathcal {D}$ **consisting of two graphs.** The 2-path tree in c) contains all the paths of size 1 or 2 within graphs **a** and **b**. The path with edge label CO_2_$\rightarrow$H_2_O is shown in red on both graphs as well as its position in the 2-path tree. At each vertex of this tree is attached: in the rectangle, *l*(*v*) (last label of *P*; top) and *h*(*v*) (edge linking both endpoints; bottom) and in the dotted box: *o*(*v*) (occurrences of *P*; some *o*(*v*) are hidden for visualization purpose)

#### Efficient data structure to mine AFGs: the k-path tree

We propose a data structure, the *k-path tree*, which stores all the potentially frequent paths (i.e. the paths satisfying Property 2) of size *k* or less in the set of graphs $\mathcal {D}$. Since the canonical form *N*(*G*) of any frequent AFG *G* is a tree of depth $\le k$, then ${\varvec{N(G)}}$ **is a subtree of the** k-path tree (hence its interest for subgraph mining). More precisely, the k-path tree is very similar to a prefix tree [45] obtained by considering all the frequent paths as a set of strings on the alphabet $\mathcal {L}(\mathcal {D})$, and by building the prefix tree of all their substrings of size *k* or less. An example of k-path tree is shown in Fig. 3. A unique vertex in the k-path tree is associated to each frequent path *P* of size *k* or less.

Consider a directed path $P=v_1,e_1,\dots ,e_{j-1},v_j$ with $j\le k$ and the associated vertex *V*(*P*) in the k-path tree. *V*(*P*) is characterized by:

- *l*: the last label of *P* ($e_{j-1}$)
- *h*: the label of the edge linking both endpoints of the path (which exists and is unique as stated in Property 2)
- *o*: the list of occurrences of *P* stored as couples $(g,v_1)$ where *g* is the index of the graph in $\mathcal {D}$ and $v_1$ is the vertex at the root of the path.

An efficient algorithm to build the k-path tree is detailed in the Supplementary File 1, Note S1. The value of *k* to be considered does not need to exceed the size of the longest path in the graphs in $\mathcal {D}$. In our case, fragmentation graphs contain at most 15 vertices, so the value of *k* can be 14 edges.

#### Mining closed Acyclic Flow Graphs using the k-path tree

All the frequent trees, and therefore spanning trees from the original database $\mathcal {D}$ are subtrees from the k-path tree. However, all the subtrees of the k-path tree are not frequent subtrees from the database $\mathcal {D}$.

Therefore in order to find all the frequent AFGs, i.e. all the patterns, in the database, we firstly enumerate all the subtrees from the k-path tree and check the following conditions:

1. Is the subtree a proper spanning tree in the fragmentation graphs?
2. Is it in k-LMDF form?
3. Is it frequent (i.e. its support is above the specified threshold)?

These conditions ensure that the subtree corresponds to the k-LMDF canonical form of an AFG.

A pattern is then rebuilt from this subtree, and its closeness is checked (see Definition 12).

These three steps will be detailed in the following paragraphs and an overview of the mining algorithm is presented in Algorithm 1.

*Enumeration of the subtrees from the k-path tree*

The enumeration is performed sequentially by considering all the frequent 1-edge paths from the k-path tree (Line 2), and by growing them, one edge at a time, similarly to [46]. The **Right-Most path extensions** are used, which ensures that each subtree can only be generated in a single way. This allows us to compute all the subtrees from the k-path tree. For each subtree, the three previously defined criteria are checked.

Criterion 1 can be illustrated as follows: consider the k-path tree showed in Fig. 3c and consider the subtree containing the root, the path Root$\rightarrow$H_2_O$\rightarrow$CO_2_ and the path Root$\rightarrow$CO_2_$\rightarrow$H_2_O. This subtree never occurs in the actual fragmentation graph 1 (Fig. 3a), because the edge labeled CO_2_ in the two paths of the k-path tree actually corresponds to the same edge in the fragmentation graph. Another example is the subtree of the k-path tree containing the root, the left-most path (Root$\rightarrow$H_2_O$\rightarrow$CO_2_) and the right-most path (Root$\rightarrow$CH_2_O_3_). This subtree occurs in the actual two fragmentation graphs (Fig. 3a, b), but it is not a spanning tree in both cases, it is a subgraph. This happens when two paths of the k-path tree lead to vertices with the same *h* value. Line 18 prevents this from happening and ensures that the criterion is fulfilled.

Criterion 2, which seeks to keep only subtrees in k-LMDF form, prevents the mining of isomorphic AFGs and is implemented at Line 16. The frequency criterion (Criterion 3) is checked on Line 11, with the parameter $\epsilon$ that sets the frequency threshold.

Additional details about these criteria are provided in the Supplementary File 1, Note S1.

**Algorithm 1:**
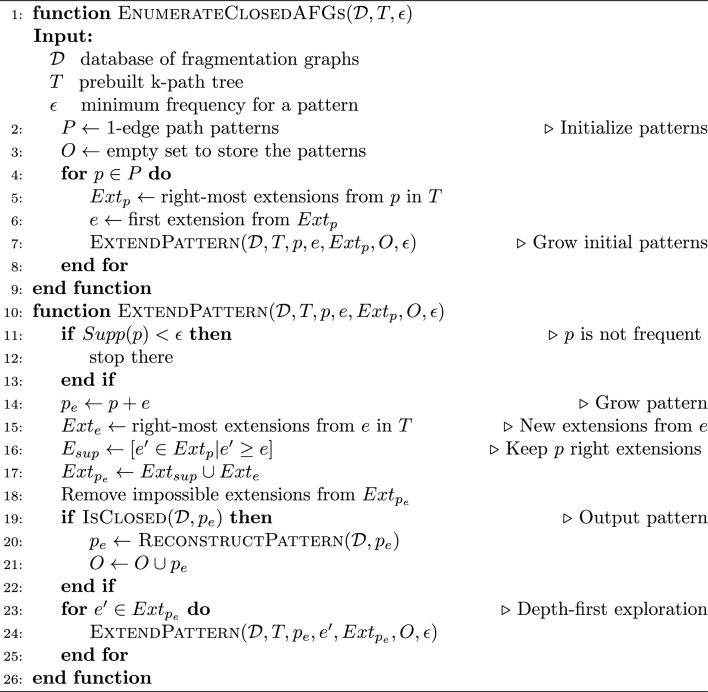
**mineMS2 Frequent Subgraph Mining algorithm**

*Closeness checking*

The closeness of the subtree needs to be checked, i.e. there must be no larger graph which includes the subtree and has the same support. As pointed in [47], to check the closeness of a graph *G*, it is sufficient to check that there does not exist a graph *H* such that *H* is the graph *G* extended with a single edge such that $Supp(H)=Supp(G)$. This condition may be further simplified because in an AFG, there is an edge from the root to any vertex. Therefore, it is sufficient to check the extensions incoming and outgoing from the root. To do so, the function isClosed (Line 19) computes two sets: $E_{in}$ the set obtained by intersecting, in every occurrence of an AFG *G* in $\mathcal {D}$, all the labels of incoming edges of the root that do not belong to *G*, and $E_{out}$ a similar set obtained by considering outgoing edges from the root. If any of these sets is not empty, the pattern is not closed.

*Pattern reconstruction*

At each extension step, if the current subtree of the k-path tree is closed, a frequent AFG, i.e. a pattern, is rebuilt from this subtree (Line 20). This is necessary because the subtree does not contain all the information of the AFG (some edges can be missing in the subtree).

This step can be done efficiently by coming back to the original set of fragmentation graphs. To reconstruct the full subgraph, the subtree in k-LMDF form is mapped on its first occurrence in $\mathcal {D}$. All the edges occurring between the mapped vertices which are not present in the k-LMDF subtree are added. These edges necessarily also occur in the other occurrences in $\mathcal {D}$ because of the Property 1.

### Implementation

The described algorithm was written in C++ using Boost, and wrapped with Rcpp into the mineMS2 R package (https://github.com/odisce/mineMS2).

All the results from this article were obtained on a Windows desktop with 64 Gb memory and a 24-core Intel Core i9-13950HX 2.2 GHz processor. The MS2LDA motifs were computed by using the MS2LDA webserver.

### Datasets

The LIMS-DB dataset consists in 622 MS/MS spectra from pure commercial standards (one spectrum per compound) acquired with a High Performance Liquid Chromatography (HPLC) system (Hypersil Gold C18 column; Thermo Fisher Scientific) coupled to a Q-Exactive Orbitrap (Thermo Fisher Scientific) operated in the positive ionization mode at a normalized collision energy (NCE) of 10% (HCD mode) and a resolution of 7500 [48, 49].

The Penicillium-DB datasets contain 52 (respectively, 51) MS/MS spectra from secondary metabolites of *Penicillium verrucosum* (respectively, *Penicillium nordicum*; one spectrum per compound) acquired on an HPLC system (Luna C18 column; Phenomenex) coupled to an LTQ Orbitrap XL hybrid (Thermo Fisher Scientific) operated in the positive ionization mode at a NCE of 20% (HCD mode) and a resolution of 7500 [50, 51].

All parameters used to process the datasets with the mineMS2, MS2LDA, and GNPS software are described in the Supplementary File 1, Note S2, and the selection of the mineMS2 parameter values is discussed in the Note S3.

## Results and discussion

As an interpretable pattern mining method, the main purpose of mineMS2 is to extract fragmentation patterns from a collection of MS/MS spectra, that correspond to structural similarities between the compounds.

### Efficiency of the mining methodology

We first evaluated the computational efficiency of the algorithms on increasing numbers of MS/MS spectra from the two main types of mass spectrometers, namely quadrupole Time-Of-Flight (qTOF) and Orbitrap. Two sets of 3000 LC-HRMS/MS spectra, acquired by positive electrospray ionisation on qTOF (respectively, Orbitrap) instruments by collision-induced dissociation at 40 eV (respectively, by higher energy collisional dissociation at 35%) were extracted from the FragHub database, which integrates the main public spectral libraries [52]. The ionization mode and collision energy were selected to maximize the number of available spectra. Computations were performed on the same laptop used for all the results presented in this study (Windows desktop with 64 Gb memory and a 24-core Intel Core i9-13950HX 2.2 GHz processor; see Sect. 3).

The computation time to process 100–3000 qTOF (respectively, Orbitrap) MS/MS spectra increased from about 10 s to 1.1 h (respectively, 3.2 h; Supplementary File 1, Fig. S1). The longer execution time with Orbitrap data is due to the larger number of spectra with more than 15 fragments (885 vs. 685 with the qTOF data on the first 1000 spectra), which results in a higher number of m/z differences (1838 vs. 1206). This, in turn, impacts the mining of the fragmentation graphs (step 2 of the algorithm), but not their building (step 1; Supplementary File 1, Fig. S1). Altogether, these results demonstrate that mineMS2 can be used to process MS/MS libraries of several thousands of spectra in a few hours.

### Chemical relevance of the mineMS2 patterns

**Fig. 4:**
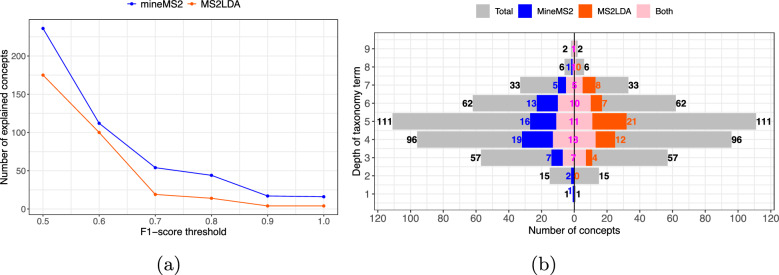
Number and distribution of ChemOnt concepts explained by mineMS2 and MS2LDA. **a** Number of explained concepts by mineMS2 (blue) and MS2LDA (orange), as a function of the F1-score threshold (the corresponding curves as a function of precision and recall are shown on Fig. S2 of the Supplementary File 1). **b** Total (grey) versus explained concepts by mineMS2 (blue) and MS2LDA (orange) at an F1-threshold of 0.6, as a function of the minimum depth of the taxonomy term. The concepts explained by both software are represented in the middle in pink

The ability of mineMS2 to extract chemically meaningful patterns was then assessed by identifying patterns explaining terms (or concepts) from the ChemOnt ontology [53] (Fig. 4). An in-house collection of 622 MS/MS spectra from pure authentic standards was used (LIMS-DB dataset, denoted $\mathcal {D}$; one spectrum per compound). These reference molecules belong to complementary chemical families (organic acids, amino acids, hormones, plant metabolites, xenobiotics), and have been selected partly according to their occurrence in human biofluids [48, 49]. The spectra from LIMS-DB were processed by mineMS2 to extract patterns common to at least two spectra. The full processing took around 20 min, and led to 5830 fragmentation patterns. In parallel, the classes from the ChemOnt ontology were retrieved for each compound by using the ClassyFire software [53]. For each taxonomy term *T*, the subset of molecules from $\mathcal {D}$ found in *T* will be denoted $\mathcal {D}(T)$ hereafter. Among the retrieved ChemOnt terms, some have exactly the same support: in this case only the one with the deeper ontology level was kept (i.e the most specific concept). Taxonomic terms containing only one molecule (i.e. such that $|\mathcal {D}(T)| = 1$) were discarded. Overall, the molecules from the LIMS-DB dataset are described by 383 taxonomic terms spread across the first 9 out of 11 levels of the ontology (as an example, *Organic oxygen compounds* and *Neuraminic acids* are taxonomy terms at level 1 and 9, respectively).

To find patterns explaining one ontology term *T*, we computed the overlap between the set of molecules described by *T*, $\mathcal {D}(T)$, and the support from pattern *P*, i.e. the molecules whose spectra contain *P*, $\mathcal {D}(P)$. The agreement between any $\mathcal {D}(T)$ and any $\mathcal {D}(P)$ was defined using the F1-score, i.e. the harmonic mean of precision and recall, which is widely used to assess the accuracy of a binary classification. Given a pattern *P* seen as the binary classifier and an ontology term *T*, the predicted label is *T* for all spectra belonging to $\mathcal {D}(P)$ and ${\overline{T}}$ for all the others. This F1-score ranges from 0 to 1 (a value of 1 indicating a perfect agreement of the two sets). All the 383 concepts were explained by at least one pattern with an F1-score threshold superior or equal to 0.2. As the F1-score threshold was decreased from 1.0 to 0.5, the number of taxonomy terms matching with at least one mineMS2 pattern increased from 16 to 236 (Fig. 4a). At a threshold of 0.6, 112 taxonomic terms were explained by at least one pattern (Fig. 4b).

Importantly, taxonomy terms were explained at almost each level of the ontology. The fact that some concepts cannot be explained accurately by mineMS2 patterns may be due in part to the very broad definition of the low level terms of the ontology. Additionally, the inclusion of (or merging with) spectra acquired at higher collision energies should enrich the graph representation of some of the compounds with new edges, and hence increase the F1-score for some concepts.

**Fig. 5:**
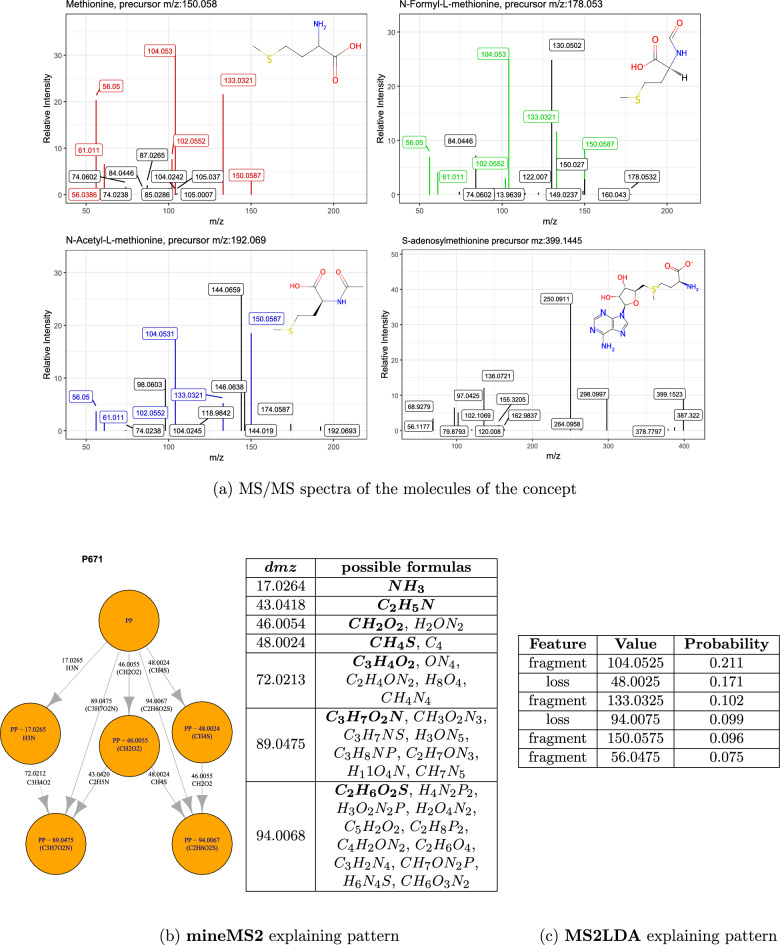
Best pattern explaining the concept *Methionine and derivatives* from mineMS2 and MS2LDA. **a** MS/MS spectra from the four LIMS-DB compounds belonging to the concept. Colored peaks on the first three spectra correspond to the nodes from the best explaining mineMS2 pattern shown below (**b**, left). m/z differences (pattern edges) are listed with the possible molecular formulas (**b**, right). In case of multiple matches, the formula with the m/z closest to the *dmz* value (bold) is shown in parentheses on the pattern. **c** Features from the MS2LDA motif with a probability above 0.05. Both mineMS2 and MS2LDA patterns explain the same 3 first spectra with an F1-score of 0.86. The patterns include two common losses, as well as 5 specific m/z differences (mineMS2) and 4 specific fragments (MS2LDA)

The explanation of the concept *Methionine and derivatives* by mineMS2 is shown as an example in Fig. 5. The ChemOnt term gathers 4 molecules of the dataset: methionine, N-acetyl-L-methionine, N-formyl-L-methionine, and S-adenosyl methionine. The mineMS2 pattern with the highest F1-score (0.86) is present in 3 out of the 4 spectra. This pattern contains in particular two m/z differences (*dmz*) that can be related to amino acids: *dmz* 17.0264 corresponding to a loss of NH_3_, and *dmz* 46.0054 corresponding to a loss of CO + H_2_O. Moreover, it also contains a m/z difference specific to the methionine and its derivatives: *dmz* 48.0024 corresponding to a loss of CH_4_S. The pattern found by mineMS2 is thus consistent with the structures of the molecules. It can be observed from the spectra (Fig. 5) that these m/z differences are detected between the same sets of peaks (between a peak at 150.0587 and a peak at 133.0321 for the loss of NH_3_ for example).

### Enriching patterns by merging spectra at multiple collision energies

We next investigated the benefit of using merged spectra acquired at multiple energies (i.e. potentially enriched in fragmentation information compared to single energy spectra) on the mineMS2 patterns. All available MS/MS spectra for the 622 standards from the LIMS-DB database (acquired on similar Orbitrap instruments) were collected, which resulted in 8 spectra per molecule on average (with a minimum of 4 and a maximum of 20) acquired over a 10% to 180% collision energy range (the most represented collision energies being 10, 20, 40 and 80%). All spectra from the same compound were merged as follows [54]: fragments with an m/z difference lower than 10 ppm, or 0.001 Da, were merged, and intensities of merged fragments were summed; the merged m/z value was computed as the intensity-weighted average.

The comparison of the mineMS2 patterns obtained with these 622 merged spectra (compared to the 622 spectra at the single 10% collision energy) showed that 48% of ChemOnt concepts were better explained by patterns from the merged spectra and, conversely, 23% were better explained with the 10% single energy spectra (Supplementary File 1, Fig. S3). The average increase (respectively, decrease) in F1-scores between multi- versus single energy spectra, was 0.13 (respectively, 0.11). This limited difference in F1-score on average may be explained by the fact that only 26% of the molecules (162 out of 622) have a cosine score below 0.5 between their merged and single energy spectrum (computed on the 15 most intense peaks, which are the one used by mineMS2).

Among the concepts better explained with the merged spectra, the *Flavonols* concept (consisting of 3 molecules) has an F1-score of 1.0 (compared to 0.4 with the single energy spectra; Supplementary File 1, Fig. S4). There is especially a gain in precision (0.3–1.0) because the pattern found with the single energy spectra contains only one m/z difference (166.025) that is present in many other molecules. In contrast, the best explaining pattern of the *Delta-5-steroids* concept (also consisting of 3 molecules) has an F1-score of 0.8 (compared to 1 in the single energy library; Supplementary File 1, Fig. S4). In this concept, two molecules are isomers and contain the pattern found with merged or single energy spectra, whereas the structure of the third molecule (pregnenolone) includes a specific acetyl group. In contrast to the single energy spectra, no motif common to all three molecules is found with merged spectra. This is due to the fact that although the two fragments of the motif found with the single energy spectra are indeed present in the merged spectrum of pregnenolone (m/z 199.0148 and 241.195), they are no more among the 15 most intense (19th and 34th respectively). Here, the merging with spectra of high collision energy (up to 100% in this case) thus results in less specific peaks predominating in the summed spectrum.

As expected, mineMS2 therefore performs best when spectra are enriched in informative peaks, which may be obtained by *stepped* approaches combining spectra acquired at multiple collision energies [55, 56].

### Complementarity to the MS2LDA motifs

The mineMS2 patterns were then compared to the motifs obtained with the probabilistic approach MS2LDA [29]. The number of topics was set to 1000. A slightly larger number of concepts are explained by mineMS2 compared to MS2LDA, for all F1-score thresholds tested (Fig. 4a). In particular, the number of explained concepts is higher for mineMS2 at all recall thresholds whereas MS2LDA explains more concepts at a precision above 0.7 (Supplementary File 1, Fig. S2). For the F1-score threshold with the closest number of concepts for the two methods (threshold of 0.6, Fig. 4b), a similar distribution of explained concepts is observed among the taxonomy depths. Importantly, a majority is best characterized specifically by one of the approaches (Fig. 4b).

To further explore this complementarity, we studied which concepts were best explained by each method. Interestingly, both software usually provide patterns with better precision than recall (in terms of precision, the best patterns averaged 0.7 with mineMS2 and 0.8 with MS2LDA, while recall averaged 0.5 for mineMS2 and 0.4 for MS2LDA). It means that while the pattern does not always explain all compounds from the concept of interest, it is seldom found in molecules from other concepts.

To study a large enough number of concepts, but that still can be inspected manually, we chose an F1 threshold of 0.7, corresponding to 60 explained concepts (i.e. 46, 7, and 7 best characterized by mineMS2, MS2LDA and both, respectively).

For mineMS2, we focused on the 7 concepts with the best F1-scores, with a depth of taxonomy above 3 and a number of molecules above 3. A detailed description of the 7 concepts best explained by mineMS2 and MS2LDA is available in the Supplementary Files 2 and 3, respectively. As expected, mineMS2 detects m/z differences that MS2LDA does not consider because they are not neutral losses but differences between peaks from two distinct fragmentation paths (Supplementary File 2). It is especially the case for the three concepts of *Flavans*, *Biopterin and derivatives*, and *Aminobenzoic acids and derivatives*. For example, the *Flavans* concept, which contains 4 molecules, is fully explained by a mineMS2 pattern (F1-score of 1), but not by MS2LDA (F1-score of 0.67). The mineMS2 pattern includes a m/z difference of 24 in m/z that corresponds to the formula C_2_. This m/z difference is most likely derived from two fragmentation paths (see Supplementary File 1, Fig. S5), and it is particularly frequent in the dataset. It is the sixth most frequent m/z difference out of the 1,549 contained in the dataset, with a number of occurrences of 335. The comprehensive analysis of all m/z differences by mineMS2 also helps to prevent the grouping of unrelated molecules, as in the *Aminobenzoic acids and derivatives* concept: while mineMS2 detects a rich pattern which is specific to all 3 molecules, the MS2LDA motif is limited to one fragment (m/z 138.058), that is indeed present in the 3 molecules of the concept but also in 7 weakly related compounds (trigonelline, pyridylacetic acids, N-acetyl-D-glucosamine, N-acetylmannosamine).

In contrast to mineMS2, the MS2LDA approach takes into account the fragments (in addition to neutral losses): such fragment features are of high importance for the 3 concepts better explained by MS2LDA (*6-aminopurines*, *Hypoxanthines* and *Serine and derivatives*; Supplementary File 3). For instance, for *6-aminopurines*, MS2LDA finds a fragment corresponding to adenine (m/z 136.062), which is present in 8 out of 10 molecules of the concept. In contrast, the m/z differences of the mineMS2 pattern are present in only 6 out of 10 compounds. For the 2 other concepts, mineMS2 is sometimes too specific, i.e. the mineMS2 pattern contains many m/z differences specific to a subclass of the concept, and MS2LDA has a better recall in this case (e.g. *Dihydroxy bile acids, alcohols and derivatives*; Supplementary File 3). Interestingly, the *Pentoses* concept illustrates a rare case of loss of precision by mineMS2 when merging many close m/z differences. For this concept, the best pattern found by mineMS2 contains a m/z difference that has a range of m/z values from 132.017 to 132.0475. This wide interval results in the presence of molecules very different from pentoses in the support from the pattern (N-Acetyl-D-penicillamine, propranolol, carnosol, prostaglandin A2). This happens because of the tolerance we considered for the binning of m/z differences. We selected a tolerance of 15 ppm because a lower value produced patterns which were too sensitive to very small variations in m/z. However, in some rare cases like this one, it results in too wide intervals of m/z values. Importantly, for two concepts (*Serine and derivatives*, and *Purine 2’-deoxyribonucleoside monophosphates*), the lower F1-score of the mineMS2 pattern in fact results at least partly from the inclusion in the support of one molecule which is structurally very close to the one from the concept (O-Acetyl-L-serine and dADP, respectively; Supplementary File 3).

### Evaluation of MESSAR to associate patterns with substructures

The MESSAR approach links MS/MS features with chemical substructures by association rule mining, and has been applied to automatically interpret MS2LDA motifs [31]. We therefore evaluated this strategy on the 14 mineMS2 and MS2LDA patterns previously selected as best explaining ChemOnt concepts. Although no structure suggestion was provided for the concepts, correct substructures annotations were detected. For two mineMS2 patterns (*Serine and derivatives* and *Coumaric acids* concepts) and one MS2LDA pattern (*Delta-5-steroids* concept), the proposed substructure with the best sensitivity is included in the structures of the molecules from the pattern. For 5 additional concepts for mineMS2 and 8 concepts for MS2LDA, consistent substructures could be found among the annotations albeit at lower sensitivities. These results confirm those previously obtained with MS2LDA: although the MESSAR rules are useful in some cases to associate relevant substructures with the motifs, the approaches are rather complementary [31], since MESSAR associates exact masses with specific substructures while the pattern mining approaches rely on experimental data to suggest combinations of features without any *a priori*.

### Detection of mineMS2 patterns in SIRIUS fragmentation trees

We next evaluated whether the mineMS2 patterns were reflected in SIRIUS fragmentation trees. To this end, the patterns from the LIMS-DB dataset best explaining specific ChemOnt concepts were selected (F1-score $>0.6$, depth in the taxonomy tree $\ge 3$, 95 patterns in total). For each pattern, the MS/MS spectra from the support (occurrences) were submitted to SIRIUS to compute the fragmentation trees. For each fragmentation tree, the subgraph containing the vertices from the mineMS2 pattern was extracted. Among the 95 sets of SIRIUS subgraphs, 6 (6%) consisted of connected and isomorphic subgraphs, 19 (20%) contained isomorphic subtrees without the full connectivity, and the remaining 70 (74%) included subgraphs which were not connected nor consistent (Supplementary File 1, Fig. S6). These results highlight the potential complementarity of mineMS2 and SIRIUS representations, and suggest that pattern based constraints might be useful in the future to enforce more similarity between the trees.

**Table 2:** Patterns explaining the similarities reported in the MS/MS studies of *P. verrucosum* [50] and *P. nordicum* [51]. For all similarities observed by the authors by molecular networking (described in the *Description* column), the presence of an mineMS2 (respectively, MS2LDA) pattern specific of the group of molecules is indicated by 1, or by 2 if the pattern is not consistent with the described similarity or if its support does not include all the molecules. 3 indicates that no pattern is found

MS/MS similarity	Origin	Description	Explaining patterns	
			mineMS2	MS2LDA
Ochratoxins	*Verrucosum & Nordicum*	Similar fragmentations	1	3
Quinazolines (verrucines, anacine and aurantiomide C)	*Verrucosum*	The 4 quinazolines are close on the MS/MS network and verrucines possess ions with similar m/z containing a loss of carbon monoxide and NH_3_	1	2
Verrucolones and C_6_H_8_O_3_	*Verrucosum*	3 ions with similar m/z	1	3
Isomers at m/z 511.29	*Verrucosum*	7 ions with similar m/z and losses of phenyalanine and valine	1	1
Aurantinomide C and Anacine	*Nordicum*	Ions with similar m/z	1	1
Fungisporin D and unknown	*Nordicum*	7 common ions between fungisporin D and an unknown metabolite	2	3
Fungisporins-related metabolites	*Nordicum*	Metabolites connected within in GNPS network	2	2

All patterns are detailed in the Supplementary File 1, Note S4

### Application to *de novo* identification

To assess the potential of the mineMS2 strategy in a *de novo* context, we applied our software to two MS/MS studies aiming at identifying new metabolites in *Penicillium verrucosum* and *Penicillium nordicum* (Penicillium-DB; [50, 51]). The datasets include 52 and 51 spectra respectively, with 8 spectra corresponding to common metabolites. Among the 95 metabolites, 33 molecules were identified by the authors as previously referenced metabolites. The mineMS2 analysis of the spectra collection was performed in 14 s for the *P.verrucosum* dataset (respectively, 24 s for the *P. nordicum* dataset), and resulted in 97 (respectively, 290) patterns detected in at least 2 spectra.

For all 7 fragmentation similarities reported by the authors (by manual inspection or by using the GNPS network), a graph pattern was detected by mineMS2 (Table 2). The mineMS2 patterns were also in $71\%$ of the case (5 times out of 7) fully coherent with the structural interpretation given by the authors. A detailed explanation of the patterns and their interpretation is provided in the Supplementary File 1, Note S4. As an example, a mineMS2 pattern specific to ochratoxin A and ochratoxin B was found. This is a rich pattern containing 13 m/z differences. It notably includes two m/z differences which are hallmarks of amino acids: CH_2_O_2_ and NH_3_. It also contains an m/z difference of 147.0683, which may correspond to a loss of phenylalanine residue, an amino acid present in ochratoxins. mineMS2 mining of fragmentation patterns is therefore of high interest in such fragmentation studies of biological samples to provide structural information of common m/z differences among groups of similar compounds and to facilitate the annotation of unknown spectra.

The MS/MS datasets were then analyzed by MS2LDA. Experiments with 200, 300 or 500 selected patterns were performed, and the value of 300 patterns was chosen because it provided the most consistent results with the fragmentation similarities described by the authors. In contrast to mineMS2, MS2LDA detected a pattern for only 4 similarities, and only 2 motifs were informative and coherent with the article (Table 2). As an example, no pattern specific to the ochratoxins was detected by MS2LDA.

Importantly, mineMS2 was shown to further enable the structural elucidation of additional unknowns. In the GNPS network from previous studies [50, 51], metabolites m/z 391 or m/z 393 were not systematically connected with the component of verrucines (A-B and F), anacine and aurantionmide, and metabolite m/z 416 remained unconnected. According to mineMS2, all these metabolites are connected with common losses of NH_3_ and CH_3_ON (e.g. NH_3_+CO; group of quinazolines in Supplementary File 1, Note S4: note that, in contrast to mineMS2, MS2LDA detects only the CH_3_ON loss in unidentified metabolites only). Postulating that (i) the fragment ion at m/z 130.0648 of the metabolite m/z 416 is representative of an indole moiety, (ii) its complementary fragment ion at m/z 287.1136 [C_14_H_14_N_4_O_3_+H]^+^ has a common structure with anacine, verrucines and aurantionmide, and (iii) these secondary fungal metabolites are produced by non-ribosomal peptide synthetases (NRPS; [57]), the m/z 416 metabolite could come from tryptophan with a proposed structure displayed in Fig. S7 from the Supplementary File 1. Similarly, m/z 391 and m/z 393 metabolites could come from tyrosine (Supplementary File 1, Fig. S7). The selectivity of the adenylation domain of the NRPS involved in the biosynthesis of these compounds may be low and the amino acid substrate of the enzyme may be either leucine, phenylalanine, tyrosine or tryptophan.

### Coupling to molecular networks

We have previously seen that mineMS2 helps propagating the fragmentation information within groups of similar spectra, including clusters from molecular networks. In fact, the coupling of mineMS2 to molecular networks [27] is a valuable strategy to focus on the patterns that best-explain each component of the network. Since mineMS2 works best at explaining small dense components, patterns specific to the connected components of the network, including cliques and high similarity pairs, are of particular interest. The coupling to molecular networks such as those generated by GNPS was therefore included as a feature of the mineMS2 software, as described in a dedicated vignette (the precomputed molecular network is provided as a file, in addition to the library of MS/MS spectra). The package thus enables to (1) automatically extracts connected components, cliques of a selected minimum size, and pairs of spectra of a specified minimum similarity, and (2) select the patterns that best explain these components.

This protocol was applied to the *P. nordicum* study ([51]; see the *mineMS2 coupling to gnps* vignette from the package). The GNPS network from the 51 spectra was rebuilt, with selected parameter values to obtain the more consistent network with the one reported by the authors (see Supplementary File 1, Note S2). This network contains two large connected components of respective size 21 and 11, two pairs, and 15 spectra remaining isolated. The main components to be explained (disjoint cliques of size above 3, as well as pairs with similarity above 0.9) were extracted (*findGNPSComponents* method), leading to 5 disjoint cliques, in addition to the initial 4 connected components of the network. Finally, mineMS2 patterns of highest recall for these components were selected (*findPatternsExplainingComponents* method), with the precision and size of the patterns being used to split ties (see the package vignette): patterns explaining each component were found, with a minimum recall of 0.9 and a mean F1-score of 0.89 (minimum of 0.55).

**Fig. 6:**
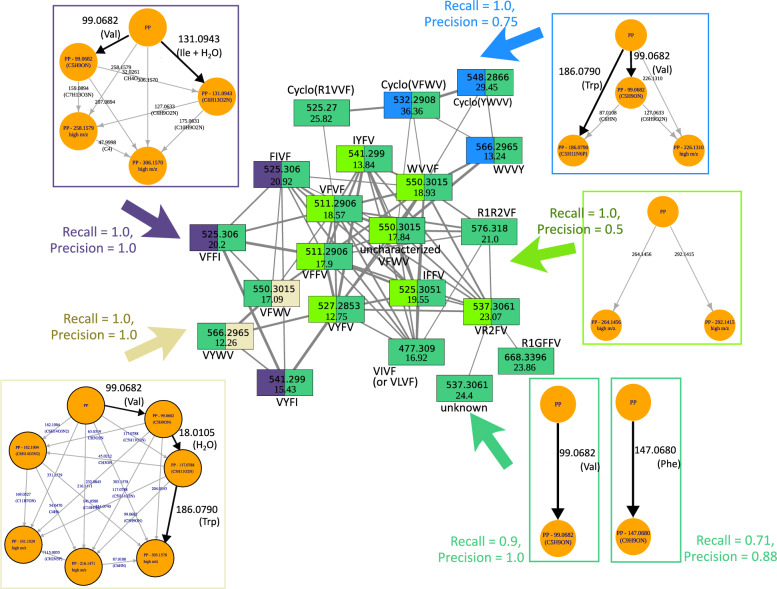
**Annotation of the fungisporin cluster from GNPS by mineMS2 patterns**. The components from the *P. nordicum* cluster explained by mineMS2 (1 global connected component, 4 disjoint cliques) and the associated patterns are displayed with specific colors. Pattern losses corresponding to specific amino acids are shown with black arrows. Each fungisporin is identified by its m/z ratio (top) and its retention time (bottom). The sequences of the identified tetrapeptides [51] are also indicated

The patterns explaining the (sub)component(s) of the larger GNPS cluster (21 spectra) were further examined (Fig. 6). This cluster consists of fungisporins, a family of tetrapeptides present in *P. verrucosum* and *P. nordicum* [51]. In particular, the whole component can be explained by two patterns (respective F1-scores of 0.95 and 0.78), each consisting of a single m/z difference (99.0682 and 147.0680, respectively), which may correspond to a respective loss of valine and phenylalanine (which are indeed included in most of the tetrapeptides). Interestingly, the 147.0680 loss is also detected in the ochratoxins by mineMS2 (in agreement with the structure of these compounds), although there is no link between the cluster and the pair in the molecular network. The study of the explained cliques further highlights losses related to additional amino acids, such as the m/z differences of 186.0790 and 131.0941 that may correspond to a loss of tryptophan and isoleucine with H_2_O, respectively. Our methodology thus enables to automatically extract the clusters from a molecular network and provide common fragmentation patterns to help interpret these clusters in terms of common m/z differences.

## Conclusion

To facilitate the identification of unknown compounds within large collections of MS/MS spectra, we propose a new approach to search for common patterns of m/z differences between spectra. This method is based, on the one hand, on a new representation of the MS/MS spectrum as a graph, independent of the knowledge of the precursor’s molecular formula, and, on the other hand, on an efficient algorithm for accurately extracting frequent subgraphs. It is implemented in the freely available mineMS2 R library, which takes the spectra file as input and returns the list of patterns, together with the associated spectra and m/z differences. It can process up to 3,000 spectra in a few hours on a standard laptop, making it compatible with global DDA-type MS/MS acquisitions.

We demonstrate both on a database of standards and in biological matrices that the patterns obtained can explain fragmentation similarities (such as those observed within a molecular network), but also chemical concepts (such as those in the ChemOnt ontology), in terms of common m/z differences organized as a fragmentation graph. Note that these m/z differences include neutral losses, but also differences between fragments belonging to distinct fragmentation paths, which may also prove specific to the fragmentation of specific molecules. The exact nature of mineMS2 motifs (i.e. all m/z differences of the motif are present in all spectra from the support), and their representation as a graph are two major advantages of mineMS2 for facilitating chemical interpretation. In particular, the natural subgraph relationship between the patterns may be used to obtain a coarse- or a fine-grain view of the similarity between the spectra, which is a major distinction with the previously described methods.

We show that mineMS2 motifs are complementary to those obtained with the MS2LDA approach [29], as 70% of the motifs best explaining ChemOnt chemical classes were found to be specific to either mineMS2 or MS2LDA. This is due to the complementary structural information provided by each type of patterns: all m/z differences for mineMS2 versus fragments and precursor losses for MS2LDA. This difference also reflects the probabilistic nature of MS2LDA (tolerant of absences of certain pattern elements in spectra) versus the deterministic nature of mineMS2 (all pattern m/z differences are present in all support spectra).

A limitation of exact pattern mining methods such as mineMS2 is the high number of patterns extracted. For the applications to large collections of spectra, coupling mineMS2 with a similarity network such as GNPS [27] enables to focus on motifs that explain groups of spectra (cliques or connected components) and facilitate their interpretation. A future approach to identifying the most informative patterns would be the development of a scoring system for patterns incorporating the frequency of the m/z difference in the selected dataset or in a reference database. This could be coupled to a target-decoy strategy by evaluating the score of randomly drawn patterns using these labels compared to the specific acquisition. In addition, strategies to facilitate the structural interpretation of the patterns will be explored in future work, e.g., by building a database of patterns of interest with a dedicated query metric. A second limitation is the need to select a subset of spectrum peaks, due to the high combinatorial complexity of pattern mining. While the selection of peaks (or m/z differences) is also used in several *in silico* structural elucidation approaches [58, 40, 41], this may result in a loss of chemically relevant information. The current criterion is based solely on peak intensities, but alternative methods including molecular formulas may be of interest to extract chemically enriched patterns.

Importantly, the representation of spectra as graphs and the identification of common subgraphs offer new perspectives for structural elucidation. On the one hand, representation as a DAG is complementary to fragmentation trees [58]. Indeed, it does not require knowledge of the molecular formula (although this option could be added in future versions of the package to enrich and validate the molecular formulas of m/z differences). Furthermore, it makes no assumptions about the particular fragmentation tree within the graph, and only annotates m/z differences whose molecular formula presents no ambiguity to the accuracy of the mass spectrometer. Like the fragmentation tree, the DAG representation does not rely on databases. It could therefore prove useful for learning algorithms for compound structure prediction. Finally, identification of the biggest common AFG between pairs of spectra may be a valuable similarity metric compared to the classical cosine score.

## Additional file

Supplementary file 1 (Notes about the mineMS2 algorithms, parameters used with mineMS2, MS2LDA, and GNPS, patterns explaining the GNPS similarities described in [50, 51], and supplementary figures)

Supplementary file 2 (Description of the ChemOnt concepts of the LIMS-DB dataset better explained by mineMS2 than MS2LDA.)

Supplementary file 3 (Description of the ChemOnt concepts of the LIMS-DB dataset better explained by MS2LDA than mineMS2.)

## Data Availability

The Penicillium-DB dataset is included in the mineMS2 package, which is freely available at https://github.com/odisce/mineMS2. The LIMS-DB dataset requires a license and cannot be shared publicly. A detailed tutorial about the use of mineMS2 to mine spectral libraries and interpret the patterns, as used in the ’Results and discussion’ section, is available in the two package vignettes.
