# Supplementary material for: mineMS2: annotation of spectral libraries with exact fragmentation patterns
Source: J Cheminform. 2025 Jul 24;17:111. doi: 10.1186/s13321-025-01051-y (PMC12291297; doi:10.1186/s13321-025-01051-y)
Supplement: Supplementary file 1 — (Notes about the mineMS2 algorithms, parameters used with mineMS2, MS2LDA, and GNPS, patterns explaining the GNPS similarities described in [50, 51], and supplementary figures) [file 13321_2025_1051_MOESM1_ESM.pdf]

Supplementary material

mineMS2: Annotation of spectral libraries with exact fragmentation patterns

Alexis Delabrière[1,*], Coline Gianfrotta[2,3*], Sylvain Dechaumet[2,3], Annelaure Damont[2,3], Thaïs Hautbergue[4], Pierrick Roger[6], Emilien L. Jamin[4,5], Olivier Puel[4], Christophe Junot[2,3], François Fenaille[2,3] and Etienne A. Thévenot[2,3]

[1]Université Paris-Saclay, CEA, List, Palaiseau, France, [2]Département Médicaments et Technologies pour la Santé, Université Paris-Saclay, CEA, INRAE, MetaboHUB, Gif-sur-Yvette, France, [3]MetaboHUB-IDF, National Infrastructure of Metabolomics and Fluxomics, Gif-sur-Yvette, France, [4]Toxalim (Research Centre in Food Toxicology), INRAE UMR 1331, ENVT, INP-Purpan, Toulouse University, Toulouse, France, [5]MetaboHUB-Metatoul, National Infrastructure of Metabolomics and Fluxomics, Metatoul-AXIOM, Toulouse, France, [6]Centre National de Recherche en Génomique Humaine, CEA Paris-Saclay, Institut François Jacob, Evry, France

[*]Contributed equally to this work

Contacts: alexis.delabriere@hotmail.fr, coline.gianfrotta@ens.uvsq.fr, and etienne.thevenot@cea.fr

Contents

Note S1: Details about the mineMS2 algorithms. 3

Note S2: Parameter values used with the mineMS2, MS2LDA and GNPS software. 7

Note S3: Selection of the mineMS2 parameter values. 8

Note S4: Description of the patterns obtained with the Penicillium-DB dataset, explaining the fragmentation similarities described by the authors in Hautbergue et al. [2017, 2019]. 9

Figure S1: mineMS2 execution time as a function of the number of spectra. 35

Figure S2: Number of ChemOnt concepts explained by mineMS2 and MS2LDA at a given precision or recall level. 36

Figure S3: Explainability of ChemOnt concepts by mineMS2 patterns obtained with single or multi-energy (merged) spectral collections. 37

Figure S4: Best mineMS2 patterns explaining the ChemOnt concepts *Flavonols* and *Delta-5-steroids* obtained with the single or multi-energy spectra collections. 38

Figure S5: Example of the *Flavans* concept, composed of 4 metabolites, and its best explaining mineMS2 pattern. 46

Figure S6: Correspondence between mineMS2 patterns and SIRIUS fragmentation trees for two ChemOnt concepts. 48

Figure S7: Proposed structures of unknown secondary metabolites detected at m/z 416, 391 and 393 in the *Penicillium* studies [Hautbergue et al., 2017, 2019]. 49

Additional files **49**

Table S1: Description of ChemOnt concepts in the LIMS-DB dataset, better-explained by mineMS2 than by MS2LDA (higher F1-score) and with an F1-score greater than 0.7 (lims-db__patterns__minems2__better.xlsx) **50**

Table S2: Description of ChemOnt concepts in the LIMS-DB dataset, better-explained by mineMS2 than by MS2LDA (higher F1-score) and with an F1-score greater than 0.7 (lims-db__patterns__ms2lda__better.xlsx) **50**

Note S1: Details about the mineMS2 algorithms.

DAG creation

Each MS/MS spectrum is first converted to a DAG (Directed Acyclic Graph), with each vertex representing a fragment ion and each edge corresponding to a m/z difference. To enable subsequent comparison between individual DAGs, the m/z differences are binned.

The DAG creation step takes as input the complete set of MS/MS spectra $\mathcal{D}$ and returns a set of DAGs (one for each spectrum), with a set of common edge labels $\mathcal{L}(\mathcal{D})$. These labels are the binned m/z differences.

Binning of m/z differences

The method to compute the binning of m/z differences is described in the following paragraph.

A spectrum S is considered as a set of n pairs, $(mz_1, int_1), \ldots, (mz_n, int_n)$ where mz_j and int_j denote the mass-to-charge ratio and intensity from the j-th peak of the spectrum. The maximum value of $\boldsymbol{n}$ is a parameter fixed by the user. If there are more than n peaks in the spectra, only the n most intense are kept (n is set to 15 by default and can be increased up to 20). To simplify the notation, we define the following vectors $\boldsymbol{mz} = mz_1, ..., mz_n$ and $\boldsymbol{int} = int_1, ..., int_n$. We assume that the spectrum is stored in increasing order of m/z. First, all the possible m/z differences within each spectrum S are computed: $\boldsymbol{d_S} = mz_i - mz_j, \forall i < n, j < n, i > j$. The m/z differences from all spectra are concatenated into a single $\boldsymbol{diff_{mz}}$ vector, which is then discretized using a gaussian kernel density estimation (Algorithm 1, line 1).

Every bin with labels appearing with a frequency inferior to a threshold **e** is discarded (Algorithm 1, line 2).

The overlap between every consecutive pairs of bins is calculated and if the overlap is superior to a limit α, the two bins are fused, and the mean *mu* and standard deviation *sigma* of the fused bin are calculated (Algorithm 1, line 3 to 13).

Formula generation

The function *generateFormula* (Algorithm 1, line 14) generates all elemental compositions using the algorithm described in [Böcker and Liptak, 2007], then filters them for the existence of a planar graph [Kind and Fiehn, 2007].

The output of the Algorithm 1 is a set of disjoint m/z bins $\mathcal{L}(D)$ with at least one formula except for the m/z values superior to 200.

Converting each spectrum into a DAG

Then, for each spectrum $70S$, a directed graph $G_S = (V_S, E_S)$ is created by starting from the precursor ion and linking the up to n most intense fragment ions (vertices) with m/z differences from $\mathcal{L}(D)$ (edges). The resulting graph is acyclic (DAG) since there is no edge from i to j when $mz_i < mz_j$.

Checking edge labeling

Finally, a step to check the edge labeling is performed. As described in the properties of the fragmentation graphs, if, in any DAG $G_S \in D$, there is a triplet of vertices u, v, w, such that $(u, v) \in E_S$ and $(v, w) \in E_S$ with respective labels $a = l(u, v) \in \mathcal{L}(D)$ and $b = l(v, w) \in \mathcal{L}(D)$, then $(u, w) \in E_S$ and it exists a unique c such that $l(u, w) = a+b = c \in \mathcal{L}(D)$. Due to the binning, different values of c can be found for the same pair (a, b). To address this, if different values of c are found among the DAGs of the dataset, the corresponding bins, which are necessarily close, are merged.

To provide further empirical evidence supporting Property 1 and 2, we looked for occurrences of "triangle labeling error": if a triangle with edge labels E, F, G was found in a dataset, we counted in each DAG the number of instances where two edges were correctly labeled, but the third edge was missing or was not correctly labeled. In the LIMS-DB dataset, such a labeling error was detected in less than 0.7% of the triangles, covering 8% of the individual DAGs. No labeling error was detected in the Penicillium-DB datasets. These results confirm that labeling errors are rare.

Demonstration of the theorem of spanning trees

Let G and H be two induced connected subgraphs from our set of graphs $\mathcal{D}$.

In this demonstration, we will denote the fact that the graph G is isomorphic to the graph H by $G \simeq_e H$.

Algorithm 1 Binning of m/z differences

Input: $diff_{mz}$ list of m/z differences from all spectra in the dataset $\mathcal{D}$
ppm m/z tolerance in ppm
dmz minimum deviation for low m/z in Da
f frequency threshold
α overlap limit between two bins above which they will be merged

Output:
A list of frequent disjoint bins with formula

$bins \leftarrow normDensityClust(diff_{mz}, ppm, dmz)$ ▷ bins consist of pairs mu, $sigma$
Remove from bins the labels which are not present at least f times
$k \leftarrow 0$
while $k < number\ of\ bins$ **do** ▷ Overlapping bins are fused
  if $Overlap(bins_k, bins_{k+1}) > \alpha$ **then**
    $mu_f \leftarrow \frac{mu_k - 3 \times sig_k + mu_{k+1} + 3 \times sig_{k+1}}{2}$
    $sig_f \leftarrow mu_{k+1} + 3 \times sig_{k+1} - mu_k + 3 \times sig_k$
    $fusedBin \leftarrow (mu_f, sig_f)$
    Replace $bins_k$ and $bins_{k+1}$ by $fusedBin$
  else
    $k = k + 1$
  end if
end while
$F \leftarrow generateFormula()$
match F to each $bins_k$
return bins

We want here to show that G and H are isomorphic i.f.f there exists one spanning tree from G, T_G, and one from H, T_H, such that T_G is isomorphic to T_H.

Let us prove that $(G \simeq_e H) \Rightarrow \exists T_G, T_H$ such that $(T_G \simeq_e T_H)$

If G and H are isomorphic, then any spanning tree of G is isomorphic to any spanning tree of H, by considering the same isomorphism as between G and H. So, $(G \simeq_e H) \Rightarrow (T_G \simeq_e T_H)\ \forall T_G, T_H,$ spanning trees of G and H, respectively.

Let us prove that if it exists T_G, T_H such that $(T_G \simeq_e T_H)$, then $(G \simeq_e H)$

Let us now consider one spanning tree T_G of G and one spanning tree T_H of H such that T_G and T_H are isomorphic.

Let us call f the isomorphism from $V(T_G)$ to $V(T_H)$. We show hereafter that there is an equality between the edges and vertices sets of G and their image by f in H.

Vertex set equality

By definition of the spanning arborescence, it is clear that $V(H) = \{f(v), v \in V(G)\}$, as a spanning tree of a graph contains all the vertices of the graph.

Edge set equality

The edge set equality may be proved by contradiction.

Let us consider an edge $e = (u, v)$ such that $(u, v) \in E(G)$ but $(f(u), f(v)) \notin E(H)$. Let us denote $T_G + e$ the graph obtained by adding the edge e to T_G. Because $T_G \simeq_e T_H$ and T_H is a subgraph of H, by transitivity of the subgraph relationship, T_G is a subgraph of H. Moreover, $T_G + e$ is not a subgraph of H.

As T_G is a subgraph of $T_G + e$, we have that $Supp_D(T_G) \geq Supp_D(T_G + e)$. But, as T_G is a subgraph of both G and H and $T_G + e$ is a subgraph of G but not of H, we have $Supp_D(T_G) > Supp_D(T_G + e)$. However, based on Property 2, we have $Supp_D(T_G) = Supp_D(T_G + e)$ since $T_G + e$ is equal to T_G plus an edge e. Therefore, we have a contradiction and e cannot exist. We thus have: $E(G) \subseteq E(H)$.

Conversely, we show that $E(H) \subseteq E(G)$, and thus $E(G) = E(H)$.

We have therefore demonstrated that if it exists T_G,T_H such that ($T_G \simeq_e T_H$), then ($G \simeq_e H$).

We thus proved the theorem.

k-path tree generation

To build the k-path tree (Algorithm 2), the fragmentation graphs from the database $\mathcal{D}$ are processed sequentially (line 2). For each graph, the enumeration of all paths of size k is performed using a Depth-First Search (DFS) approach exploring all edges (line 6). Each time a path is found, it is inserted into the k-path tree (line 34). Since our fragmentation graphs are limited to at most 20 vertices, the enumeration of all the paths remains computationally tractable. Moreover one procedure further limits the computation and memory costs: line 33 ensures that the path is potentially frequent (Property 2). It should be noted that this condition is necessary but not sufficient. Once the k-path tree is built, the remaining non frequent paths are removed by exploring all vertices and checking their frequencies. The generated k-path tree contains all the frequent paths of size k or less.

Algorithm 2 Building of the k-path tree

```
1: function constructKPathTree(𝒟,k)
2:     for i ∈ 1, . . . , |𝒟| do
3:         AddToKPathTree(T,𝒟[i],k,i)                        ▷ T is the k-path tree
4:     end for
5: end function
6: procedure AddToKPathTree(T,G,k,id)
7:     N ← empty stack
8:     R ← vertices from G without parents                   ▷ roots in G
9:     S ← empty list                                        ▷ to store the current path
10:    initialize all edges in G to not visited   ▷ edges can be not visited, currently visited, or visited
11:    for r ∈ R do
12:        n ← r, s ← NULL
13:        push n into N
14:        S ← [n]
15:        s ← next reachable vertex from n by a not currently visited edge if exists, else NULL
16:        put the edge (n, s) as currently visited if exists
17:        while n ≠ r or s ≠ NULL do
18:            if s = NULL then
19:                n ← pop an element of N
20:                label all outgoing edges from n as visited
21:                remove the last element of S
22:                if !isEmpty(N) then
23:                    n ← pop an element of N
24:                    push n into N
25:                else
26:                    s ← NULL
27:                    continue
28:                end if
29:            else
30:                push s into N
31:                append s to S
32:                for i in min(k, |S|), . . . , 1 do
33:                    if |S| − i > 0 and there is an edge from S[|S| − i] to s then
34:                        insert the path from S[|S| − i] to s into the k-path tree T
35:                    end if
36:                end for
37:                n ← s
38:            end if
39:            s ← next reachable vertex from n by a not currently visited edge if exists, else NULL
40:            put the edge (n, s) as currently visited if exists
41:        end while
42:    end for
43: end procedure
```

Criteria to mine Acyclic Flow Graphs (AFGs) using the k-path tree

To enumerate frequent AFGs, mineMS2 ensures that three criteria are met by each subtree from the k-path tree: the candidate subtree is checked to be a proper subtree of a graph of $\mathcal{D}$, to be in k-LMDF form, and to be frequent.

Ensuring that a candidate is a proper subtree

By enumerating all subtrees from the k-path tree, it is possible to enumerate subtrees which never occur in the fragmentation graphs, or which are not subtrees but subgraphs, even if all their paths are frequent.

Consider the subtree of Fig. 3 containing the 2 left-most branched of the tree (Root$\rightarrow H_2O \rightarrow CO_2$ and Root$\rightarrow CO_2 \rightarrow H_2O$ joined by the root). While this subtree is a subgraph of the 2-path tree, it does not occur in the fragmentation graph 1. This happens when two paths of the k-path tree lead to vertices with the same h value. Therefore during the extension process, at each step, the h values of the current subtree from the k-path tree are stored. When this subtree is expanded, all the h values of the possible extensions are checked. If an h value already exists in the set of h values of the subtree, the corresponding extension is removed from the set of extensions, avoiding to lead to impossible subtrees.

Ensuring that a candidate is in k-LMDF form

To ensure that each candidate is in k-LMDF form, we use the DFS (Depth-First Search) code [Yan and Han, 2002] as a label for each edge extension. After each extension of an edge e, all extensions with a lower DFS code than e are removed.

Calculating the support of a candidate and ensuring that it is frequent

The set of occurrences of any path in the k-path tree is stored in the last vertex of the path.

We can use it to calculate the support of a subtree, because the support of a proper subtree of the k-path tree in the database $\mathcal{D}$ is the intersection of the occurrences sets of every path constituting this subtree.

If the size of this intersection is lower than the user-defined threshold, the pattern is considered as not frequent and then eventually discarded.

Completeness of the mineMS2 subgraph mining algorithm

We wish to demonstrate here that our tree extension procedure does not miss any frequent AFG.

The completeness of the right-most extension has been proven in [Zaki, 2002]. However, as described previously, the mineMS2 FSM algorithm stops this process of extension if at least one of the three criteria is not verified (i.e. not a proper subtree, or not in the k-LMDF form, or not frequent). Nevertheless, if a tree does not meet one criterion, none of his supertrees does (i.e. the negation of each criterion is transitive by the subgraph relationship). The discarded part of the mining space therefore only includes trees which do not correspond to frequent AFGs.

Note S2: Parameter values used with the mineMS2, MS2LDA and GNPS software.

The parameter values used to process the LIMS-DB and Penicillium-DB datasets with mineMS2 (respectively, MS2LDA) are indicated in Table 1 (respectively, Table 2) below. The parameter values used to process Penicillium-DB data with GNPS [Wang et al., 2016] are indicated in Table 3. The values which are different from the default ones are shown in bold.

Table 1: mineMS2 parameters

	LIMS-DB and Penicillium-DB
ppm	15
dmz	0.007
maxFrags	15
atoms	C:16, H:50, O:15, N:10, Cl:2, S:2, P:2
limMzFormula	14.5, 200
maxOverlap	0.05
count	2
sizeMin	1
threshold	0.2

Table 2: MS2LDA parameters

	LIMS-DB	Penicillium-DB
isolation_window	**0.5**	
bin_width	0.005	
min_ms1_rt	180	
max_ms1_rt	1260	
min_ms2_intensity	**5000**	
filter duplicates	False	
min_ms1_intensity	0.0	
duplicate_filter_mz_tol	0.5	
duplicate_filter_rt_tol	16.0	
n_its	1000	
K	**1000**	**300**

Table 3: GNPS parameters for Penicillium-DB (both *P.verrucosum* and *P.nordicum* datasets)

**Penicillium-DB**	
Precursor ion mass tolerance	0.2 Da
Fragment ion mass tolerance	0.02 Da
Minimum pairs cosine score	0.6
Network topK	100
Maximum connected component size	100
Minimum matched fragment ions	4
run MSCluster	OFF
Minimum cluster size	1
Filter precursor window	No
Filter peaks in 50Da window	Yes

Note S3: Selection of the mineMS2 parameter values.

The default parameter values gave the best results on the two tested datasets (LIMS-DB and Penicillium-DB), in terms of relevance with the ChemOnt concepts and the GNPS network, respectively, and can thus be used as a good starting point. More specifically, the values of the most important parameters are discussed below:

ppm Selecting a lower *ppm* tolerance tends to decrease the size of the patterns and their support, and thus, of the information they provide. For example, for the LIMS-DB dataset, when the tolerance was set to 7 ppm, the average F1-score for the ChemOnt concepts was decreased to 0.47 (instead of 0.53 with the default tolerance of 15 ppm). In addition, the patterns best explaining the ChemOnt concepts at 7 ppm contained 5.2 m/z differences on average, versus 6.8 m/z differences with the 15 ppm tolerance. It can be noted that the tolerance in *ppm* is used to define the bandwidth of the kernel when aligning the m/z differences between the spectra. It also defines the bin width, that cannot be chosen independently by the user.

count Regarding the minimum size of the support (minimum number of spectra in which a pattern should be detected to be considered), the default value (2) enables very specific patterns to be extracted.

minSize The minimum size of the patterns is by default set to 1 in order to detect (frequent) single m/z differences. Note that this default value is automatically increased to 2 (generating a warning) when the number of frequent m/z differences is large ($>$ 600) in the spectral collection.

maxFrags The default maximum number of fragments is set to 15 as a compromise to extract most of the spectral information while avoiding computational overload. As an example, the mining of the LIMS-DB dataset (622 spectra) with a *maxFrags* value of 20 (instead of 15) increased the running time to 1.5 h (instead of 20 min) and resulted in a slight increase of concepts explained with F1-scores above 0.7, as shown in the figure below.

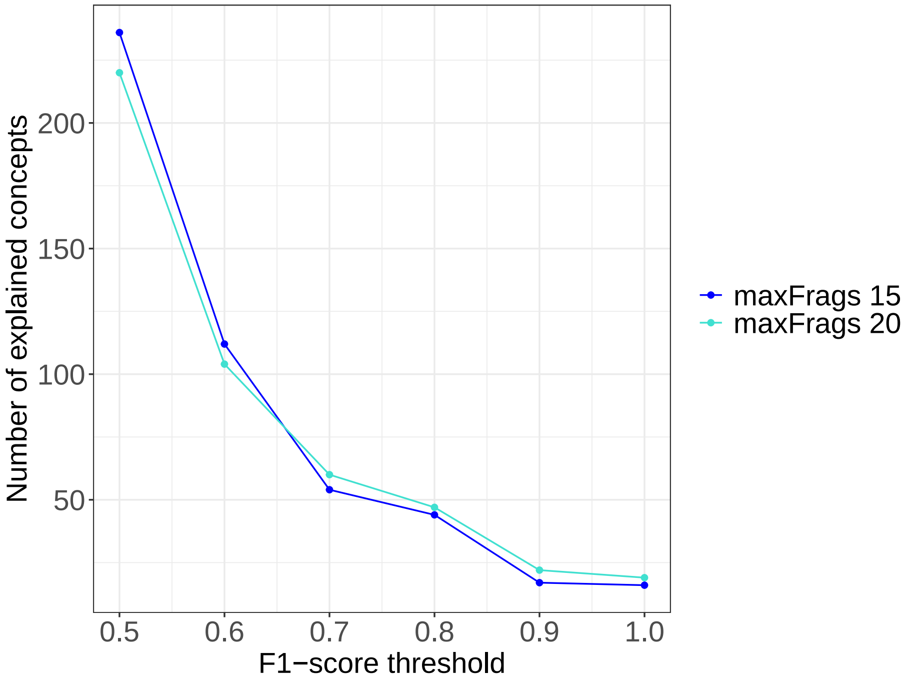

Note S4: Description of the patterns obtained with the Penicillium-DB dataset, explaining the fragmentation similarities described by the authors in Hautbergue et al. [2017, 2019].

The specific mineMS2 fragmentation patterns (i.e. with the best F1-score) for 8 clusters of MS/MS spectra reported in Hautbergue et al. [2017, 2019] are described below (4 clusters in each study). For each pattern:

- all MS/MS spectra containing the mineMS2 pattern are shown, and the detected similarity is highlighted in color
- the mineMS2 pattern is displayed; for m/z < 200, edges are labelled with the molecular formula (in case of several possible formulas, the one with the closest m/z is shown in parentheses); for m/z > 200, edges are labelled with the m/z values
- the MS2LDA motif with the highest F1-score is indicated (if it exists)

Dataset *Penicillium verrucosum* (Hautbergue *et al.*, 2017)

Ochratoxins A and B

Ochratoxin A (m/z 404.0897 and rt 33.1; the major mycotoxin of *P. verrucosum*) and ochratoxin B (m/z 370.13 and rt 26.5) are very similar in structure and share a large fragmentation pattern. The pattern notably includes a CH_2O_2 loss (corresponding to a loss of H_2O and CO) as well as a loss of NH_3. Another mass difference of 147.0683 is detected and may correspond to a loss of a phenylalanine residue, which is part of ochratoxins. According to the fragmentation graph, this loss may occur after a loss of water, or directly, probably through a rearrangement of the molecule. The formula C_9H_9ON is among the suggested formulas for this mass difference. The edges corresponding to these 3 losses are colored in green in the fragmentation graph. The exact same pattern is found in *P.nordicum*.

Surprisingly, no MS2LDA pattern is detected for the ochratoxins. Each ochratoxin is explained by one pattern, present only in the spectrum of the molecule, but no common pattern is found.

Spectra

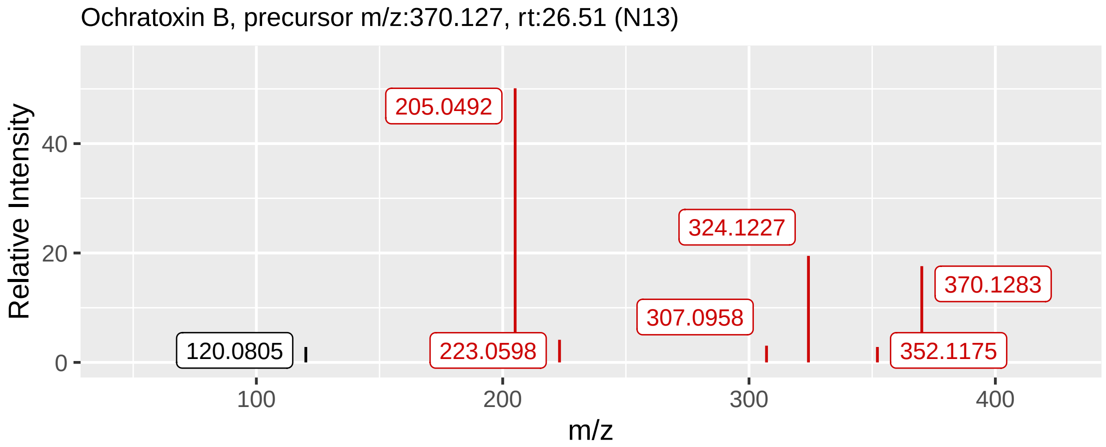

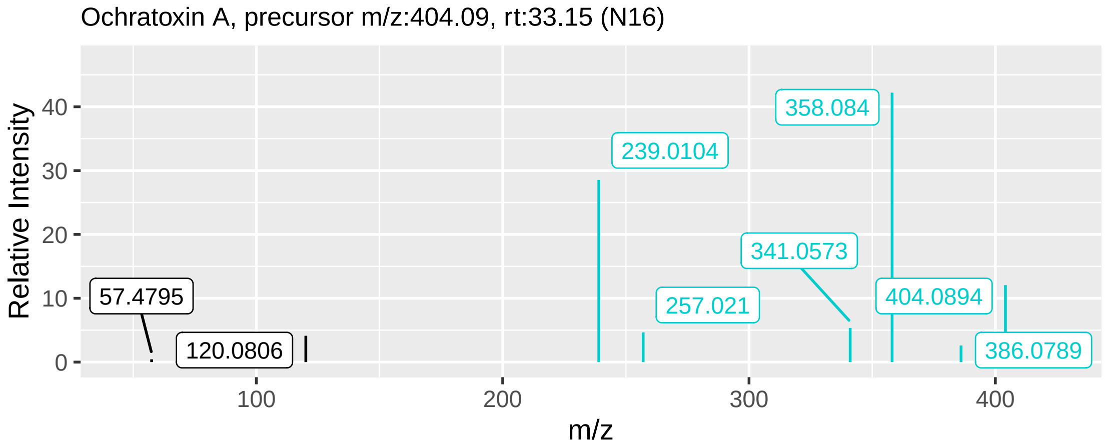

Fragmentation pattern

P33

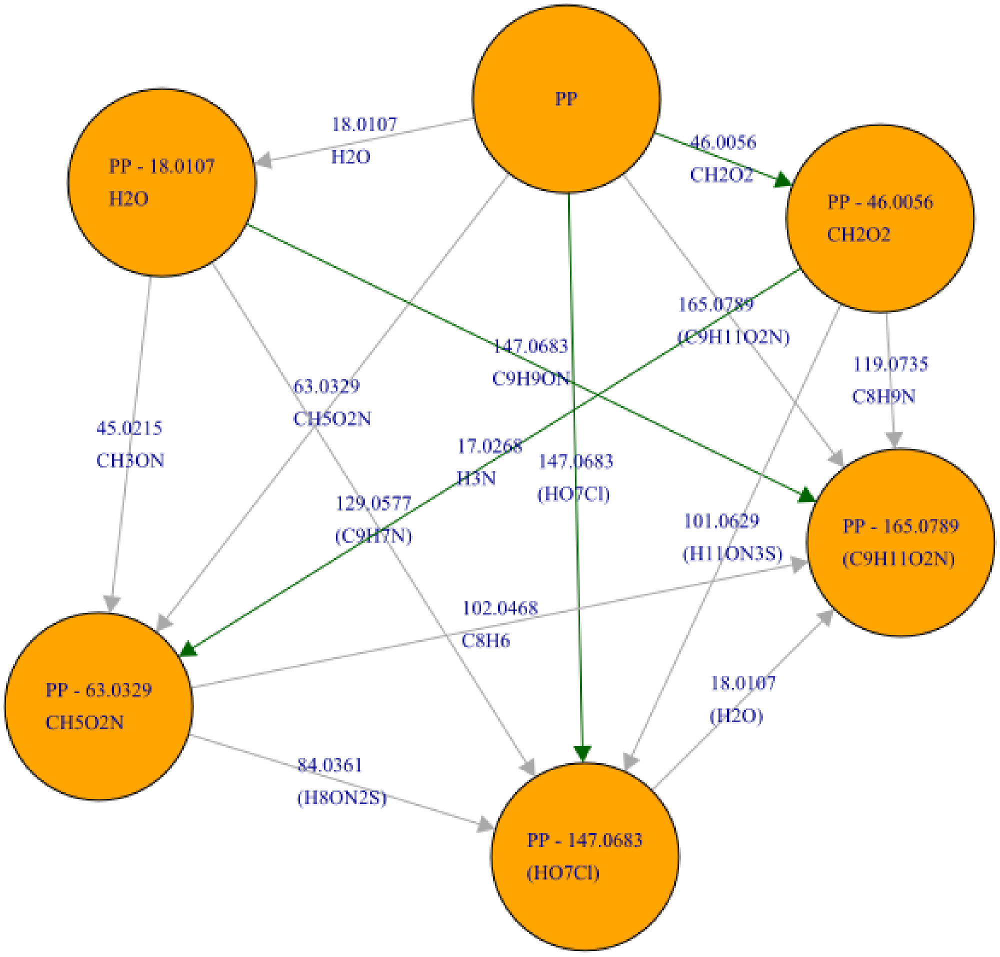

No MS2LDA pattern found

Quinazolines (and in particular verrucines)

The anacine (m/z 343.174 rt 12.7), aurantinomide C (m/z 341.161 rt 20.1), verrucines A/B (m/z 377.16 rt 12.78/18.4) and F (m/z 375.14 rt 18.7) are all linked closely in the GNPS network from the original publication. In particular, as stated in this original publication, verrucine A/B and verrucine F share a common loss of NH_3 and CO. MineMS2 finds a fragmentation pattern containing this mass difference (CH_3ON in the graph), and this pattern is present in the MS/MS spectra of the four cited metabolites, along with their isomers and 4 unidentified other metabolites. This is not surprising, as this loss is indicative of amino acids.

The MS2LDA best pattern is restricted to the loss of NH_3 (dmz of 17.0275), and is present in 10 unidentified metabolites.

Spectra

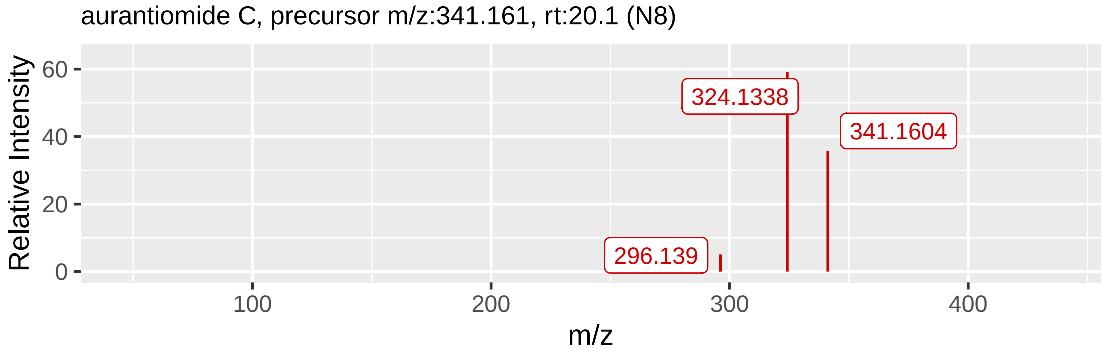

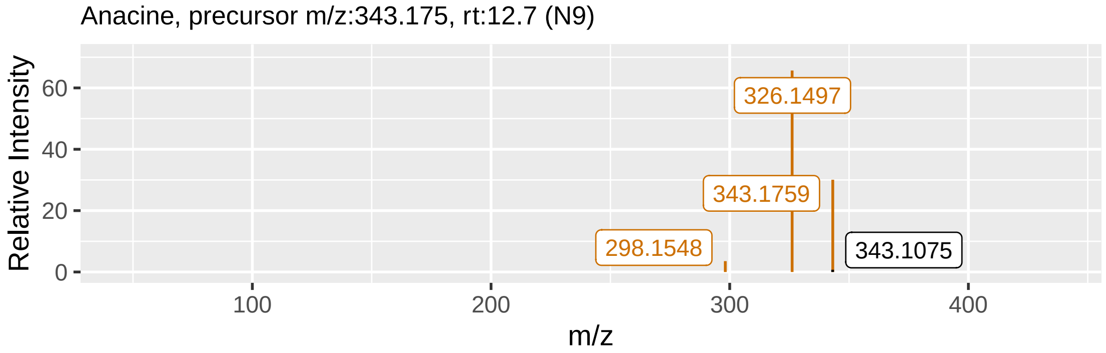

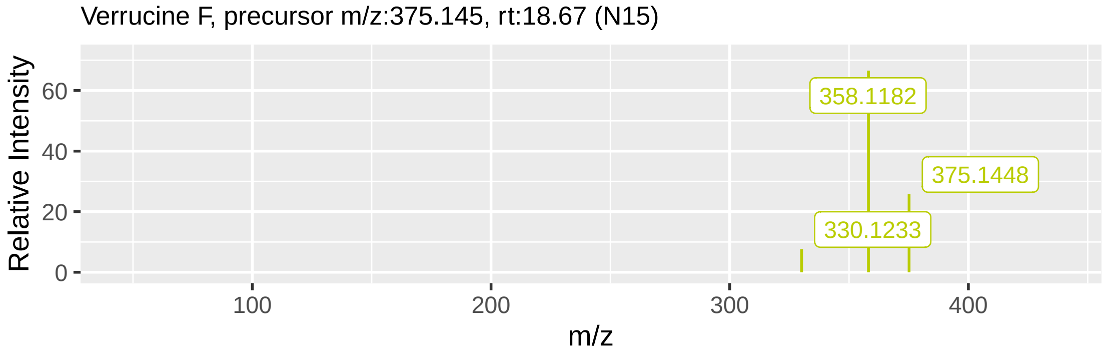

, precursor m/z:341.161, rt:15.06 (N27)

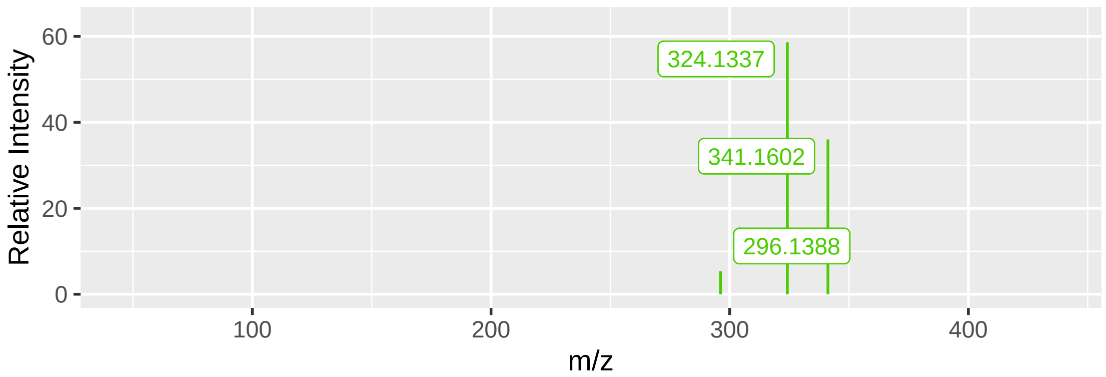

, precursor m/z:343.175, rt:17.9 (N28)

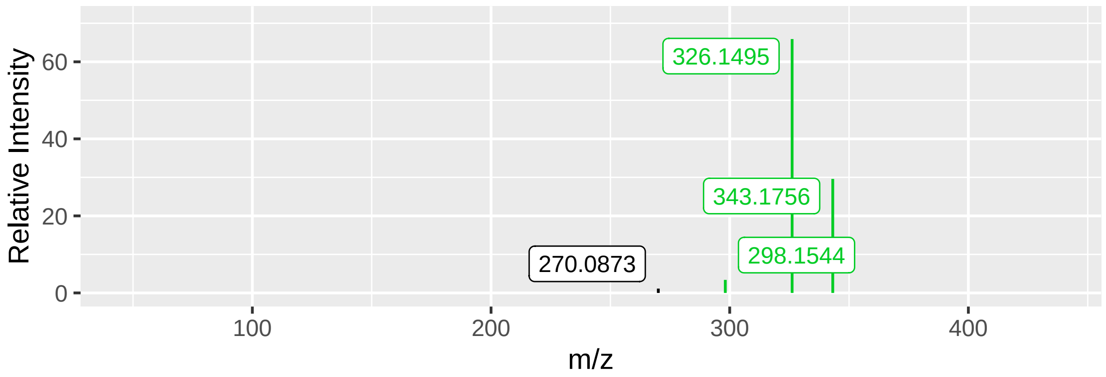

Verrucines A ou B, precursor m/z:377.16, rt:18.4 (N30)

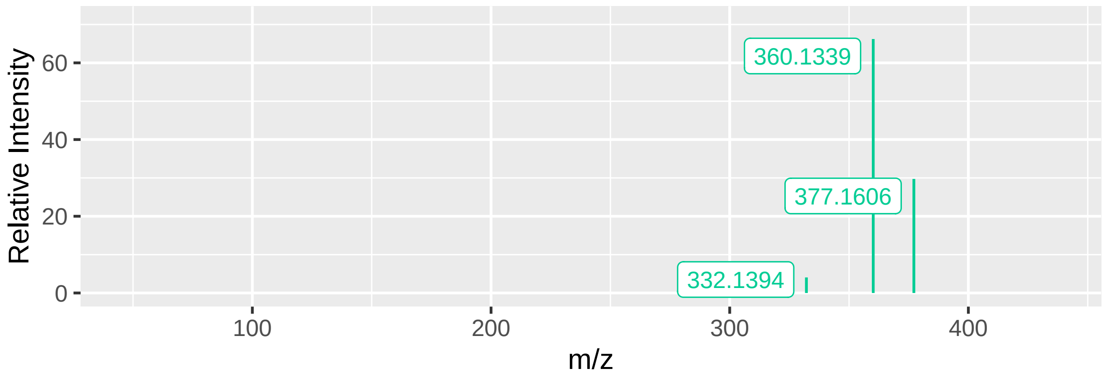

Verrucines A ou B, precursor m/z:377.16, rt:12.78 (N31)

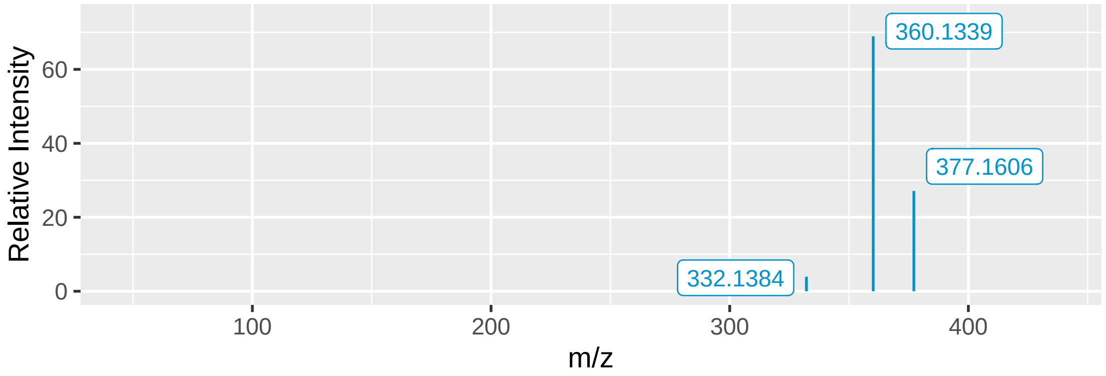

, precursor m/z:391.139, rt:13.46 (N32)

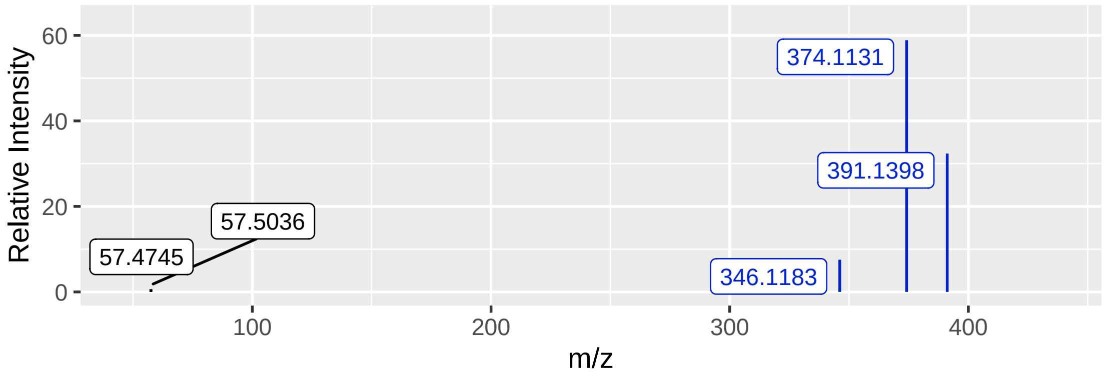

, precursor m/z:391.139, rt:20.98 (N33)

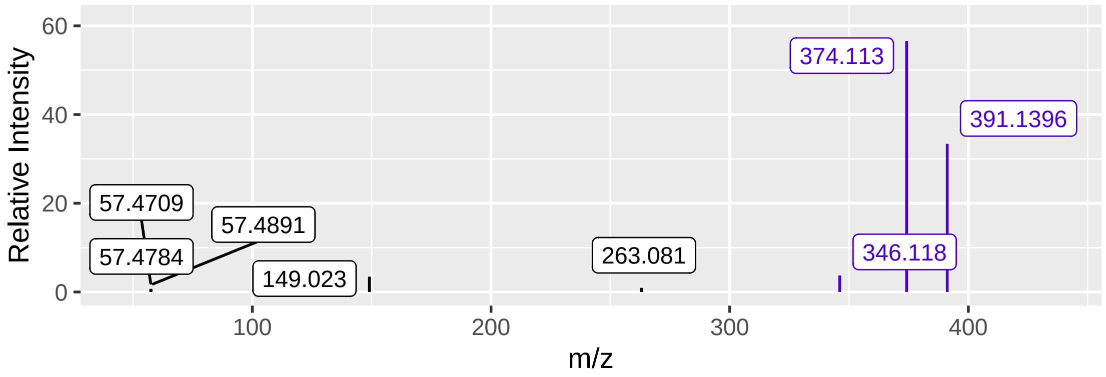

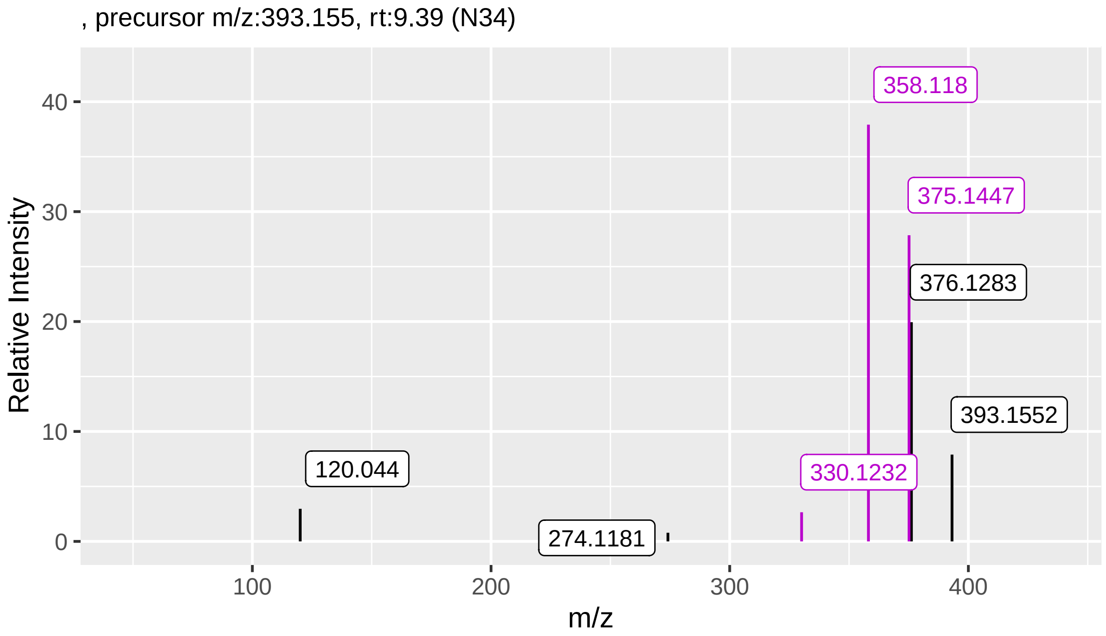
, precursor m/z:393.155, rt:9.39 (N34)
358.118
375.1447
376.1283
393.1552
120.044
330.1232
274.1181
Relative Intensity
m/z
0
10
20
30
40
100
200
300
400

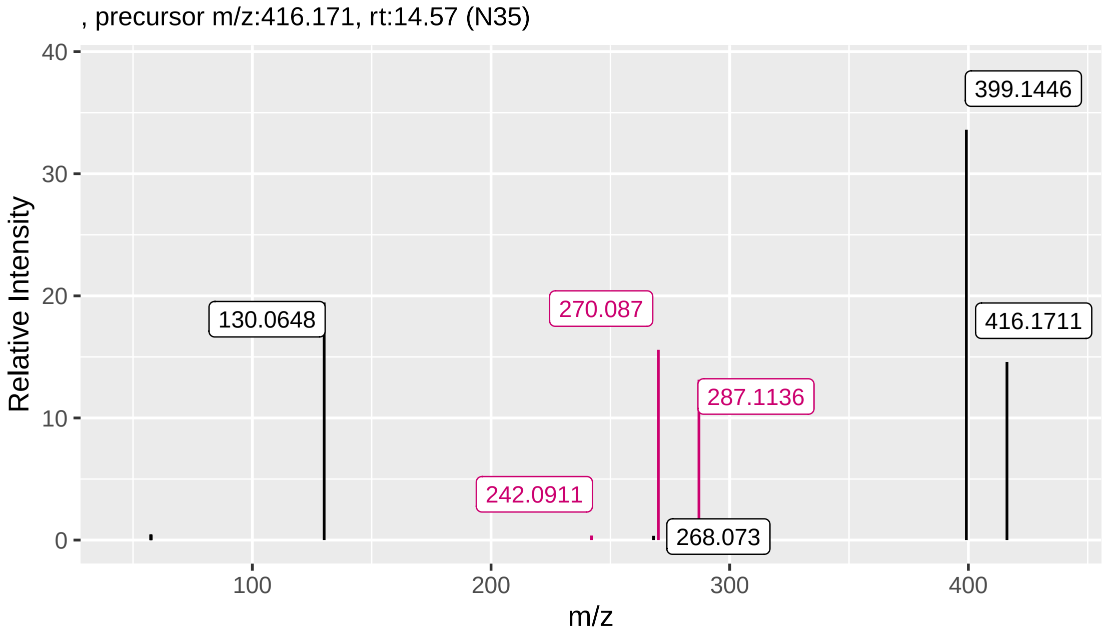
, precursor m/z:416.171, rt:14.57 (N35)
399.1446
130.0648
270.087
416.1711
287.1136
242.0911
268.073
Relative Intensity
m/z
0
10
20
30
40
100
200
300
400

Fragmentation pattern

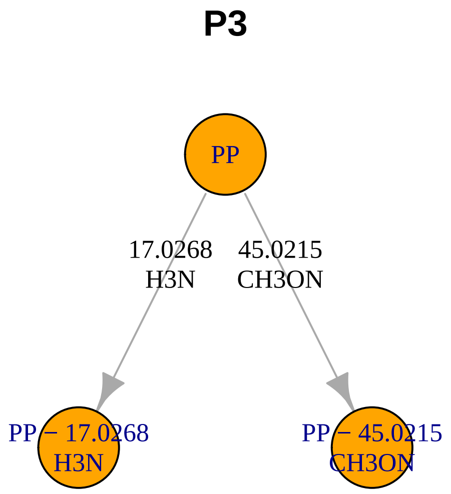

MS2LDA pattern 230

Feature	m/z mean	m/z min	m/z max	Probability
loss	17.0275	17.025	17.03	0.998

Metabolites containing this pattern

Precursor m/z	Retention time	Name	Probability
513.2125	30.09		0.497
513.2125	27.13		0.368
513.2122	26.70		0.333
513.2119	24.60		0.404
513.2119	20.56		0.383
513.2119	26.31		0.274
416.1712	14.57		0.203
393.1554	9.39		0.102
391.1392	20.98		0.357
391.1392	13.46		0.354
377.1604	12.78	Verrucine A/B	0.397
377.1604	18.40	Verrucine A/B	0.386
375.1450	18.67	Verrucine F	0.380
343.1747	17.90	Anacine	0.387
341.1608	20.10	Aurantiomide C	0.358
341.1608	15.06		0.355

Verrucolone and $C_6H_8O_3$

Verrucolone (m/z 147.06 rt 3.1; also known as arabenoic acid) and the metabolite with the formula $C_6H_8O_3$ (m/z 129.05 rt 7.6) share a common structure. The latter was suspected to be a dehydroxylated form of verrucolone (Hautbergue et al., 2017). A specific fragmentation pattern is found by mineMS2 exclusively in verrucolone and $C_6H_8O_3$. This pattern contains five ions (see the spectra below), including the three mentioned in the publication: 97.0280, 87.0437 and 129.054.

In contrast, no common MS2LDA pattern is found for these two metabolites.

Spectra

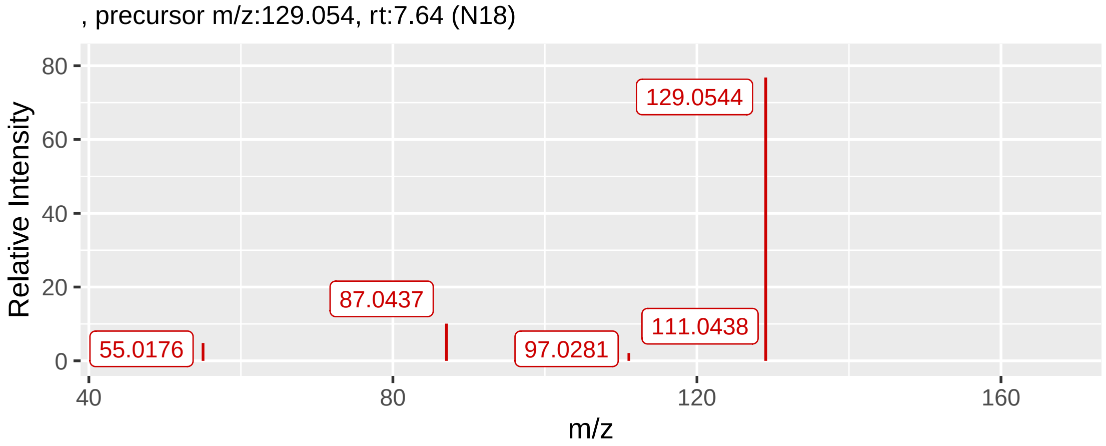

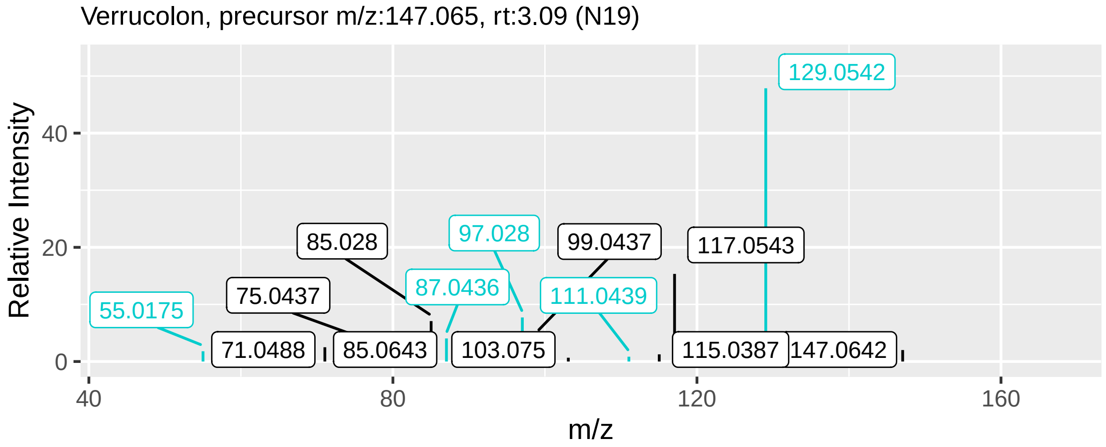

Fragmentation pattern

P22

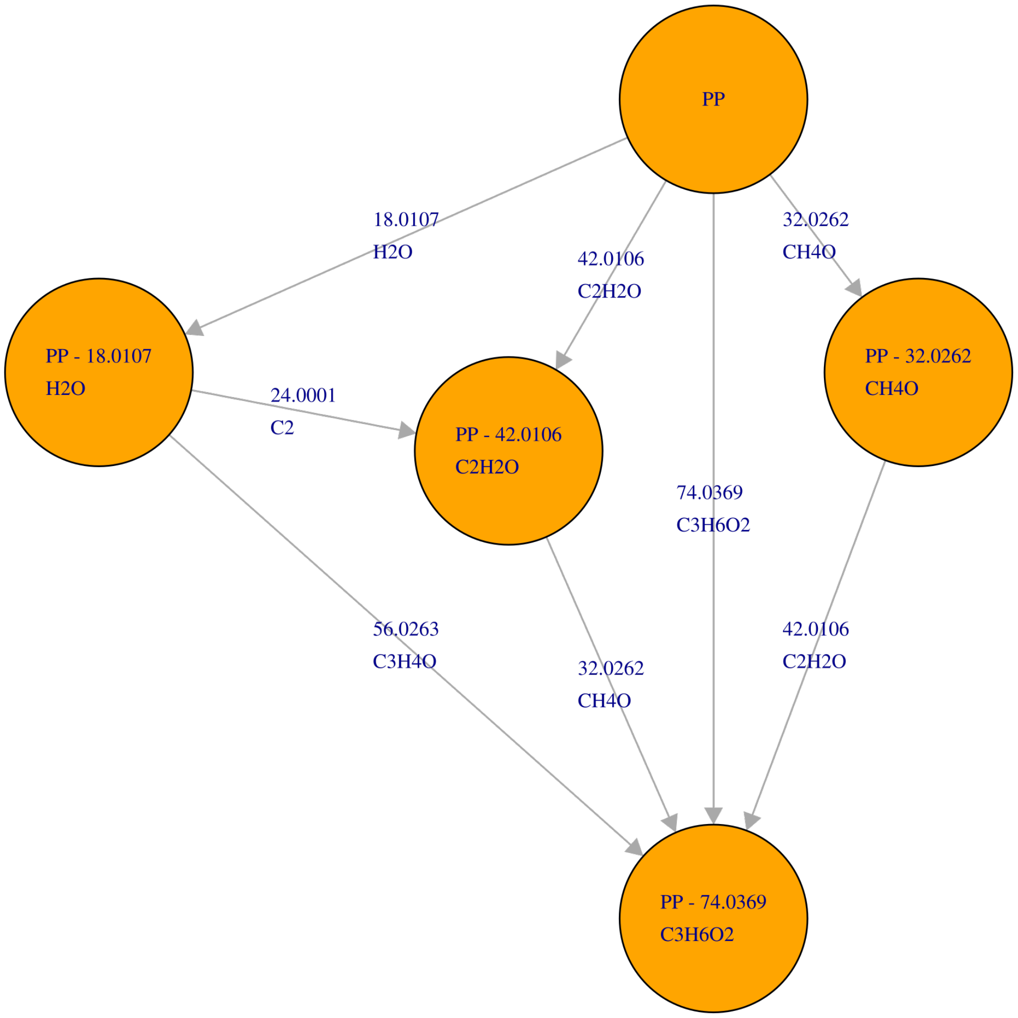

No MS2LDA pattern was found.

Isomers at m/z 511.29

A fragmentation pattern is detected between the two isomeric ions described in the publication (m/z 511.29 rt 17.92 and m/z 511.29 rt 18.58). It includes 6 ions out of the 7 manually observed in the publication, and contains mass differences of 99.0682 and 147.0683 (colored in green in the graph). These mass differences (and assigned molecular formulas) suggest that they correspond respectively to the valine and phenylalanine losses described in the article (Hautbergue et al.; 2017). The missing fragment, of low intensity, was discarded during the pre-processing step.

MS2LDA finds a pattern with the same 6 fragments.

Spectra

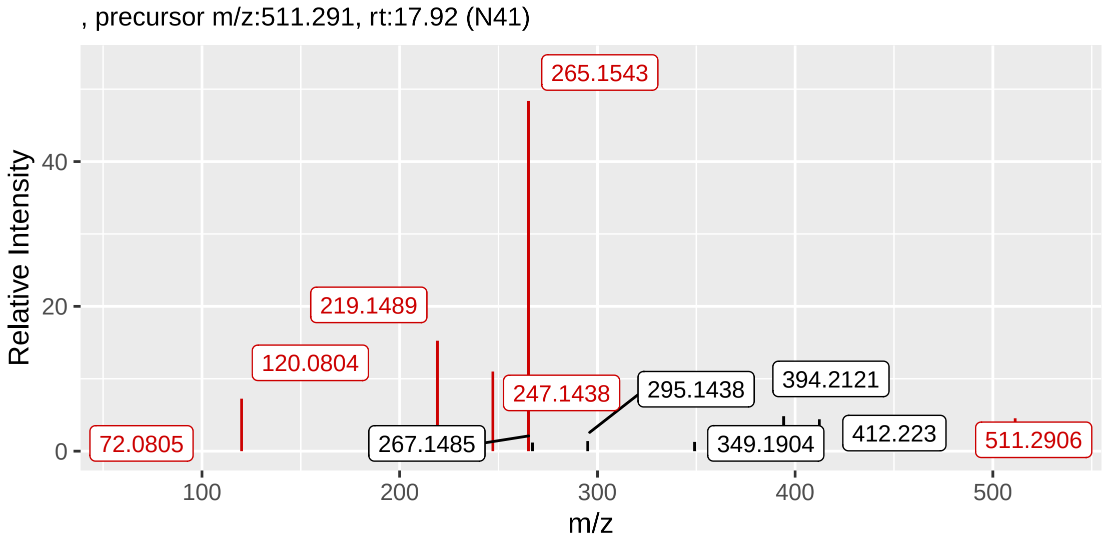

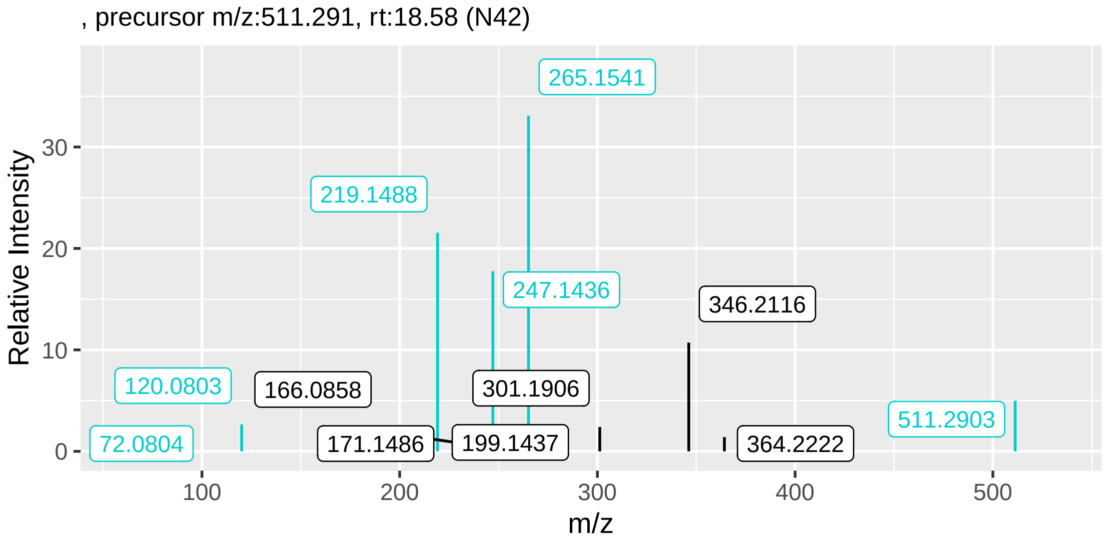

Fragmentation pattern

P90

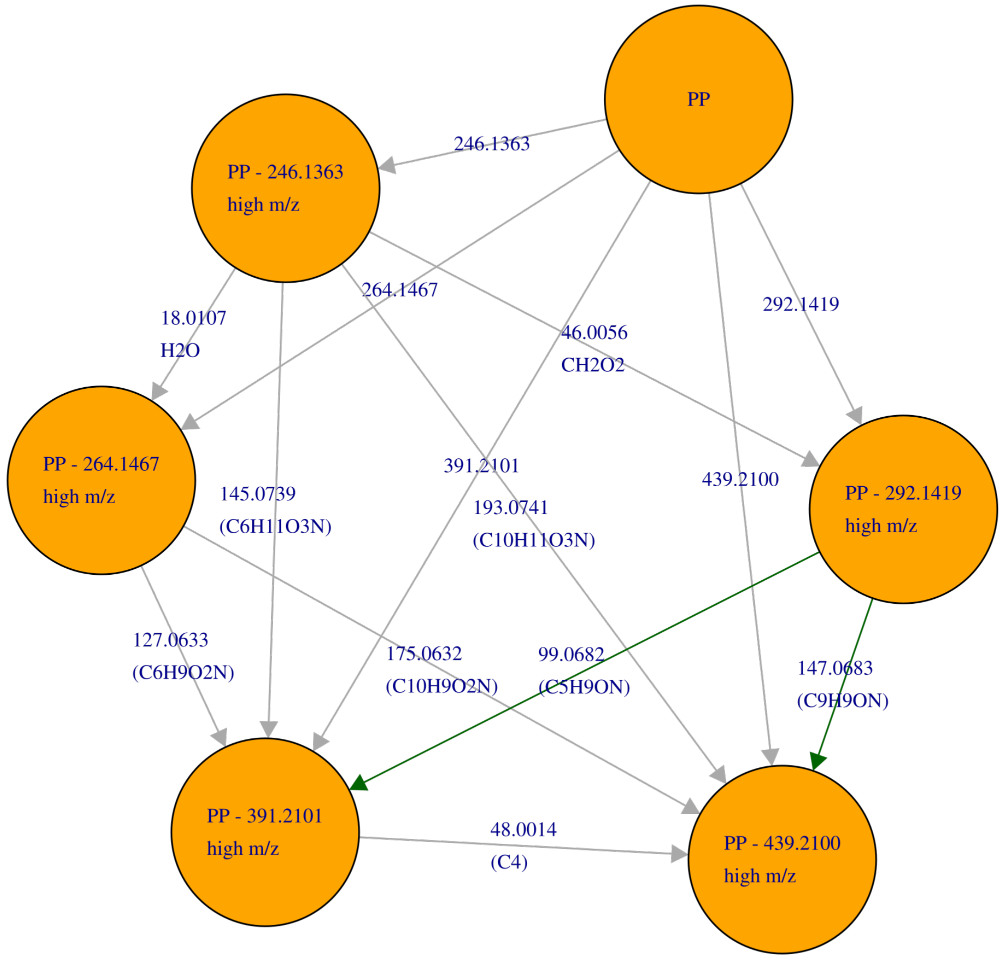

MS2LDA pattern 236

Feature	m/z mean	m/z min	m/z max	Probability
fragment	72.0825	72.080	72.085	0.005
fragment	120.0825	120.080	120.085	0.047
fragment	219.1475	219.145	219.150	0.182
fragment	247.1425	247.140	247.145	0.131
fragment	265.1525	265.150	265.155	0.573
fragment	511.2925	511.290	511.295	0.053

Metabolites containing this pattern

Precursor m/z	Retention time	Name	Probability
511.2909	17.92	NA	0.756
511.2909	18.58	NA	0.509

Dataset *Penicillium nordicum* (Hautbergue *et al.*, 2019)

Aurantiomide C and anacine

Aurantiomide C (m/z 341.161 rt 20.1) and anacine (m/z 343.174 rt 12.7) are detected in both *P. verrucosum* and *P.nordicum*, unlike verrucines that are only detected in *P. verrucosum*. MineMS2 finds the same pattern in both datasets, including aurantiomide C and anacine. It contains the mass difference of NH_3 and CO (CH_3ON in the graph), and is present in 8 metabolites in this dataset, including 3 metabolites that are not quinazolines, but fungisporins.

MS2LDA also finds a pattern containing the neutral losses NH_3 and CO, that is however present in the 4 quinazolines only: aurantiomide C, anacine and their isomers.

Spectra

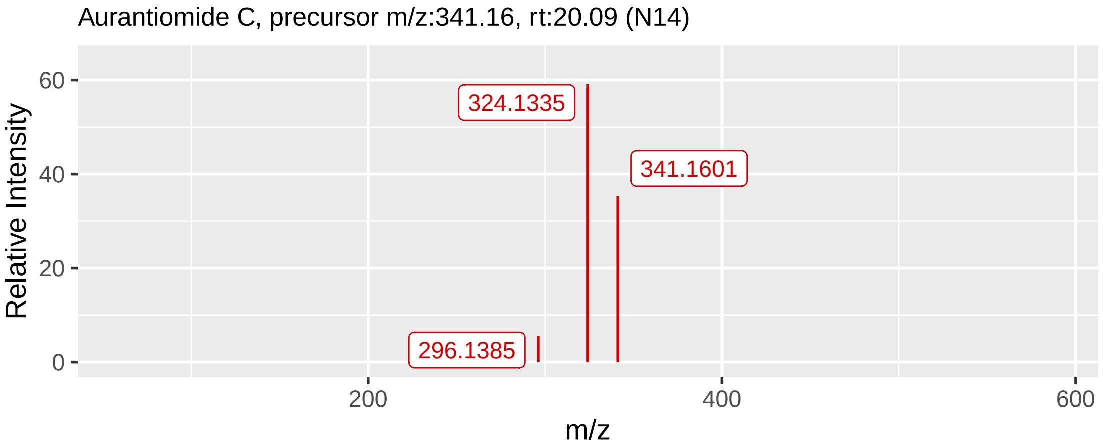

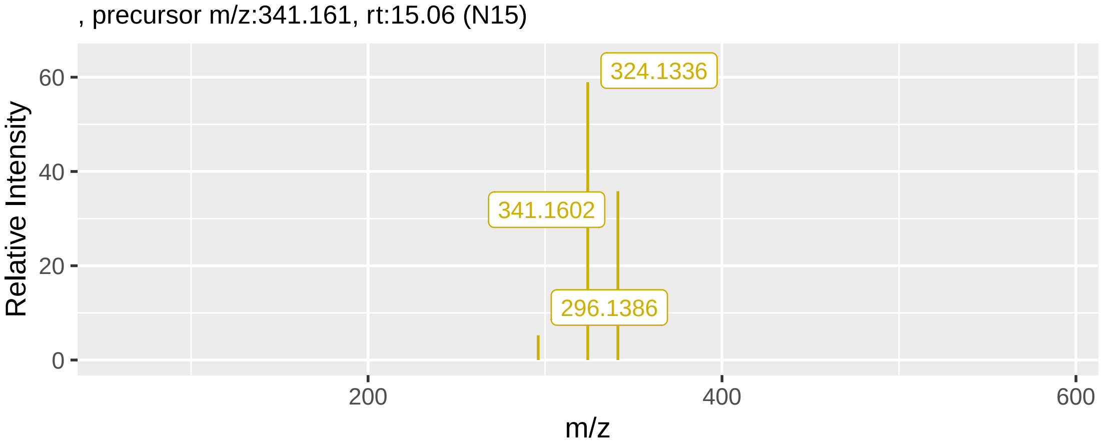

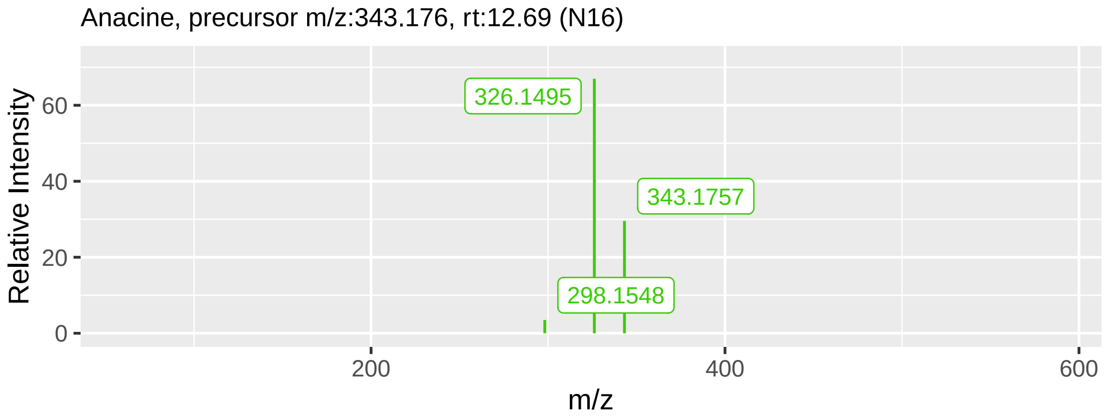
Anacine, precursor m/z:343.176, rt:12.69 (N16)
Relative Intensity
m/z
326.1495
343.1757
298.1548
0
20
40
60
200
400
600

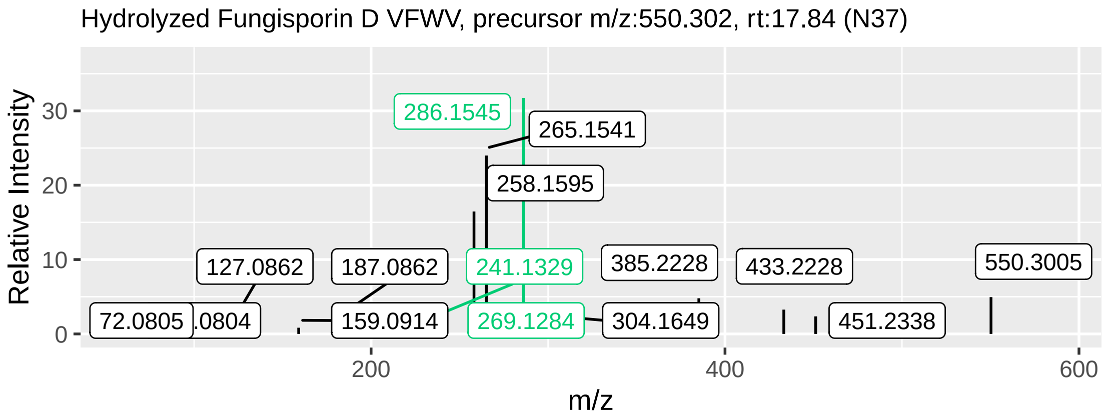
Hydrolyzed Fungisporin D VFWV, precursor m/z:550.302, rt:17.84 (N37)
Relative Intensity
m/z
286.1545
265.1541
258.1595
127.0862
187.0862
241.1329
385.2228
433.2228
550.3005
72.0805
.0804
159.0914
269.1284
304.1649
451.2338
0
10
20
30
200
400
600

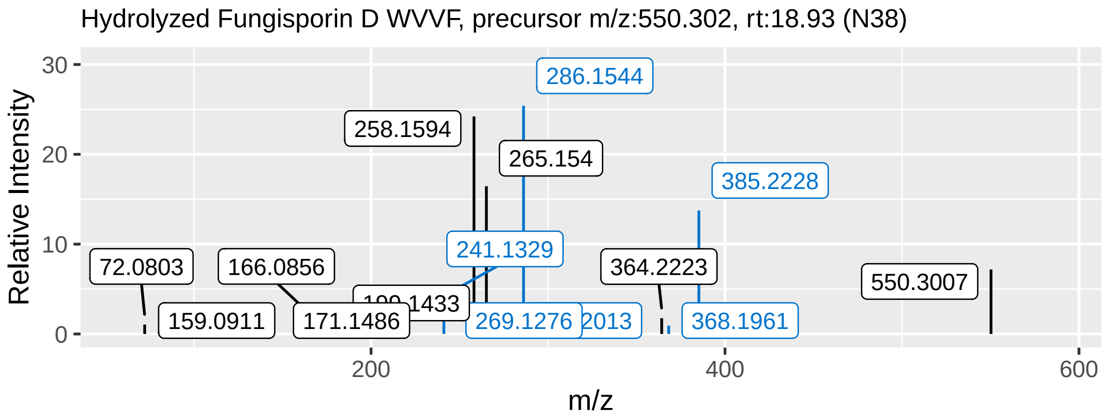
Hydrolyzed Fungisporin D WVVF, precursor m/z:550.302, rt:18.93 (N38)
Relative Intensity
m/z
286.1544
258.1594
265.154
385.2228
241.1329
72.0803
166.0856
364.2223
550.3007
.1433
159.0911
171.1486
269.1276
2013
368.1961
0
10
20
30
200
400
600

Hydrolyzed Cyclo(YWVV) WVVY, precursor m/z:566.297, rt:13.24 (N40)

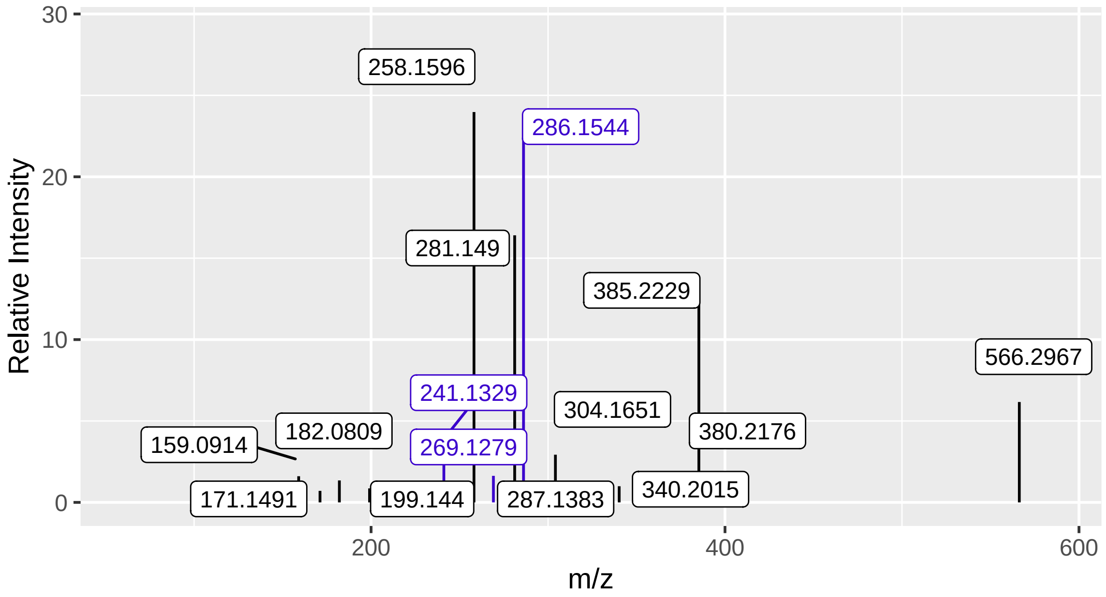

, precursor m/z:343.176, rt:17.83 (N48)

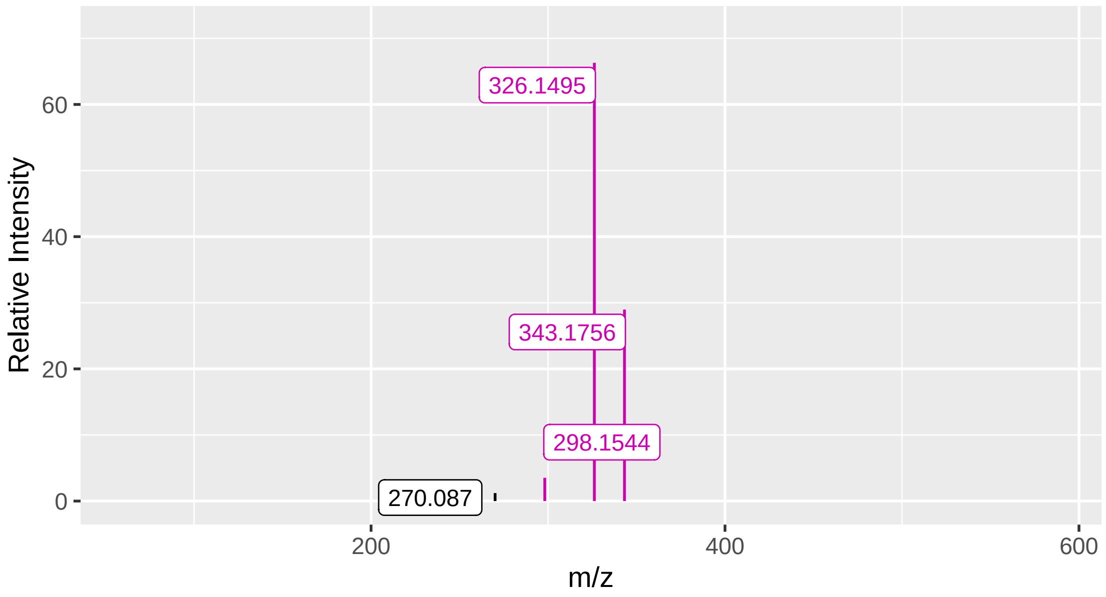

Fragmentation pattern

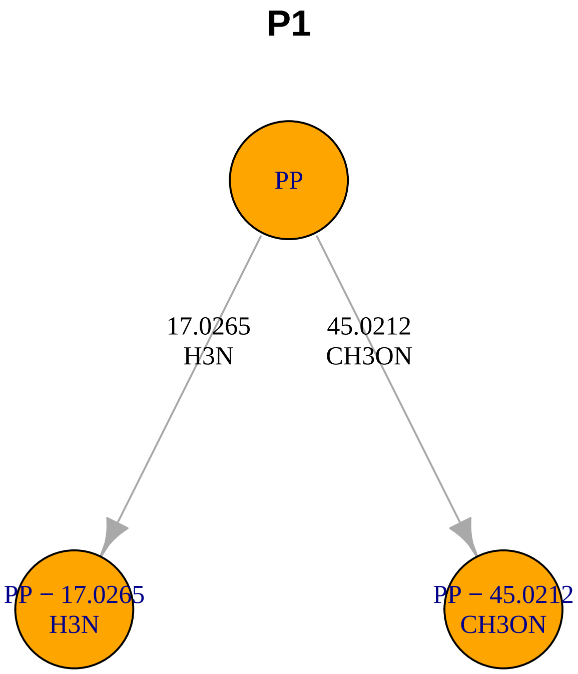

MS2LDA pattern 174

Feature	m/z mean	m/z min	m/z max	Probability
loss	17.0275	17.025	17.03	0.948
loss	45.0225	45.020	45.25	0.041

Metabolites containing this pattern

Precursor m/z	Retention time	Name	Probability
343.1761	12.69	Anacine	0.404
343.1761	17.83		0.399
341.1606	15.06		0.365
341.1601	20.09	Aurantiomide C	0.365

Fungisporin D and unknown ion at m/z 525.2682 rt 25.8

As observed in the publication (Hautbergue et al., 2019), fungisporin D (m/z 532.2908 rt 36.36; tetrapeptide antibiotic FWVV) and the unannotated metabolite at m/z 525.2682 rt 25.8 share seven common fragment ions and two losses, corresponding to the elimination of valine and phenylalanine respectively. A specific mineMS2 pattern is found for these two metabolites. As expected, the pattern contains the losses of valine (*dmz* 99.0682) and phenylalanine (*dmz* 147.0683; edges in green in the graph). Interestingly, the 8 ions in the pattern are all distinct from the 7 fragments listed in the publication: m/z 72.0804, 120.0809, 171.1485, 199.1439, 219.1485, 247.1436, 346.2115.

No common MS2LDA pattern was found for these two metabolites.

Spectra

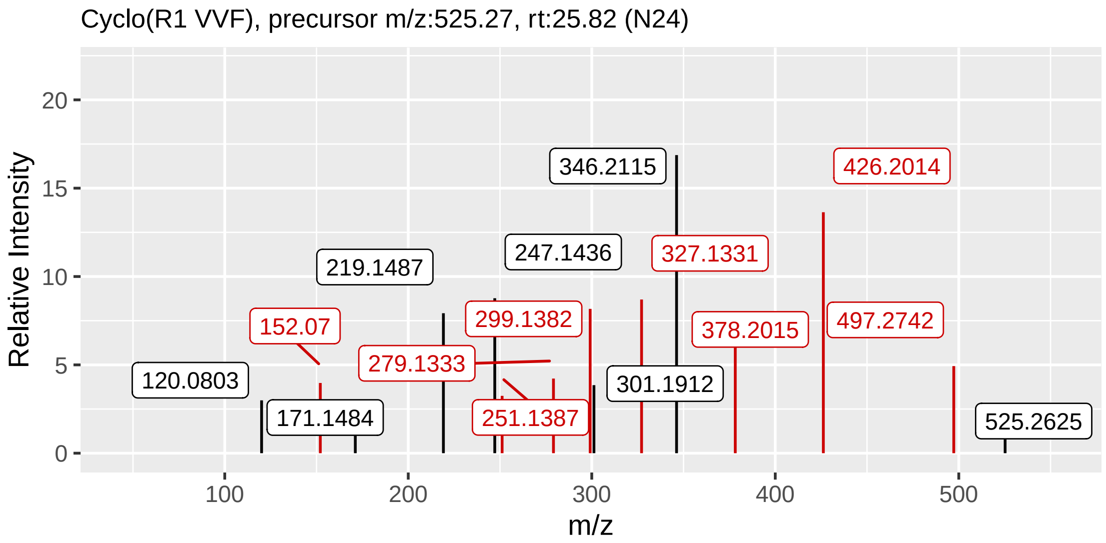

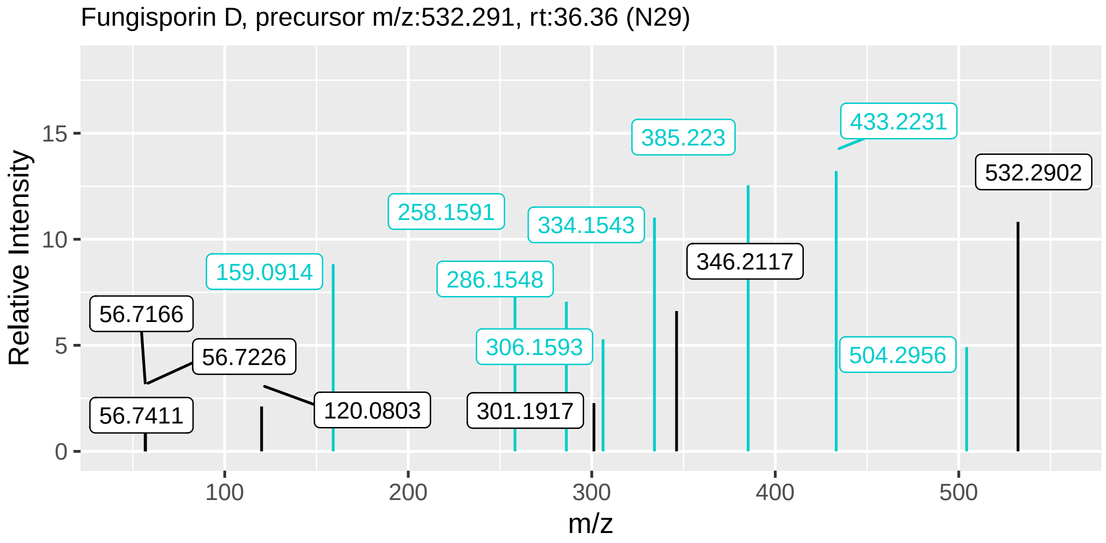

Fragmentation pattern

P142

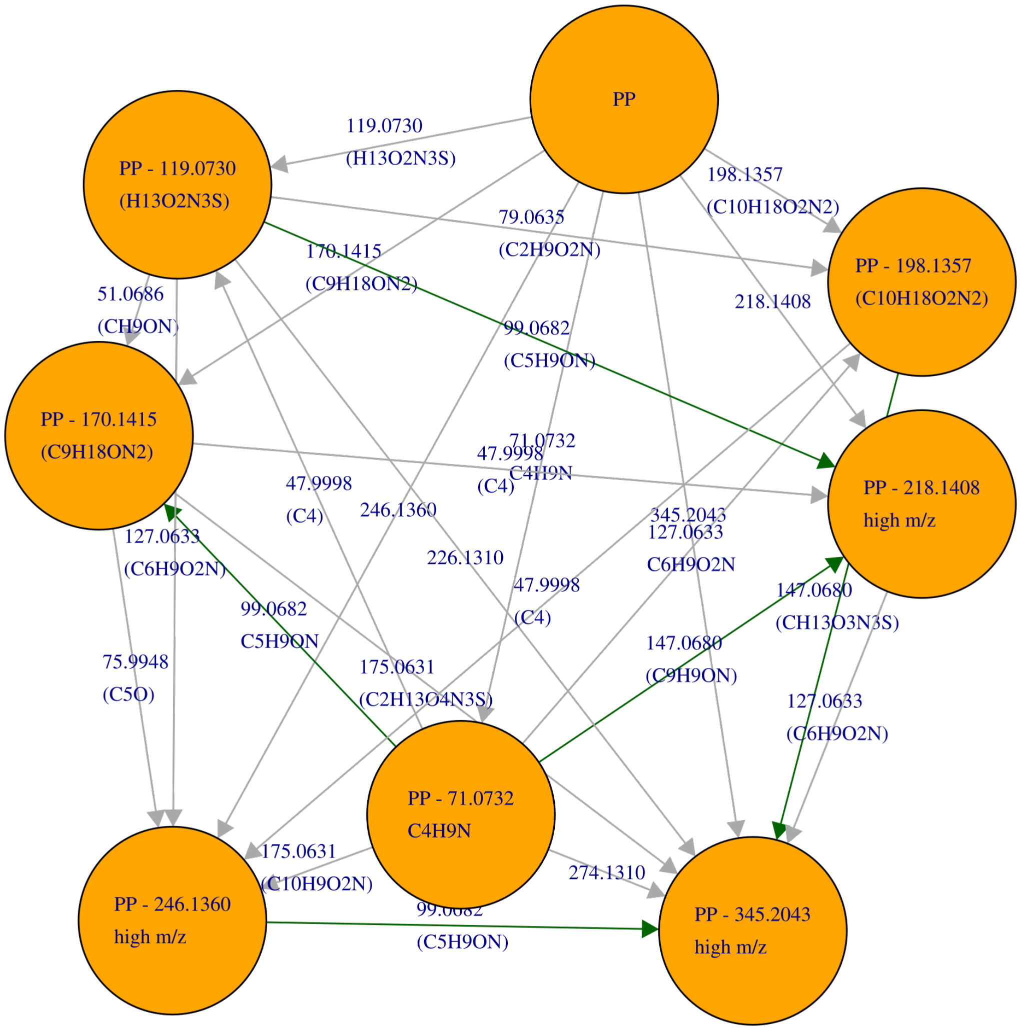

No MS2LDA pattern found.

Fungisporins related metabolites

In the original publication, 21 metabolites have been annotated as part of a family called fungisporins. These metabolites are cyclic or linear tetrapeptides, especially containing valine and phenylalanine residues. 6 of them had not yet been characterized prior to Hautbergue *et al.*, 2019. All the 21 metabolites are linked together in the GNPS network. The best mineMS2 fragmentation pattern, in terms of F1-score, is composed of a loss of valine (*dmz* 99.0683) and is present in 19 out of the 21 spectra. It can be noted that another pattern is also found, containing a loss of phenylalanine (*dmz* 147.068), present in 15 out of the 21 spectra, and in ochratoxins too. However, no mineMS2 pattern contains both a loss of valine and a loss of phenylalanine, and is present in more than 5 fungisporins.

The MS2LDA best pattern groups 10 fungisporins and contains a single fragment (m/z between 265.150 and 265.155).
This fragment could correspond to a dipeptide of valine and phenylalanine.

Spectra

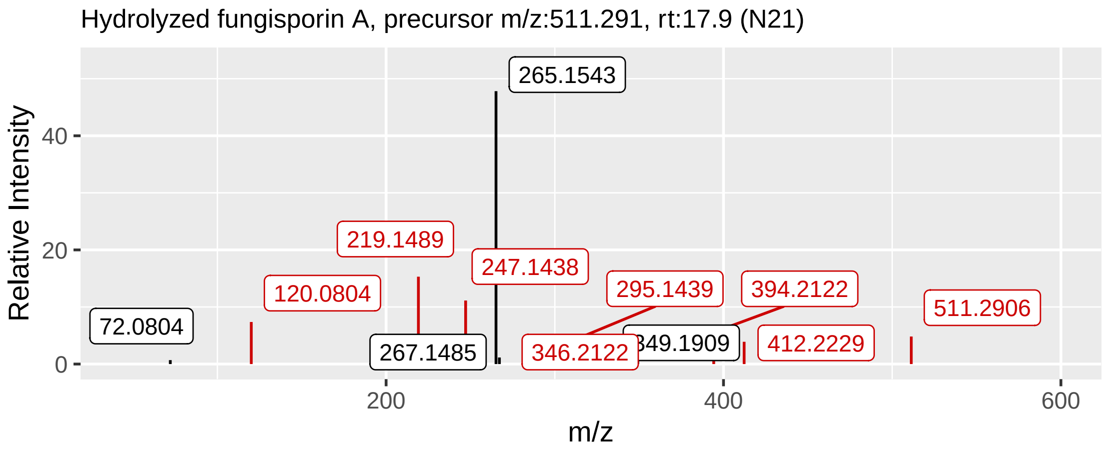

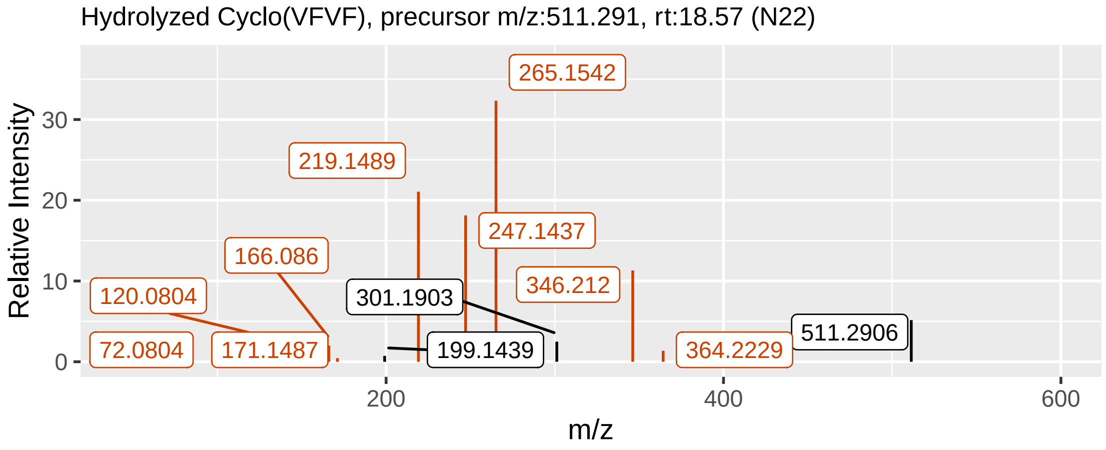

Cyclo(R1 VVF), precursor m/z:525.27, rt:25.82 (N24)

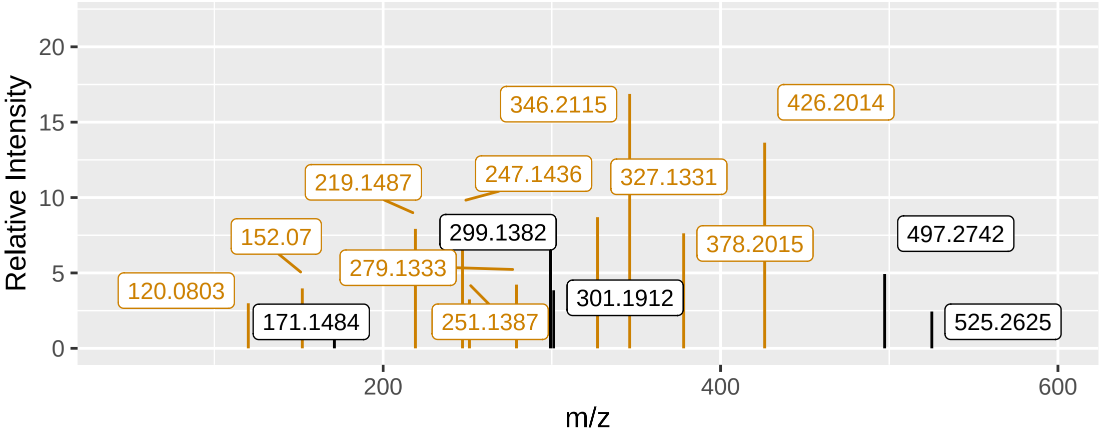

Hydrolyzed Cyclo(FFVI) IFFV, precursor m/z:525.306, rt:19.55 (N25)

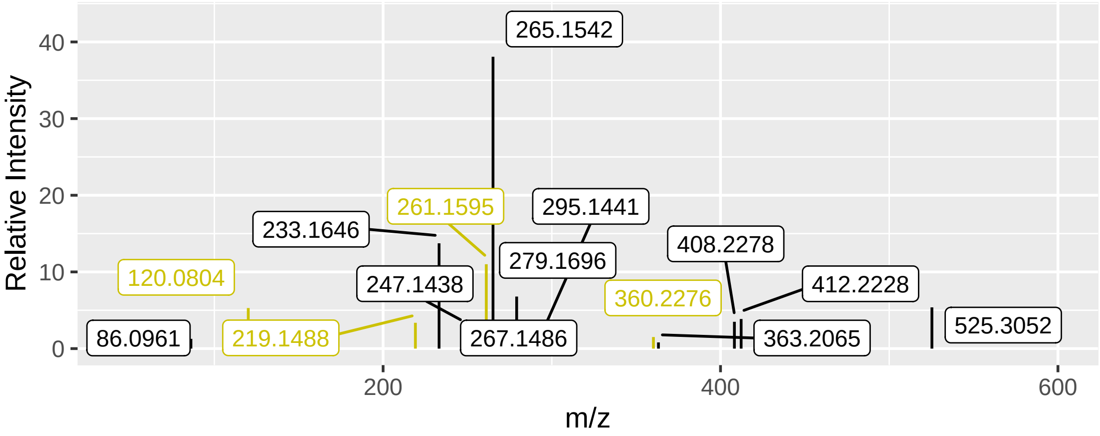

Hydrolyzed Cyclo(FFIV) VFFI, precursor m/z:525.306, rt:20.2 (N26)

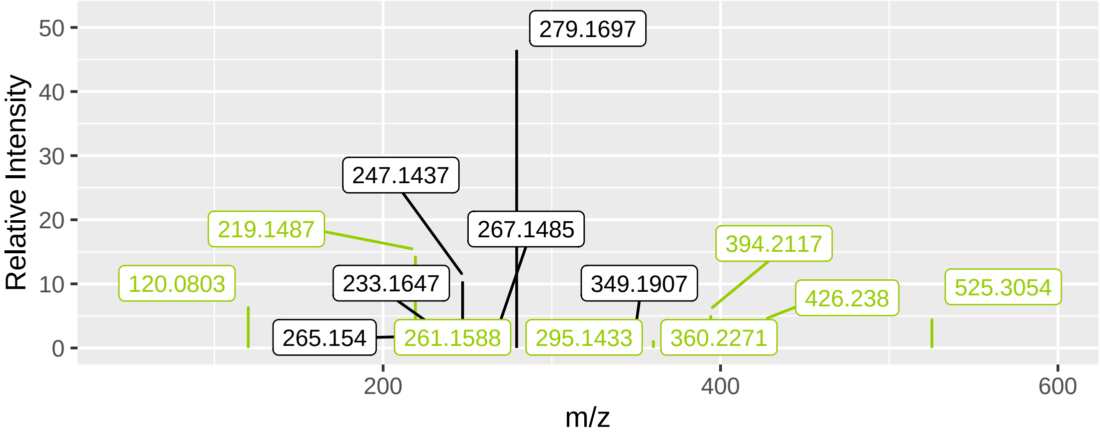

, precursor m/z:525.306, rt:20.92 (N27)

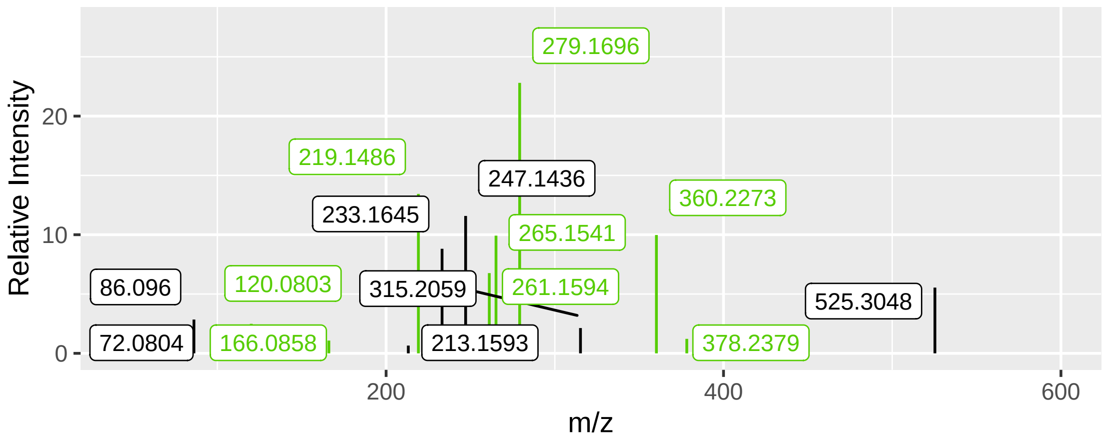

Hydrolyzed fungisporin B, precursor m/z:527.285, rt:12.75 (N28)

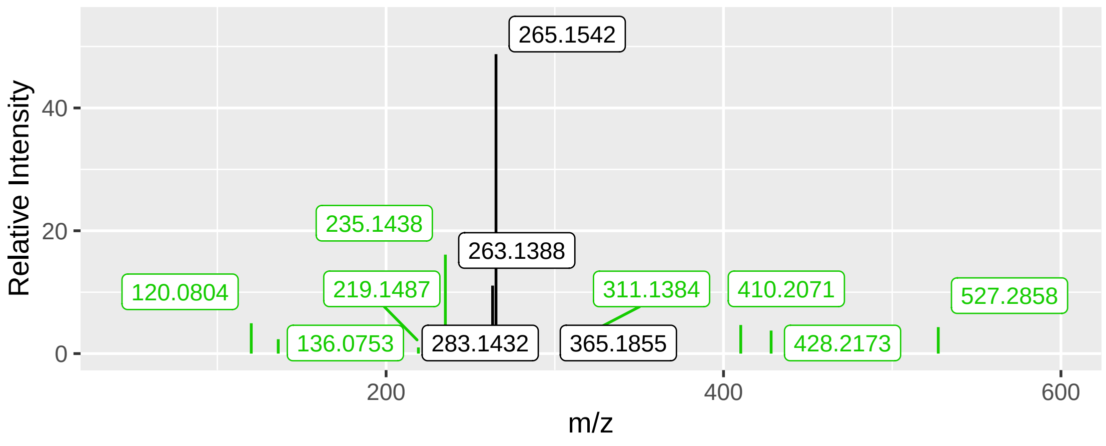

Fungisporin D, precursor m/z:532.291, rt:36.36 (N29)

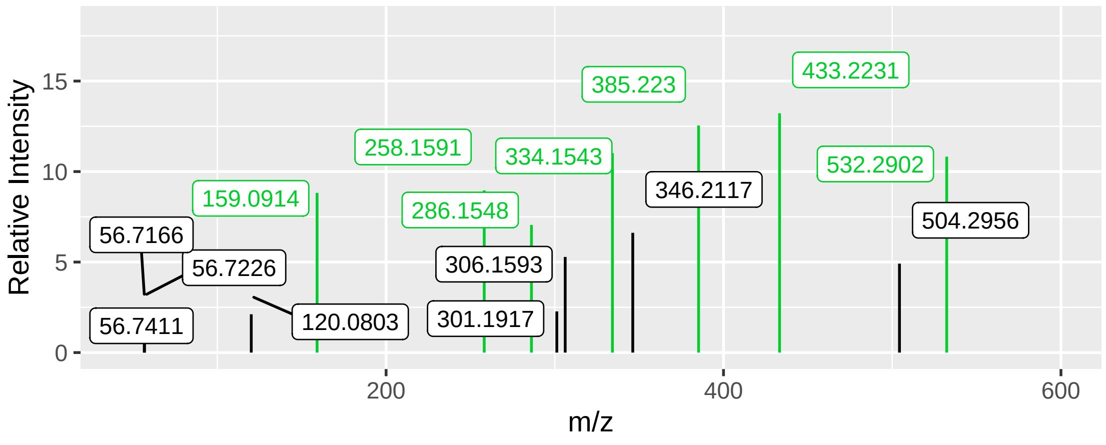

Hydrolyzed Cyclo(VR2FV) VR2FV, precursor m/z:537.306, rt:23.07 (N31)

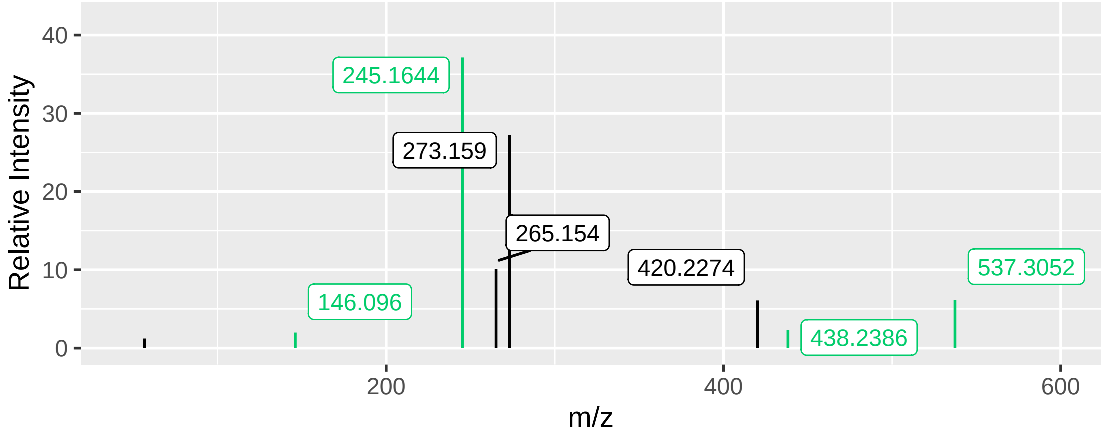

Hydrolyzed Cyclo(YFIV), precursor m/z:541.299, rt:15.43 (N33)

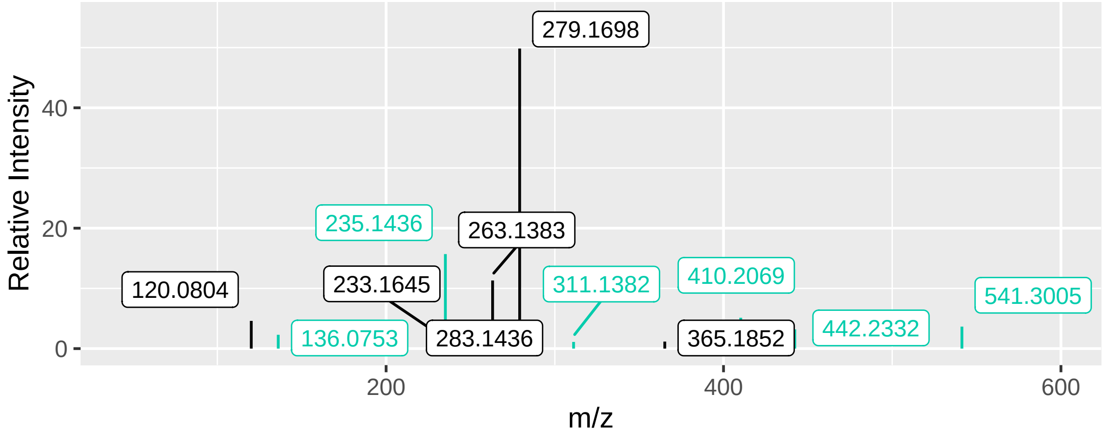

Hydrolyzed Cyclo(YFVI), precursor m/z:541.299, rt:13.84 (N34)

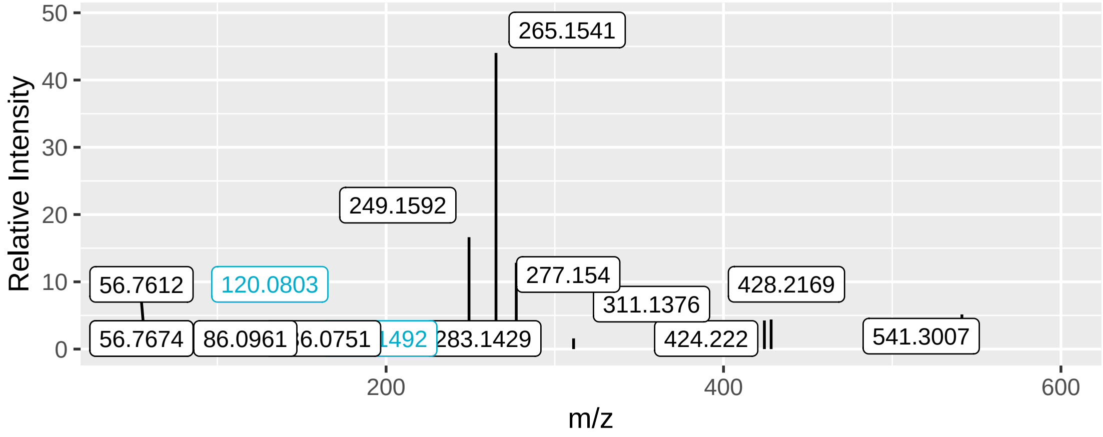

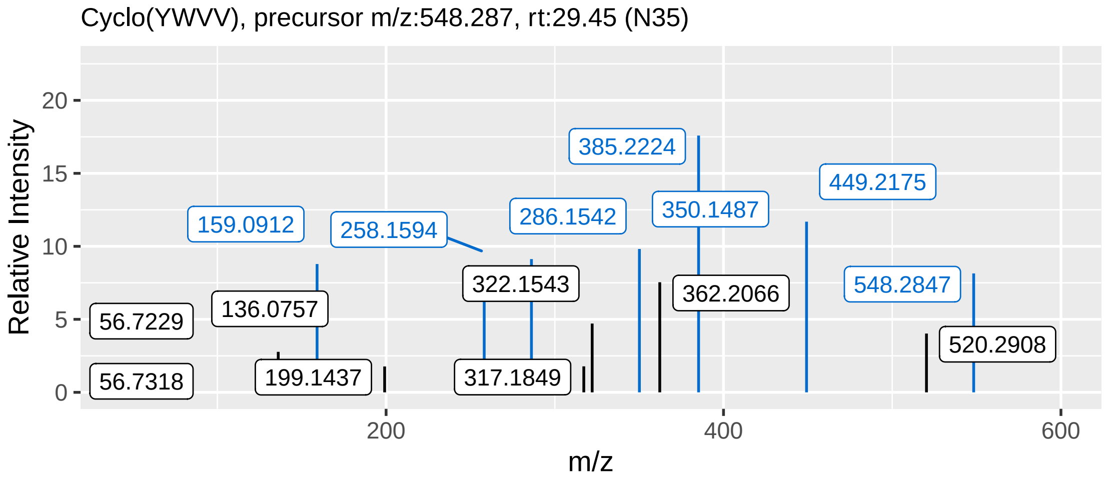
Cyclo(YWVV), precursor m/z:548.287, rt:29.45 (N35)
Relative Intensity
m/z
0
5
10
15
20
200
400
600
385.2224
449.2175
159.0912
258.1594
286.1542
350.1487
322.1543
362.2066
548.2847
56.7229
136.0757
520.2908
56.7318
199.1437
317.1849

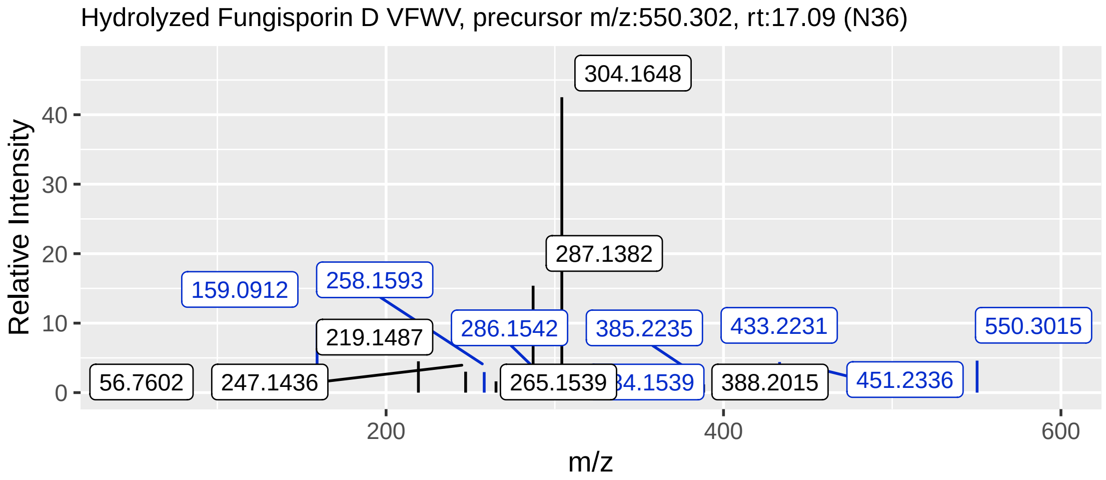
Hydrolyzed Fungisporin D VFWV, precursor m/z:550.302, rt:17.09 (N36)
Relative Intensity
m/z
0
10
20
30
40
200
400
600
304.1648
287.1382
159.0912
258.1593
219.1487
286.1542
385.2235
433.2231
550.3015
56.7602
247.1436
265.1539
388.2015
451.2336

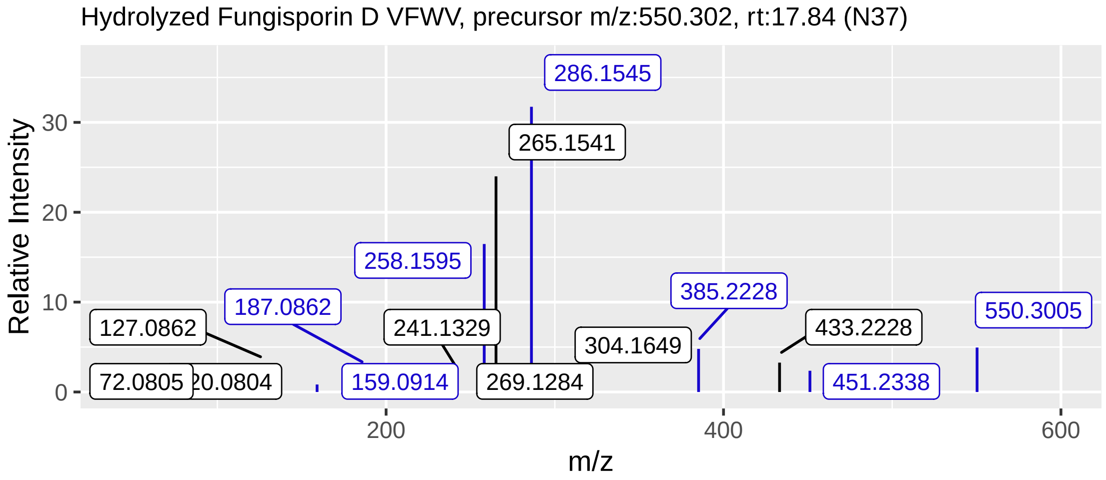
Hydrolyzed Fungisporin D VFWV, precursor m/z:550.302, rt:17.84 (N37)
Relative Intensity
m/z
0
10
20
30
200
400
600
286.1545
265.1541
258.1595
385.2228
187.0862
127.0862
241.1329
433.2228
550.3005
304.1649
72.0805
159.0914
269.1284
451.2338

Hydrolyzed Fungisporin D WVVF, precursor m/z:550.302, rt:18.93 (N38)

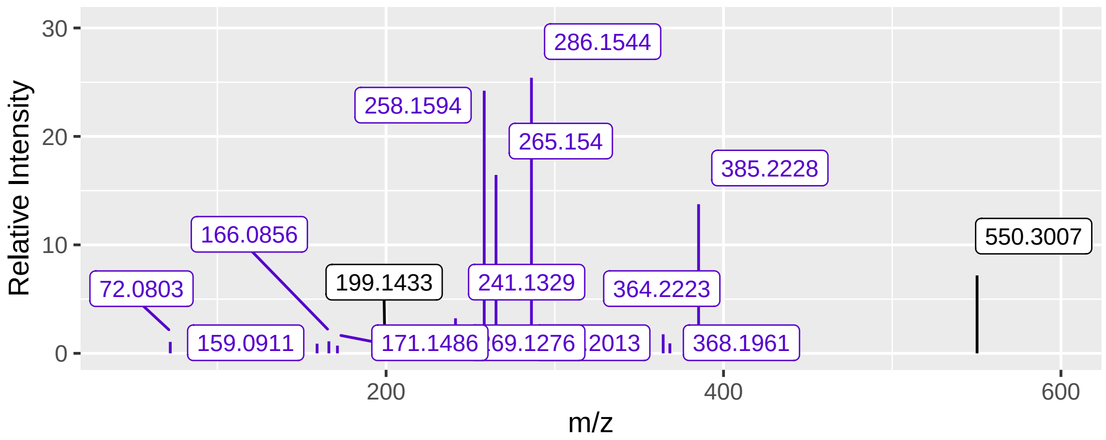

Hydrolyzed Cyclo(YWVV)VYWV, precursor m/z:566.297, rt:12.26 (N39)

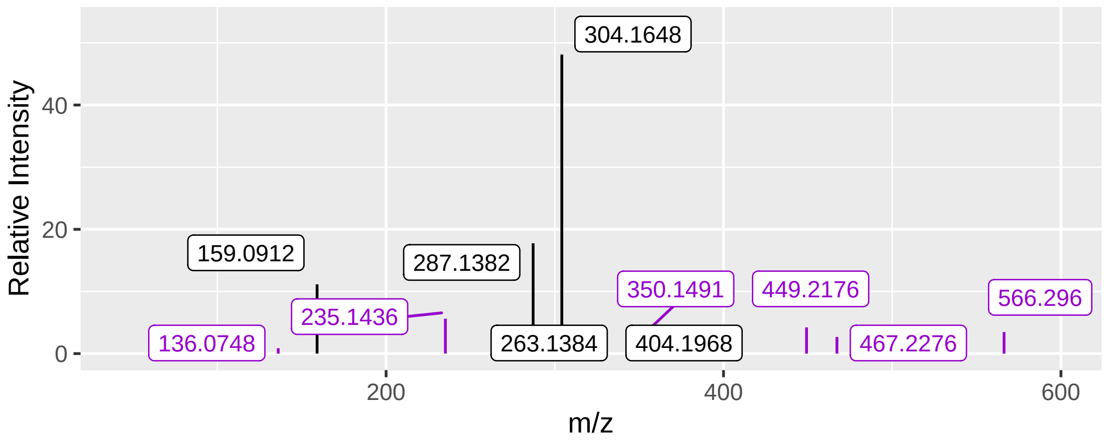

Hydrolyzed Cyclo(YWVV) WVVY, precursor m/z:566.297, rt:13.24 (N40)

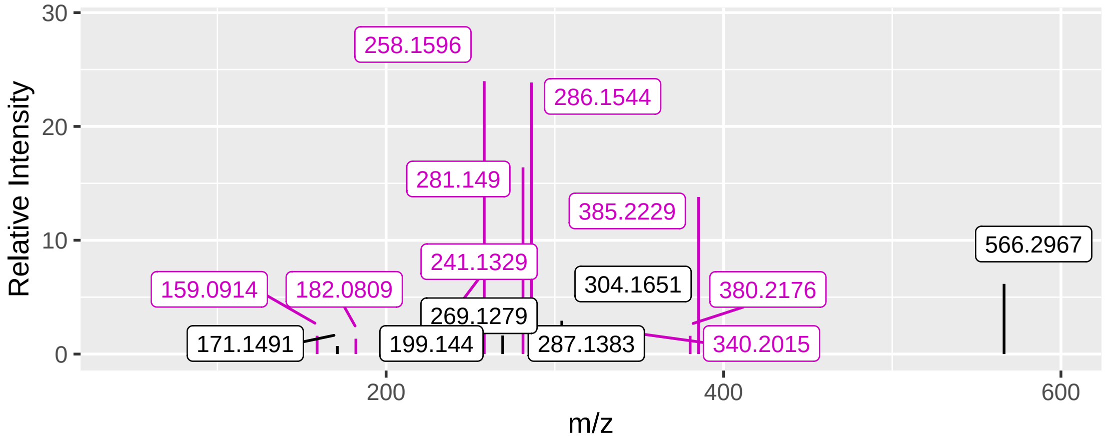

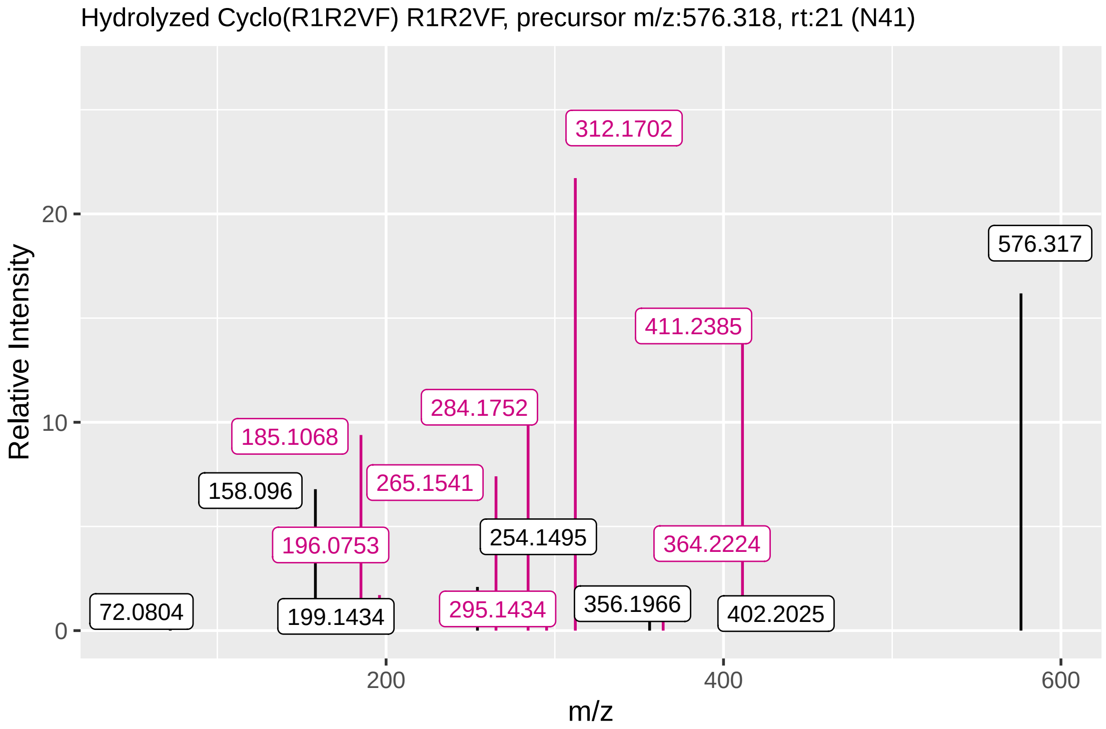
Hydrolyzed Cyclo(R1R2VF) R1R2VF, precursor m/z:576.318, rt:21 (N41)
Relative Intensity
m/z
0
10
20
200
400
600
312.1702
576.317
411.2385
284.1752
185.1068
265.1541
158.096
254.1495
196.0753
364.2224
356.1966
72.0804
295.1434
402.2025
199.1434

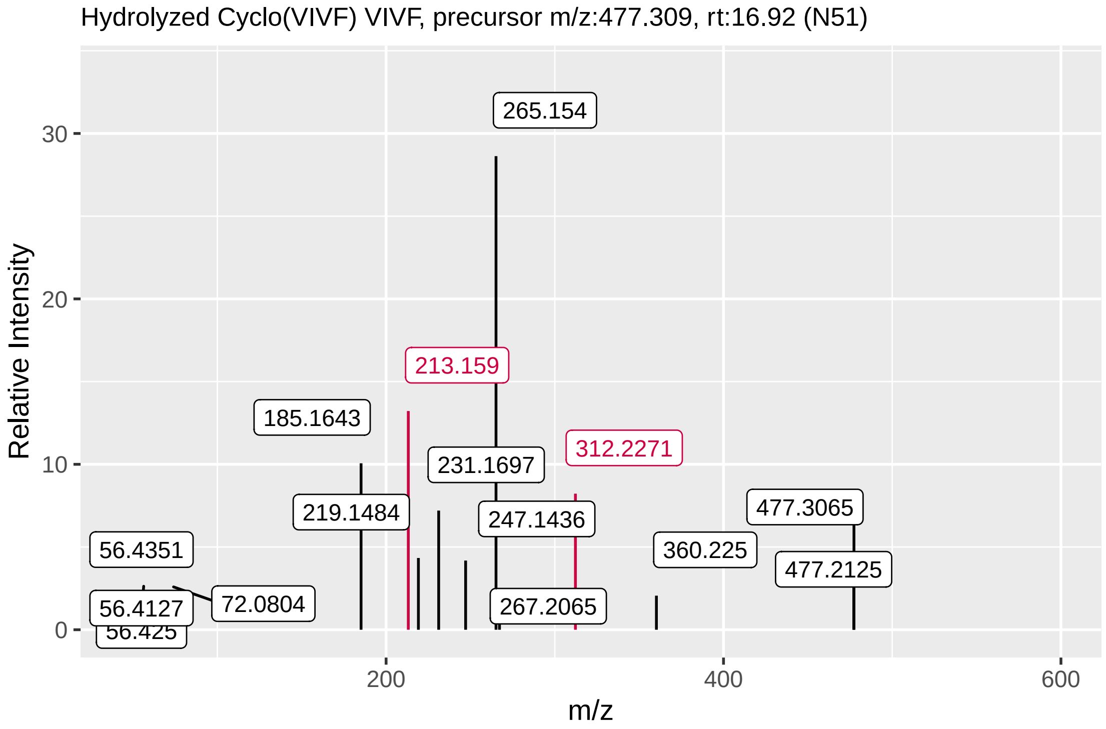
Hydrolyzed Cyclo(VIVF) VIVF, precursor m/z:477.309, rt:16.92 (N51)
Relative Intensity
m/z
0
10
20
30
200
400
600
265.154
213.159
185.1643
312.2271
231.1697
219.1484
247.1436
477.3065
56.4351
360.225
477.2125
72.0804
56.4127
267.2065
56.425

Fragmentation pattern

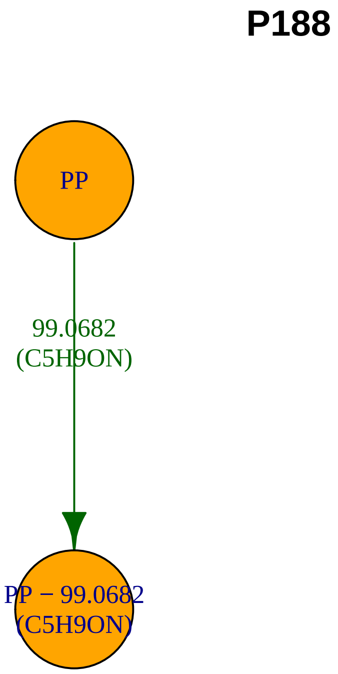

MS2LDA pattern 212

Feature	m/z mean	m/z min	m/z max	Probability
fragment	265.1525	265.15	265.155	0.995

Metabolites containing this pattern

Precursor m/z	Retention time	Name	Probability
477.3090	16.92	Hydrolyzed Cyclo(VIVF) VIVF	0.294
511.2906	17.90	Hydrolyzed fungisporin A	0.433
511.2906	18.57	Hydrolyzed Cyclo(VFVF)	0.285
525.3060	19.55	Hydrolyzed Cyclo(FFVI) IFFV	0.346
525.3060	20.92		0.084
527.2853	12.75	Hydrolyzed fungisporin B	0.445
537.3061	23.07	Hydrolyzed Cyclo(VR2FV) VR2FV	0.100
541.2990	13.84	Hydrolyzed Cyclo(YFVI)	0.418
550.3015	17.84	Hydrolyzed Fungisporin D VFWV	0.218
550.3015	18.93	Hydrolyzed Fungisporin D WVVF	0.135

Figure S1: mineMS2 execution time as a function of the number of spectra.

LC-HRMS/MS spectra acquired in the positive mode with either (a) a quadrupole Time-Of-Flight (qTOF; CID mode; collision energy: 40 eV), or (b) an Orbitrap (HCD mode; normalized collision energy: 35%) mass analyzer were extracted from the FragHub database, which integrates the main public libraries [Dablanc et al., 2024]. Computation times (measured on a Windows desktop with 64 Gb memory and a 24-core Intel Core i9-13950HX 2.2 GHz processor) of the two main steps of the algorithm are indicated (as well as the total execution time): 1) the DAG creation (binning of m/z differences, formula generation and DAG conversion from spectra), and 2) the mining of frequent subgraphs within the DAGs.

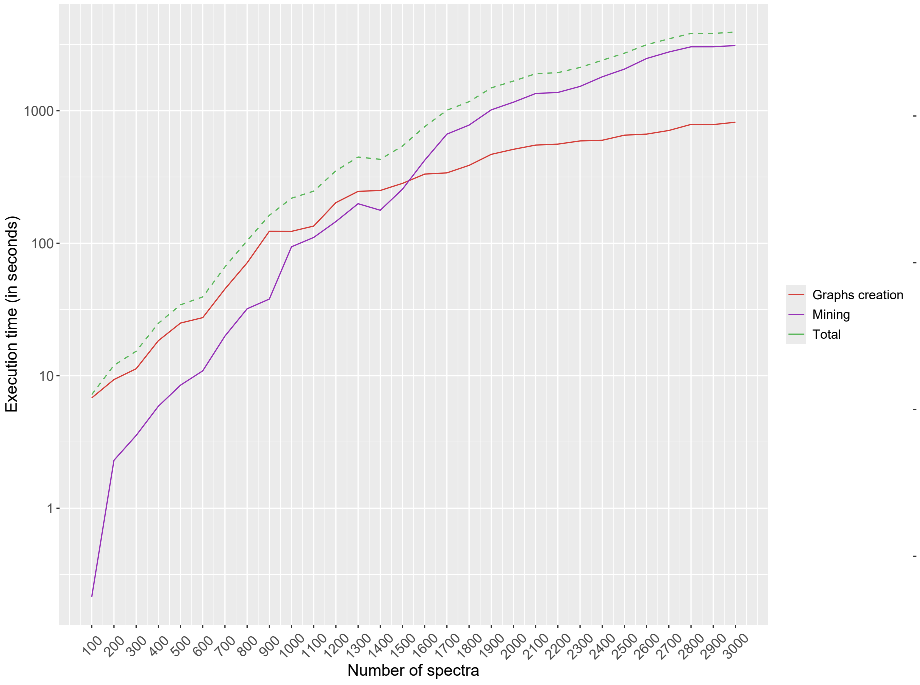

(a) qTOF

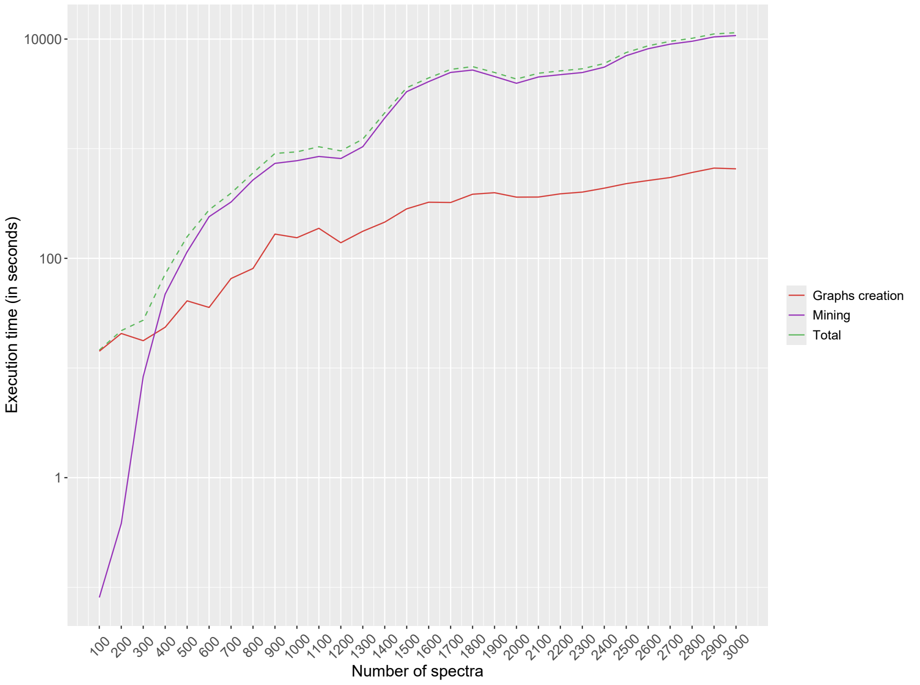

(b) Orbitrap

Figure S2: Number of ChemOnt concepts explained by mineMS2 and MS2LDA at a given precision or recall level.

The number of ChemOnt concepts for which at least one pattern from mineMS2 or MS2LDA exists with a precision or recall above a specified threshold is shown (left plot). Interestingly, a closer inspection of the MS2LDA patterns best explaining ChemOnt concepts at a precision above 0.5 indicates that the patterns were actually included in a single molecule for half of the concepts. The curves obtained when only MS2LDA patterns detected in at least two spectra are considered, are shown on the right plot.

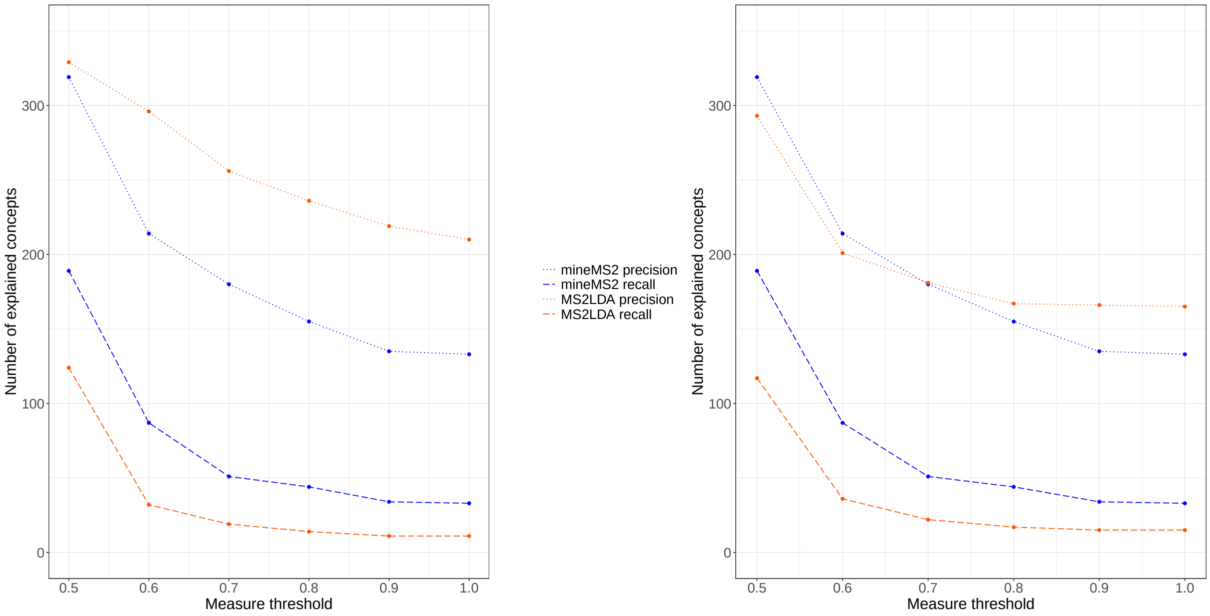

(a) all MS2LDA patterns (b) MS2LDA patterns found in at least two spectra

Number of ChemOnt concepts for which at least one pattern from mineMS2 (blue) or MS2LDA (orange) was found as a function of the precision (dotted line) or recall (dashed line) threshold. a) all MS2LDA patterns are considered (as in the Fig. 4 of the main article). b) MS2LDA patterns are restricted to those found in at least two spectra.

Figure S3: Explainability of ChemOnt concepts by mineMS2 patterns obtained with single or multi-energy (merged) spectral collections.

	**10% Energy**	**Merged energies**
**Mean F1**	0.53	0.57
**Mean recall**	0.48	0.51
**Mean precision**	0.70	0.74

(a) Mean values of F1-score, recall and precision for the explained ChemOnt concepts with single collision energy (HCD 10%) or multi-energy (merged) spectra collections from the LIMS-DB library.

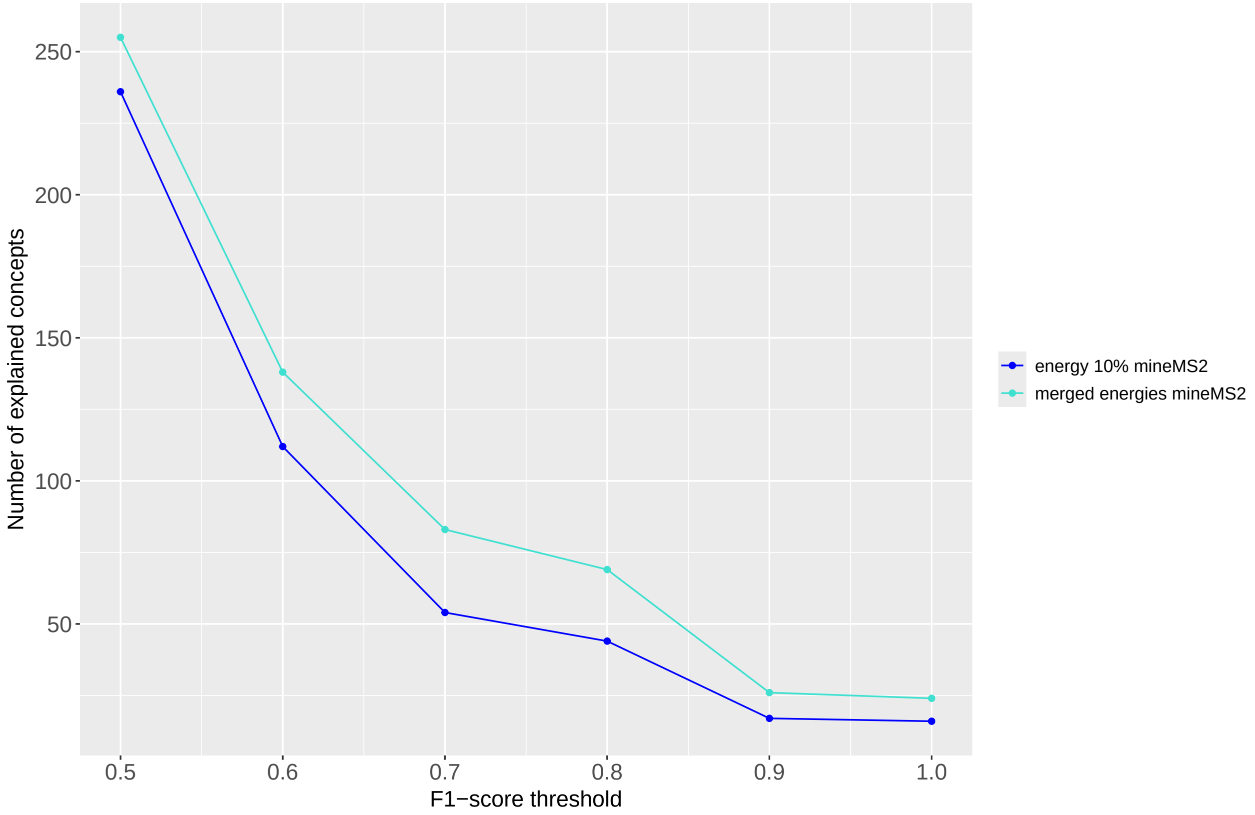

(b) Explained ChemOnt concepts by mineMS2 patterns obtained from single (blue) and multi-energy (turquoise) spectra, as a function of the F1-score threshold.

Figure S4: Best mineMS2 patterns explaining the ChemOnt concepts *Flavonols* and *Delta-5-steroids* obtained with the single and multi-energy spectra collections.

The concept *Flavonols* (respectively, *Delta-5-steroids*) is fully explained (F1-score = 1.0) by the pattern obtained with the merged (respectively, single energy) spectra, with the highest difference of F1-score values compared to the single energy (respectively, merged) spectra library.

For each concept, the following information is provided:

1. the best mineMS2 patterns obtained with both collections
2. a table summarizing whether the spectrum of each molecule contains the pattern, and the cosine similarity between the single energy and merged spectra
3. the merged (top) and single energy (bottom) spectrum of each molecule

Flavonols

ChemOnt id: CHEMONTID:0001136
Depth of taxonomy: 5
Description: Compounds that contain a flavone (2-phenyl-1-benzopyran-4-one) backbone carrying a hydroxyl group at the 3-position.

Patterns

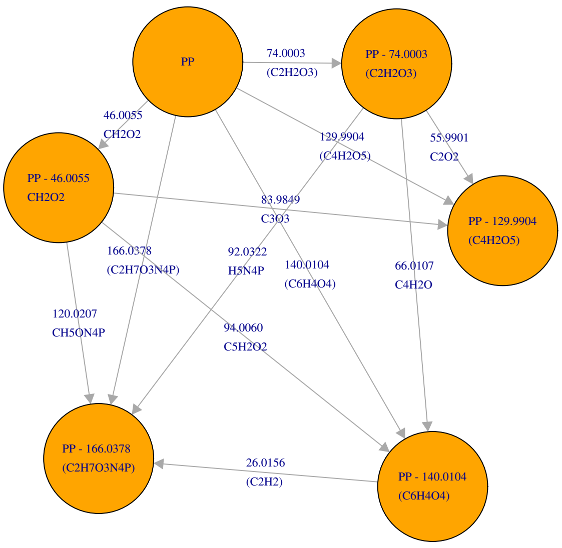

(a) merged energy pattern

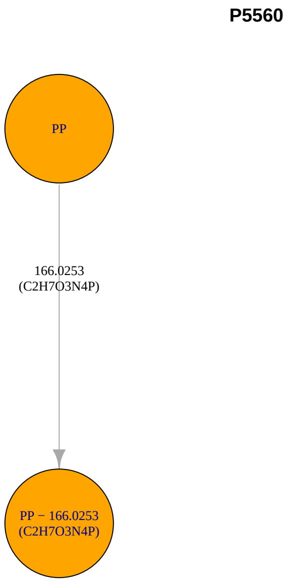

(b) 10% energy pattern

Molecules of the concepts

**Name**	**Precursor m/z**	**Formula**	**Structure**	**In merged energy pattern** precision = 1.0 recall = 1.0	**In 10% energy pattern** precision = 0.33 recall = 0.67	**Cosine score between spectra**
Kaempferol	287.0550	C15H10O6	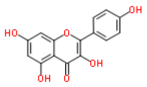	Yes	Yes	0.78
Myricetin	319.0448	C15H10O8	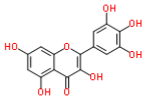	Yes	Yes	0.78
Quercetin	303.0499	C15H10O7	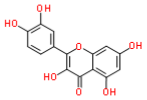	Yes	No	0.53

Spectra

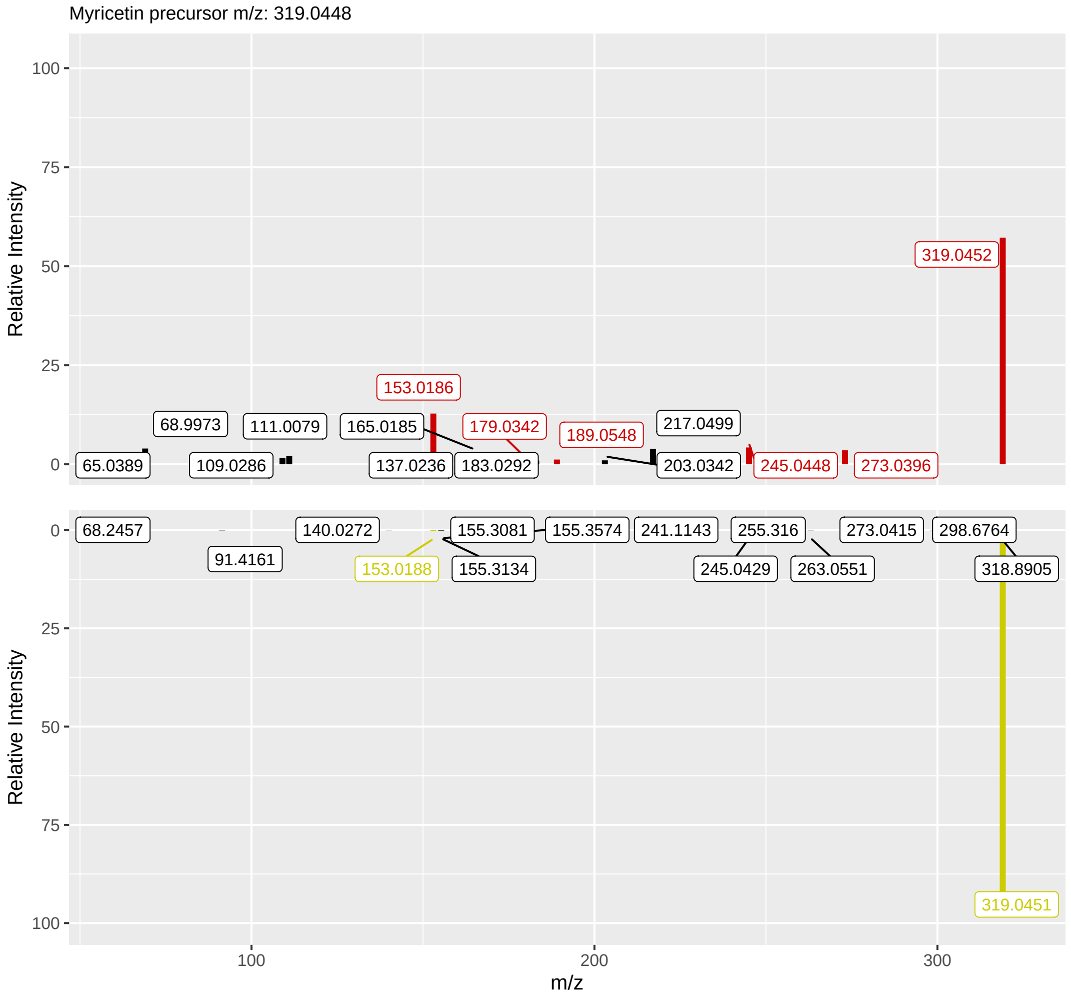

merged energy

10% energy

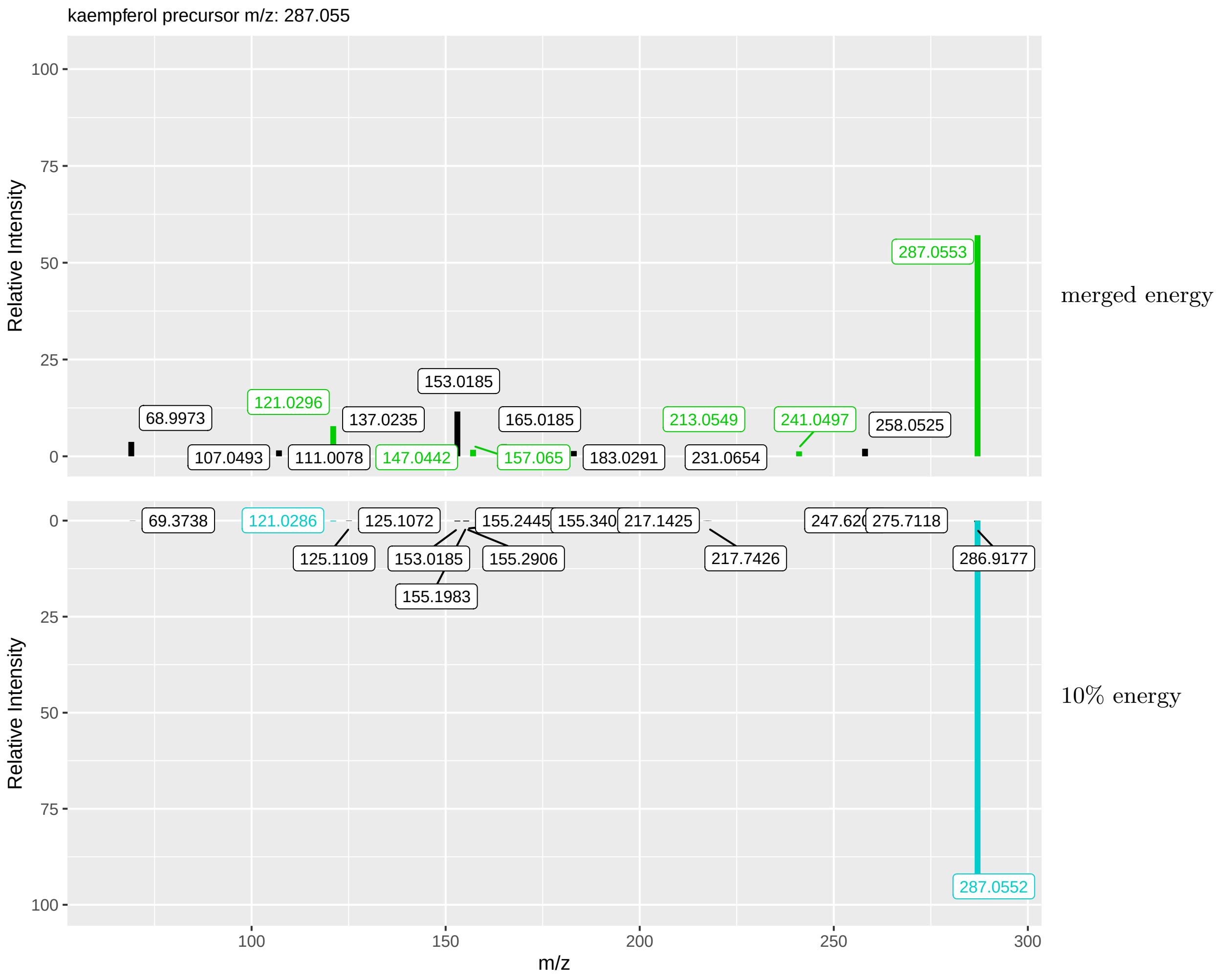
kaempferol precursor m/z: 287.055
Relative Intensity
m/z
287.0553
153.0185
121.0296
68.9973
137.0235
165.0185
213.0549
241.0497
258.0525
107.0493
111.0078
147.0442
157.065
183.0291
231.0654
69.3738
121.0286
125.1072
155.2445
155.340
217.1425
247.620
275.7118
125.1109
153.0185
155.2906
217.7426
286.9177
155.1983
287.0552
merged energy
10% energy

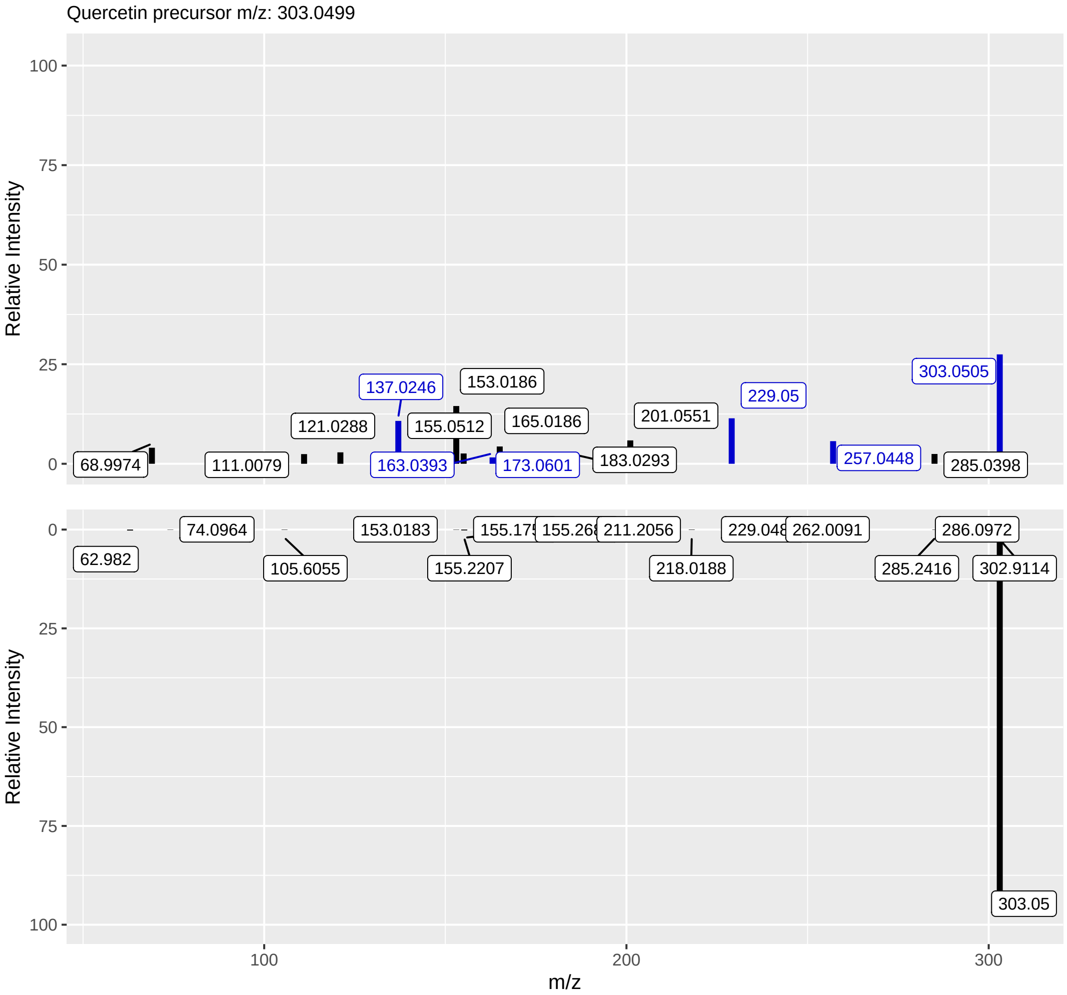

merged energy

10% energy

Delta-5-steroids

ChemOnt id: CHEMONTID:0002979
Depth of taxonomy: 4
Description: Steroids containing a double bond between positions 5 and 6.

Patterns

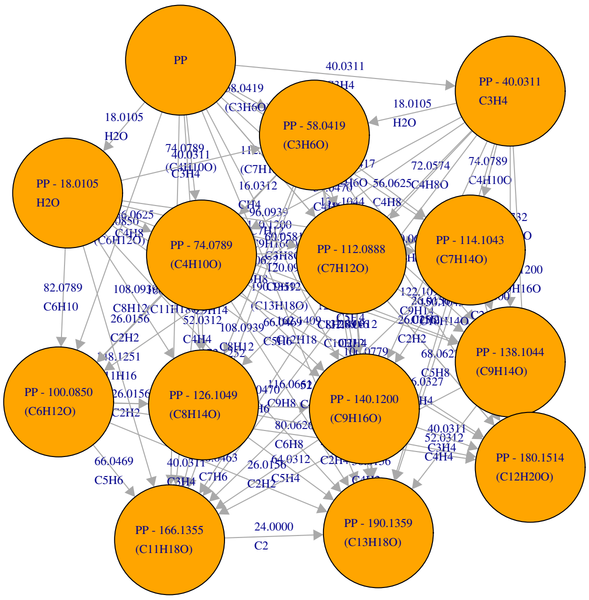

(a) merged energy pattern

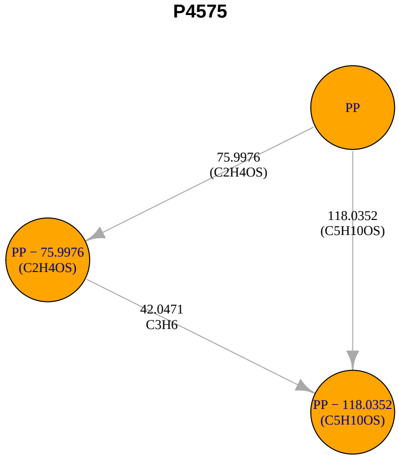

(b) 10% energy pattern

Molecules of the concepts

Name	Precursor m/z	Formula	Structure	In merged energy pattern precision = 1.0 recall = 0.67	In 10% energy pattern precision = 1.0 recall = 1.0	Cosine score between spectra
trans-Dehydro androsterone (DHEA)	289.2162	C19H28O	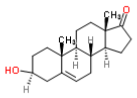	Yes	Yes	0.64
3-b-Hydroxyandrost-5-en-17-one (Dehydroepi-androsterone)	289.2162	C19H28O	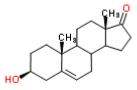	Yes	Yes	0.73
Pregnenolone	317.2475	C21H32O	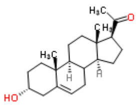	No	Yes	0.61

Spectra

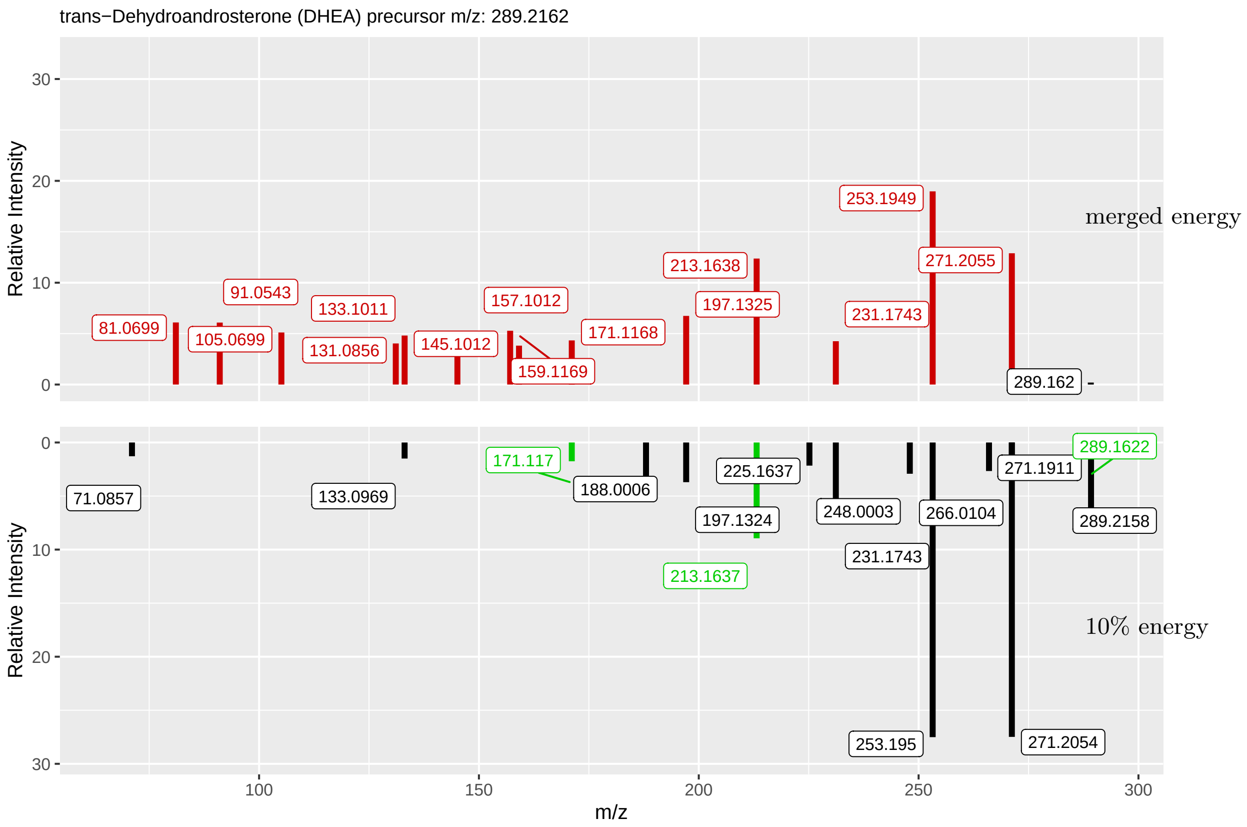
trans−Dehydroandrosterone (DHEA) precursor m/z: 289.2162
Relative Intensity
merged energy
253.1949
271.2055
213.1638
197.1325
231.1743
91.0543
133.1011
157.1012
81.0699
105.0699
131.0856
145.1012
171.1168
159.1169
289.162
171.117
188.0006
225.1637
197.1324
248.0003
266.0104
231.1743
213.1637
271.1911
289.1622
289.2158
71.0857
133.0969
10% energy
253.195
271.2054
m/z

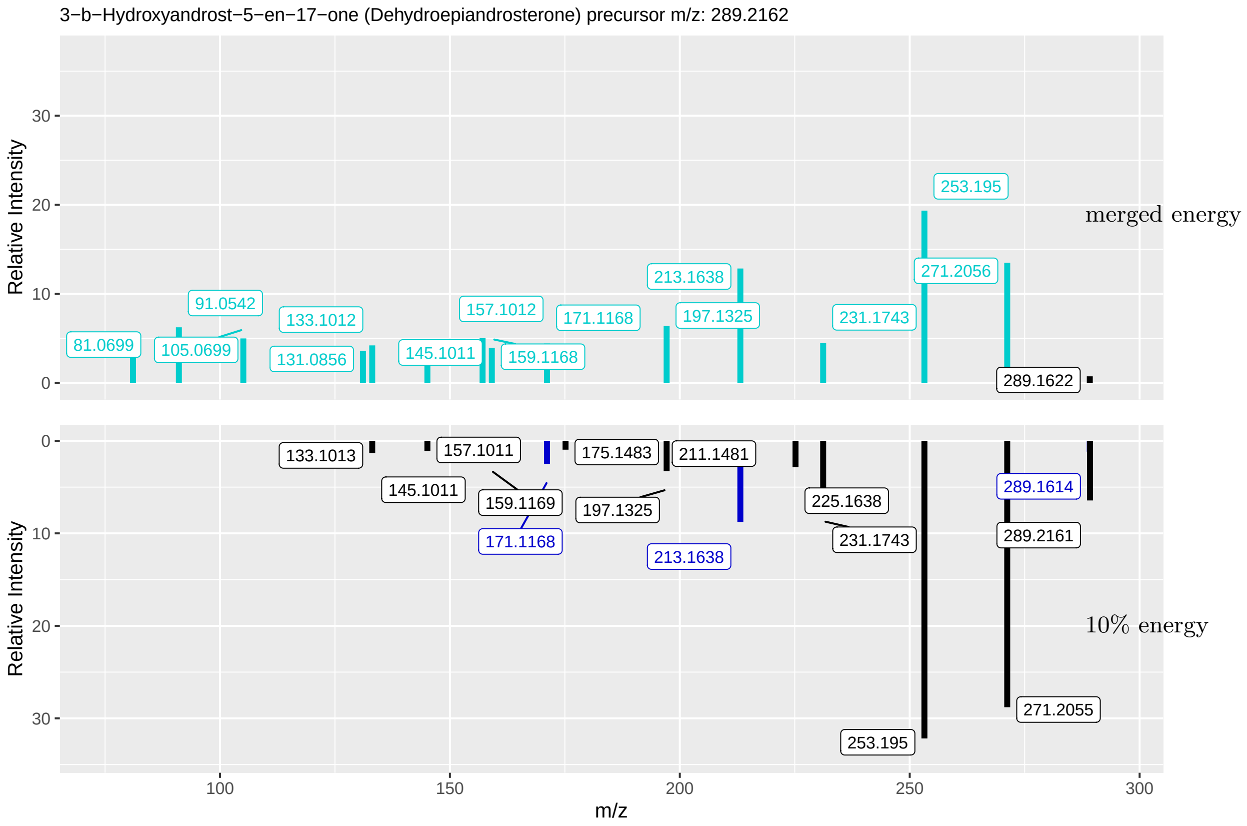
3−b−Hydroxyandrost−5−en−17−one (Dehydroepiandrosterone) precursor m/z: 289.2162
Relative Intensity
merged energy
253.195
271.2056
213.1638
197.1325
231.1743
91.0542
133.1012
157.1012
171.1168
81.0699
105.0699
131.0856
145.1011
159.1168
289.1622
133.1013
157.1011
175.1483
211.1481
145.1011
159.1169
197.1325
225.1638
171.1168
213.1638
231.1743
289.1614
289.2161
10% energy
271.2055
253.195
m/z

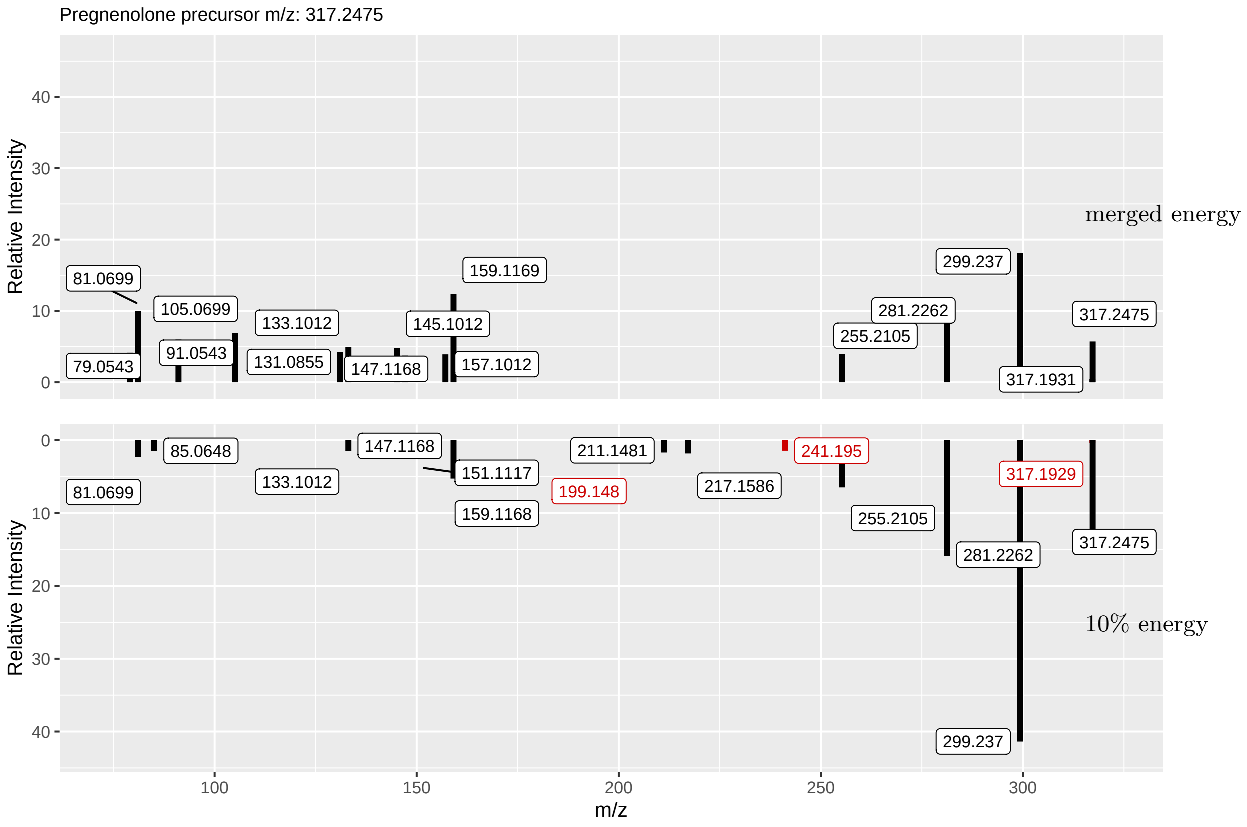
Pregnenolone precursor m/z: 317.2475
Relative Intensity
merged energy
81.0699
105.0699
133.1012
145.1012
159.1169
299.237
281.2262
255.2105
317.2475
79.0543
91.0543
131.0855
147.1168
157.1012
317.1931
85.0648
147.1168
211.1481
241.195
81.0699
133.1012
151.1117
199.148
217.1586
317.1929
159.1168
255.2105
281.2262
317.2475
10% energy
299.237
m/z

Figure S5: Example of the *Flavans* concept, composed of 4 metabolites, and its best explaining mineMS2 pattern.

mineMS2 finds a pattern that fully explains the *Flavans* concept (F1-score = 1). In the MS/MS spectra (a), colored peaks are included in the fragmentation pattern of mineMS2 (b). The table lists the m/z differences from the pattern, with the possible molecular formulas for each of them. For each m/z difference, the algorithm selects the formula with the m/z closest to the *dmz* value. It can be noted that the precursor peak is not always included in the pattern. Importantly, some m/z differences are probably due to parallel fragmentations, such as the *dmz* = 24 of formula C_2 contained in this pattern. c) The fragmentation trees predicted by SIRIUS [Böcker et al., 2009] further support this hypothesis, by all including a C_2 difference (shown in blue) between molecular formulas from parallel fragmentation paths. The above specificities of mineMS2 explain why MS2LDA, which focuses on neutral losses from the precursor ion, does not provide an equivalent pattern for these spectra.

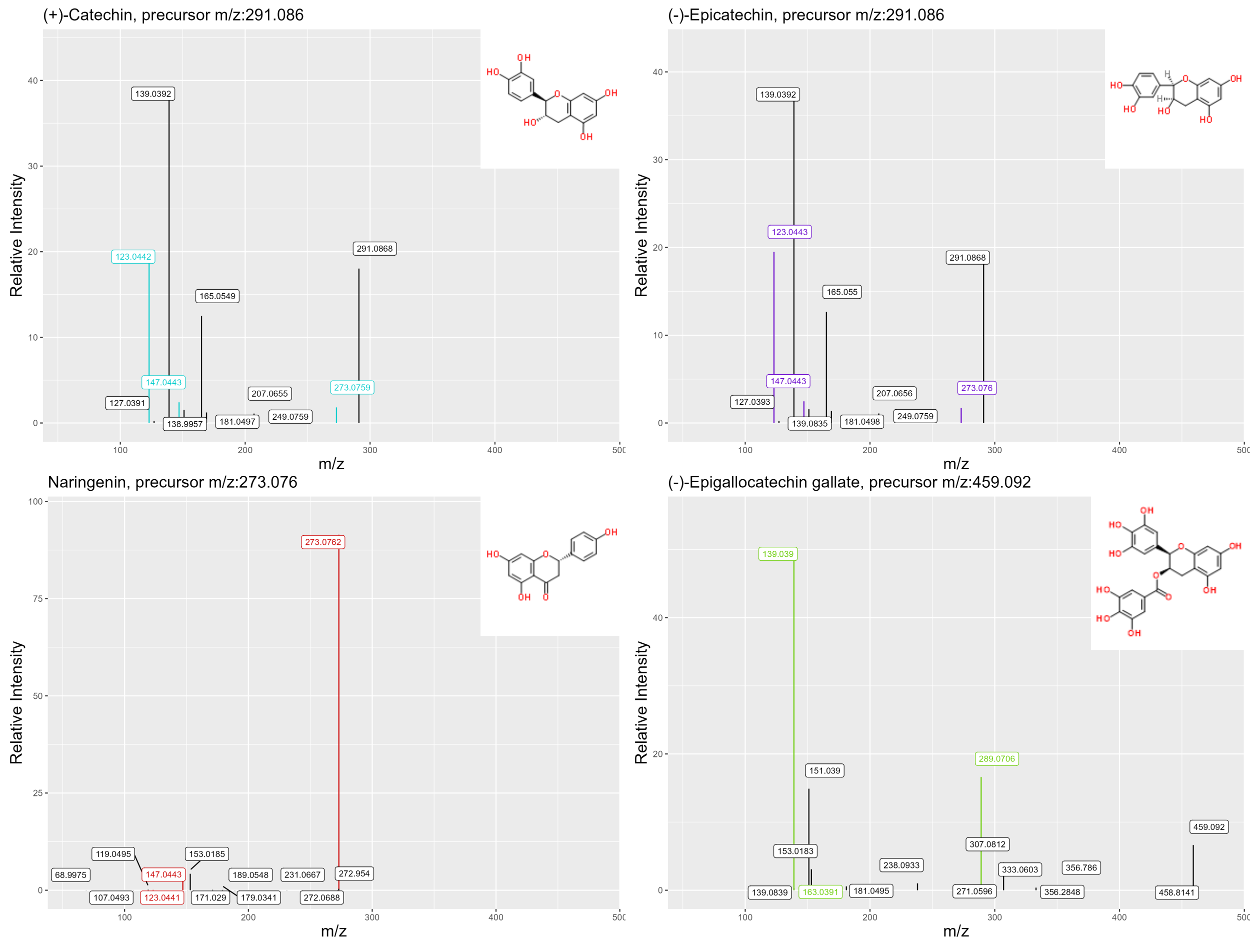

(a) MS/MS spectra

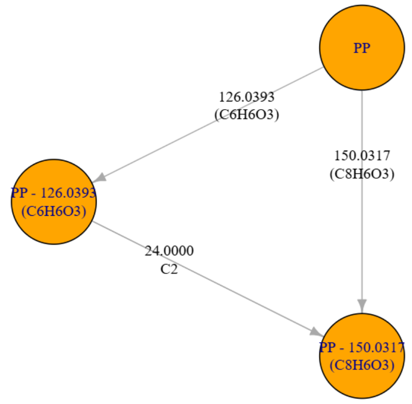

dmz	possible formulas
24.0000	$\boldsymbol{C_2}$
126.0393	$\boldsymbol{C_6H_6O_3}$, $C_2H_6O_6$, CH_2N_8, $C_{10}H_6$,$C_3H_{10}O_5$,$C_7H_{10}O_2$, $H_{14}O_7$,$C_4H_{14}O_4$
150.0317	$\boldsymbol{C_8H_6O_3}$, $C_4H_6O_6$,$CH_{10}O_8$, $C_{12}H_6$,$C_5H_{10}O_5$

(b) mineMS2 explaining pattern

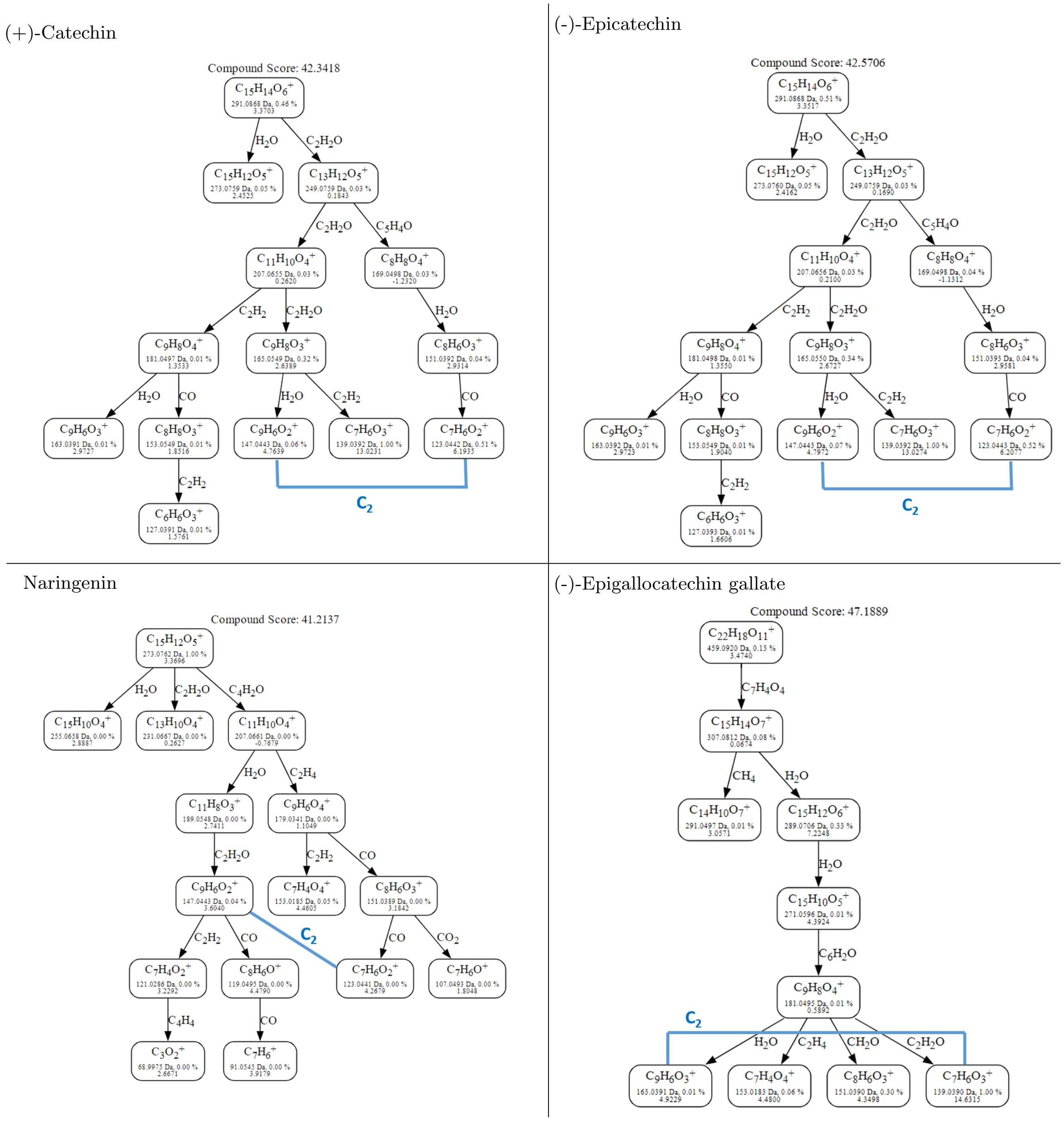

(c) Fragmentation trees obtained from the MS/MS spectra with SIRIUS [Böcker et al., 2009]

Figure S6: Correspondence between mineMS2 patterns and SIRIUS fragmentation trees for two ChemOnt concepts.

The mineMS2 pattern best explaining the *Methoxybenzoic acids and derivatives* (respectively, the *Methionine and derivatives*) concept are shown on the left at the top (respectively, at the bottom). Each pattern has a support consisting of three spectra, whose fragmentation trees computed by SIRIUS are shown on the right. The nodes from the fragmentation trees matching the mineMS2 pattern are colored.

Whereas the mineMS2 pattern from the *Methoxybenzoic acids and derivatives* concept is present in all SIRIUS fragmentation trees as a consistent fully connected subtree (top), the pattern from the *Methionine and derivatives* concept is not found in the fragmentation trees, as the red vertices are not connected in a similar manner (bottom).

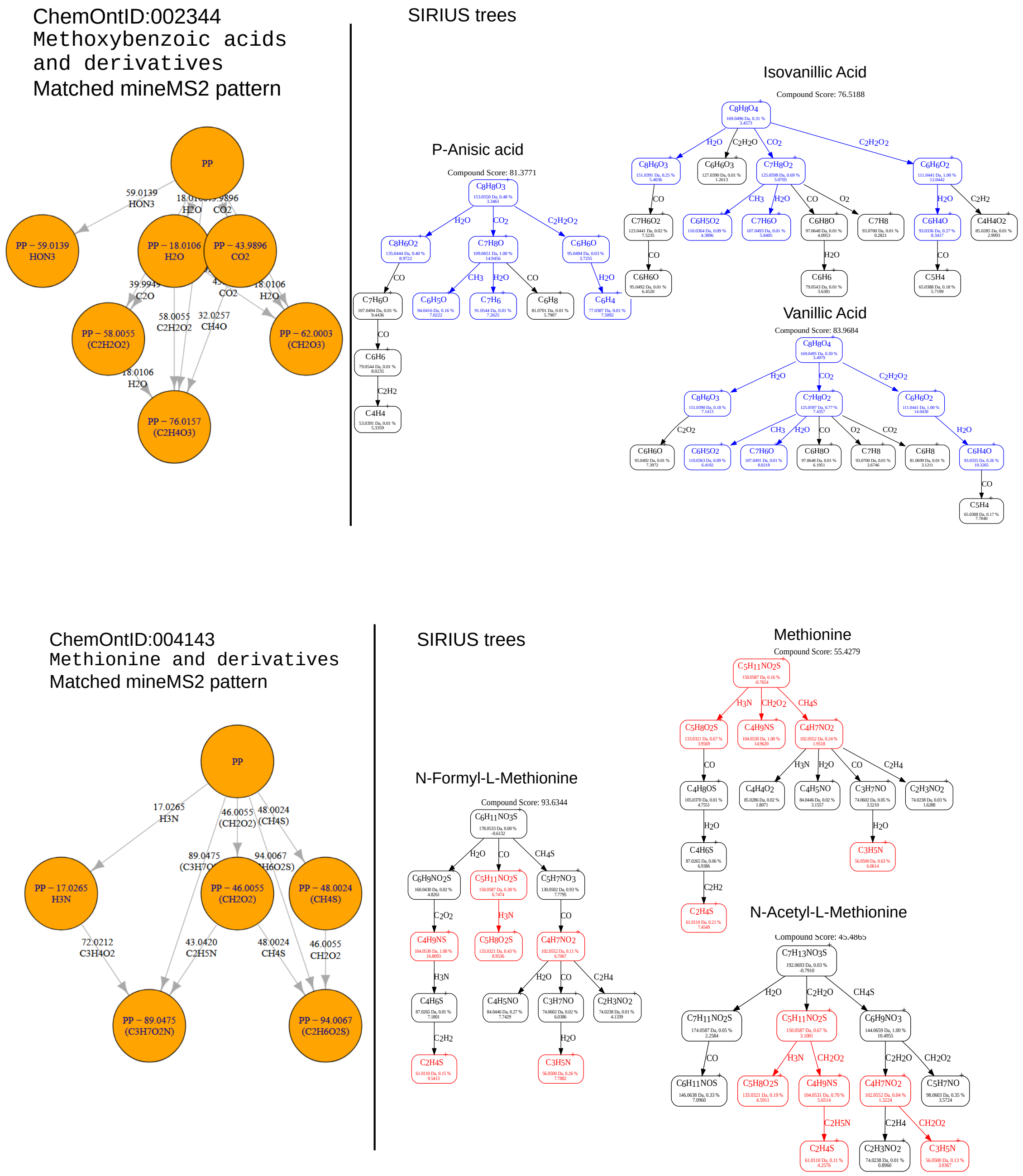

Figure S7: Proposed structures of unknown secondary metabolites detected at m/z 416, 391 and 393 in the *Penicillium* studies [Hautbergue et al., 2017, 2019].

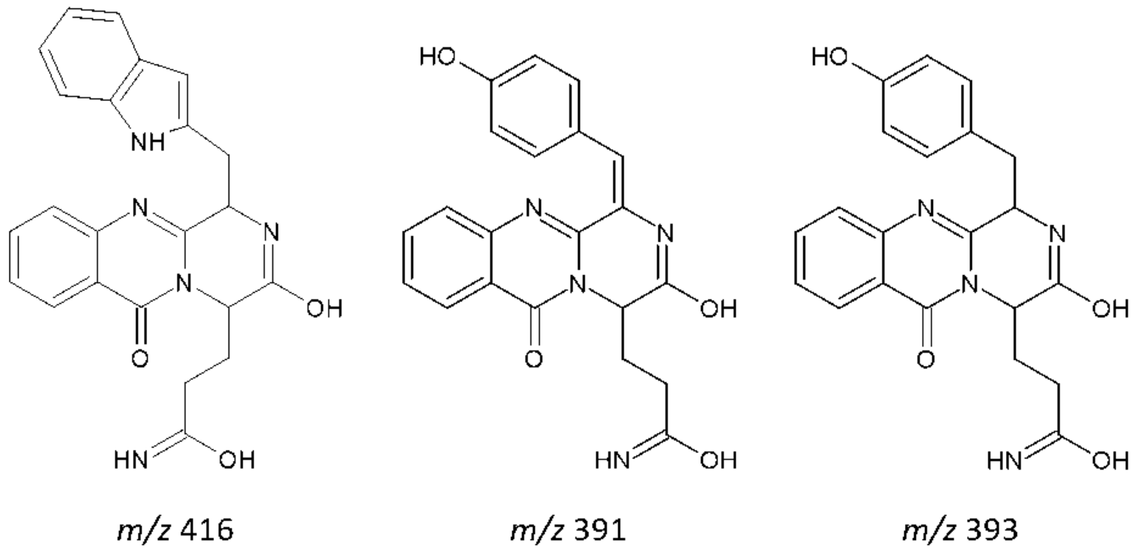

References

S. Böcker and Z. Liptak. A fast and simple algorithm for the money changing problem. *Algorithmica*, 48(4):413–432, Aug. 2007. doi: 10.1007/s00453-007-0162-8.

S. Böcker, M. C. Letzel, Z. Liptak, and A. Pervukhin. SIRIUS: Decomposing isotope patterns for metabolite identification. *Bioinformatics*, 25(2):218–224, 2009. doi: 10.1093/bioinformatics/btn603.

A. Dablanc, S. Hennechart, A. Perez, G. Cabanac, Y. Guitton, N. Paulhe, B. Lyan, E. L. Jamin, F. Giacomoni, and G. Marti. FragHub: A Mass Spectral Library Data Integration Workflow. *Analytical Chemistry*, 96(30):12489–12496, July 2024. doi: 10.1021/acs.analchem.4c02219.

T. Hautbergue, O. Puel, S. Tadrist, L. Meneghetti, M. Pean, M. Delaforge, L. Debrauwer, I. P. Oswald, and E. L. Jamin. Evidencing 98 secondary metabolites of Penicillium verrucosum using substrate isotopic labeling and high-resolution mass spectrometry. *Journal of Chromatography B*, 1071:29–43, 2017. doi: 10.1016/j.jchromb.2017.03.011.

T. Hautbergue, E. L. Jamin, R. Costantino, S. Tadrist, L. Meneghetti, J.-C. Tabet, L. Debrauwer, I. P. Oswald, and O. Puel. Combination of isotope labeling and molecular networking of tandem mass spectrometry data to reveal 69 unknown metabolites produced by penicillium nordicum. *Analytical Chemistry*, pages 12191–12202, Sept. 2019. doi: 10.1021/acs.analchem.9b01634.

T. Kind and O. Fiehn. Seven Golden Rules for heuristic filtering of molecular formulas obtained by accurate mass spectrometry. *BMC Bioinformatics*, 8(1):105, 2007. doi: 10.1186/1471-2105-8-105.

M. Wang, J. J. Carver, V. V. Phelan, L. M. Sanchez, N. Garg, Y. Peng, D. D. Nguyen, J. Watrous, C. A. Kapono, T. Luzzatto-Knaan, C. Porto, A. Bouslimani, A. V. Melnik, M. J. Meehan, W.-T. Liu, M. Crüsemann, P. D. Boudreau, E. Esquenazi, M. Sandoval-Calderón, R. D. Kersten, L. A. Pace, R. A. Quinn, K. R. Duncan, C.-C. Hsu, D. J. Floros, R. G. Gavilan, K. Kleigrewe, T. Northen, R. J. Dutton, D. Parrot, E. E. Carlson, B. Aigle, C. F. Michelsen, L. Jelsbak, C. Sohlenkamp, P. Pevzner, A. Edlund, J. McLean, J. Piel, B. T. Murphy, L. Gerwick, C.-C. Liaw, Y.-L. Yang, H.-U. Humpf, M. Maansson, R. A. Keyzers, A. C. Sims, A. R. Johnson, A. M. Sidebottom, B. E. Sedio, A. Klitgaard, C. B. Larson, C. A. Boya P, D. Torres-Mendoza, D. J. Gonzalez, D. B. Silva, L. M. Marques, D. P. Demarque, E. Pociute, E. C. O'Neill, E. Briand, E. J. N. Helfrich, E. A. Granatosky, E. Glukhov, F. Ryffel, H. Houson, H. Mohimani, J. J. Kharbush, Y. Zeng, J. A. Vorholt, K. L. Kurita, P. Charusanti, K. L. McPhail, K. F. Nielsen, L. Vuong, M. Elfeki, M. F. Traxler, N. Engene, N. Koyama, O. B. Vining, R. Baric, R. R. Silva, S. J. Mascuch, S. Tomasi, S. Jenkins, V. Macherla, T. Hoffman, V. Agarwal, P. G. Williams, J. Dai, R. Neupane, J. Gurr, A. M. C. Rodríguez, A. Lamsa, C. Zhang, K. Dorrestein, B. M. Duggan, J. Almaliti, P.-M. Allard, P. Phapale, L.-F. Nothias, T. Alexandrov, M. Litaudon, J.-L. Wolfender, J. E. Kyle, T. O. Metz, T. Peryea, D.-T. Nguyen, D. VanLeer, P. Shinn, A. Jadhav, R. Müller, K. M. Waters, W. Shi, X. Liu, L. Zhang, R. Knight, P. R. Jensen, B. Ø. Palsson, K. Pogliano, R. G. Linington, M. Gutiérrez, N. P. Lopes, W. H. Gerwick, B. S. Moore, P. C. Dorrestein, and N. Bandeira. Sharing and community curation of mass spectrometry data with Global Natural Products Social Molecular Networking. *Nature Biotechnology*, 34(8): 828–837, Aug. 2016. doi: 10.1038/nbt.3597.

X. Yan and J. Han. gSpan: Graph-based substructure pattern mining. In *2002 IEEE International Conference on Data Mining, 2002. Proceedings.*, pages 721–724, 2002. doi: 10.1109/ICDM.2002.1184038.

M. J. Zaki. Efficiently mining frequent trees in a forest. In *Proceedings of the eighth ACM SIGKDD international conference on Knowledge discovery and data mining*, KDD '02, pages 71–80, New York, NY, USA, July 2002. Association for Computing Machinery. ISBN 978-1-58113-567-1. doi: 10.1145/775047.775058.
